# Reprogramming triggers endogenous L1 and *Alu* retrotransposition in human induced pluripotent stem cells

**DOI:** 10.1038/ncomms10286

**Published:** 2016-01-08

**Authors:** Sabine Klawitter, Nina V. Fuchs, Kyle R. Upton, Martin Muñoz-Lopez, Ruchi Shukla, Jichang Wang, Marta Garcia-Cañadas, Cesar Lopez-Ruiz, Daniel J. Gerhardt, Attila Sebe, Ivana Grabundzija, Sylvia Merkert, Patricia Gerdes, J. Andres Pulgarin, Anja Bock, Ulrike Held, Anett Witthuhn, Alexandra Haase, Balázs Sarkadi, Johannes Löwer, Ernst J. Wolvetang, Ulrich Martin, Zoltán Ivics, Zsuzsanna Izsvák, Jose L. Garcia-Perez, Geoffrey J. Faulkner, Gerald G. Schumann

**Affiliations:** 1 Division of Medical Biotechnology, Paul-Ehrlich-Institute, D-63225 Langen, Germany; 2 Max-Delbrück-Center for Molecular Medicine, D-13125 Berlin, Germany; 3 Mater Research Institute, University of Queensland, TRI Building, Woolloongabba, Brisbane, Queensland 4102, Australia; 4 Department of Human DNA Variability, Pfizer/University of Granada and Andalusian Regional Government Center for Genomics and Oncology (GENYO), PTS Granada, 18016 Granada, Spain; 5 Leibniz Research Laboratories for Biotechnology and Artificial Organs (LEBAO), Department of Cardiac, Thoracic, Transplantation, and Vascular Surgery; REBIRTH, Cluster of Excellence, Hannover Medical School, D-30625 Hannover, Germany; 6 Department of Biophysics and Radiation Biology, Semmelweis University, H-1094 Budapest, Hungary; 7 Australian Institute for Bioengineering and Nanotechnology, The University of Queensland, St Lucia, Queensland 4072, Australia; 8 Queensland Brain Institute, University of Queensland, Brisbane, Queensland 4072, Australia

## Abstract

Human induced pluripotent stem cells (hiPSCs) are capable of unlimited proliferation and can differentiate *in vitro* to generate derivatives of the three primary germ layers. Genetic and epigenetic abnormalities have been reported by Wissing and colleagues to occur during hiPSC derivation, including mobilization of engineered LINE-1 (L1) retrotransposons. However, incidence and functional impact of endogenous retrotransposition in hiPSCs are yet to be established. Here we apply retrotransposon capture sequencing to eight hiPSC lines and three human embryonic stem cell (hESC) lines, revealing endogenous L1, *Alu* and SINE-VNTR-Alu (SVA) mobilization during reprogramming and pluripotent stem cell cultivation. Surprisingly, 4/7 *de novo* L1 insertions are full length and 6/11 retrotransposition events occurred in protein-coding genes expressed in pluripotent stem cells. We further demonstrate that an intronic L1 insertion in the *CADPS2* gene is acquired during hiPSC cultivation and disrupts *CADPS2* expression. These experiments elucidate endogenous retrotransposition, and its potential consequences, in hiPSCs and hESCs.

## Abstract (web summary)

Genetic and epigenetic abnormalities have been found to result from reprogramming of differentiated cells into human induced pluripotent stem cells (hiPSCs). Here, Klawitter *et al.* identify endogenous L1, Alu and SVA mobilization during reprogramming, highlighting the risk of insertional mutagens in hiPSCs.

## Graphical abstract

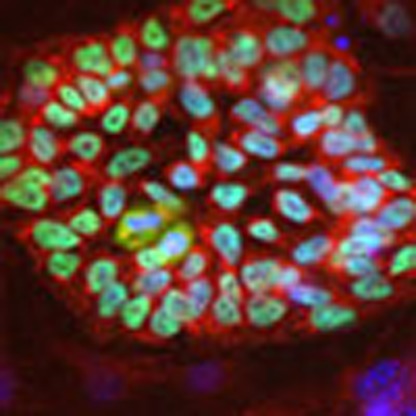

Human induced pluripotent stem cells (hiPSCs) hold substantial promise for biomedical applications and as *in vitro* models of disease and development. Unlike human embryonic stem cells (hESCs), hiPSCs are a potential source of autologous cells compatible with the immune system of transplant recipients^1^. hiPSCs also circumvent ethical issues associated with the use of human embryos^1^. However, genetic and epigenetic aberrations that occur during reprogramming and expansion *in vitro*^2^,^3^,^4^,^5^,^6^ may hinder the use of hiPSCs in regenerative medicine due to, for instance, an elevated risk of tumorigenesis upon implantation^7^. Thus, identifying the full spectrum of aberrant mutational processes occurring in the hiPSC genome, and their functional consequences, is of paramount significance.

LINE-1 (L1) retrotransposons (Fig. 1a) are mobile genetic elements remaining active in nearly all mammals^8^. In humans, 500,000 L1 copies contribute 17% of the genome, though only 80–100 L1s per individual remain transposition competent^9^,^10^,^11^. L1 mobilization is thought to primarily occur in germ cells and during early embryonic development and, together with L1-mediated *Alu* and SVA retrotransposition, has caused widespread genome structural variation in human populations^10^,^12^,^13^,^14^. *De novo* retrotransposition events can profoundly alter gene structure, expression and function, and drive pathogenesis^15^,^16^,^17^. Several intracellular defence mechanisms have consequently evolved to limit L1 mobility, including histone modifications and DNA methylation^8^,^18^.

Nonetheless, epigenome-wide remodelling^19^ coincident with reprogramming appears to enable L1 promoter hypomethylation and transcriptional activation in hiPSCs^20^,^21^. hiPSCs and hESCs also support low-level retrotransposition of an engineered L1 reporter^13^,^20^,^22^. These observations indicate that the molecular machinery and substrates required for L1 retrotransposition exist in pluripotent stem cells. However, genomic analyses of mouse- and human-derived iPSC populations have to date not identified endogenous L1 mobilization events^23^,^24^. It is therefore unclear whether endogenous L1-mediated mobilization occurs during reprogramming or hiPSC cultivation and, as a result, the potential significance of L1 insertional mutagenesis in hiPSCs remains unresolved. Here, we describe the dynamics of L1 expression associated with reprogramming, elucidate L1, *Alu* and SVA mobilization in hiPSCs, and use an exemplar *de novo* L1 insertion in *CADPS2* to demonstrate the potential impact of endogenous retrotransposition in pluripotent stem cells.

## Results

### Dynamic L1 activity in hiPSCs

To elucidate endogenous L1 mobilization associated with hiPSC reprogramming, we first assembled a panel of eight hiPSC lines and matched parental cells. Briefly, hiPSCs were derived from human fibroblasts and cord blood-derived endothelial cells (hCBECs) using several combinations of reprogramming factors, as well as integrating and non-integrating delivery systems (Table 1). Extensive characterization of these lines is described elsewhere^25^,^26^,^27^ or, as for hFF-iPS4 and hiPS-SB4, was performed here to confirm differentiation potential and expression of pluripotency markers (Supplementary Figs 1 and 2). Noting that genomic aberrations observed in hiPSCs may occur in small parental cell subpopulations and only rise to prominence after hiPSC cultivation^28^, we ensured that each hiPSC line used in this study was reprogrammed from a single somatic cell. This lessened the probability that heterogeneous genomic variants in parental cells could be erroneously called as *de novo* in descendant hiPSCs. As additional controls, we used three hESC lines as benchmarks of L1 expression and pluripotency (Table 1).

**Figure 1:**
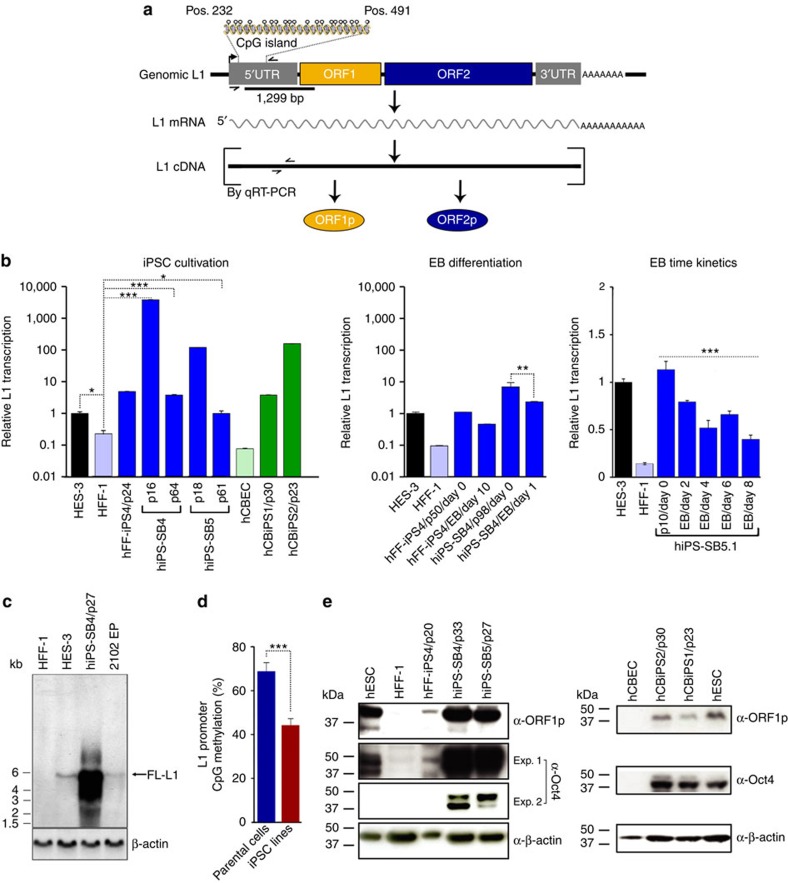
Reprogramming-induced expression of the L1 retrotransposition machinery is abrogated during embryoid body formation. (**a**) Schematic of organization and expression of a functional human L1 element. Binding sites of TaqMan primer/probe combinations (small convergent arrows) on L1 cDNA used for qRT–PCR analyses and of the 1,299-bp [α-^32^P]dCTP-labelled PCR product in the 5′UTR region (black bar) used for northern analysis are shown. Methylation status of the CpG island (position number 232–491 of the L1.3 reference sequence) was analysed. Open circles, CpG residues. (**b**) Relative full-length L1 (FL-L1) mRNA transcript levels were assessed by qRT–PCR from early passage (until p24) HFF-1-derived (hFF-iPS4, hiPS-SB4 and hiPS-SB5) and hCBEC-derived (hCBiPS1 and hCBiPS1) hiPSC lines (left panel), and after differentiation of hFF-iPS4 (p50) and hiPS-SB4 (p98) lines into embryoid bodies (EBs) (middle panel) (*P<0.05, **P<0.01, ***P<0.001). hiPS-SB5.1 cells (p10) were differentiated into EBs. L1 transcript levels were quantified on day 0 before initiation of differentiation, and after 2, 4, 6 and 8 days of differentiation by qRT–PCR (right panel; ***P<0.001, linear regression *t*-test). Bars represent arithmetic means±s.d. from experiments performed as technical duplicates of biological triplicates, or, in the case of hCBEC, hCBiPS1 and hCBiPS2 (green bars), arithmetic means of technical duplicates of one biological sample. (**c**) Northern analysis of cytoplasmic poly-A+ mRNA with a 1,299-bp L1 5′UTR-specific probe confirmed exceeding activation of FL-L1 transcription during hiPSC cultivation. β-Actin mRNA (1.8 kb, lower panel) served as loading control. (**d**) Endogenous L1 promoter sequences are significantly hypomethylated in hiPSC lines relative to their parental HFF-1 and hCBEC cells. Overall percentage methylation of 5′UTR CpG islands in HFF-1 and hCBEC cells (*n*=29 CpG islands; blue bar) and in five derived hiPSC lines (*n*=95 CpG islands; red bar), respectively, is presented. Error bars indicate s.e.m.***P<0.001; *χ*^2^ test. (**e**) Immunoblot analysis of cell lysates from HFF-1 and hCBEC cells and their respective derived hiPSC lines measures L1 ORF1p (40 kDa) and Oct-3/4 expression (A isoform, 45 kDa; B isoform 33 kDa). Shorter (exp.1) and longer exposures (exp.2) of the αOct-3/4 immunoblot are provided. Lysates from hESC lines HES-3 (left panel) and H1 (right panel) served as positive control for L1 ORF1p and Oct-3/4 expression. β-Actin (42 kDa) served as loading control.

**Figure 2:**
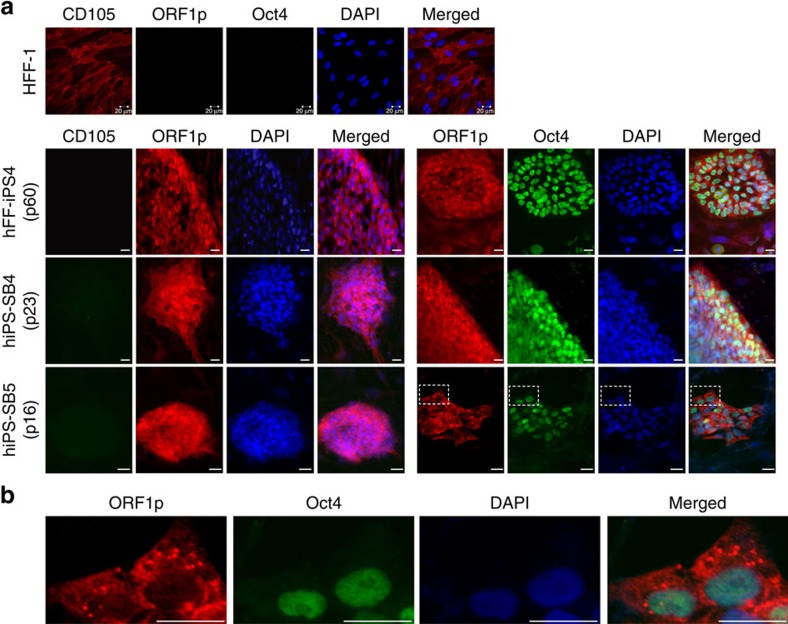
Immunofluorescence staining of hiPSC colonies and their parental cells for endogenous L1 ORF1p expression in HFF-1-derived hiPSCs. (**a**) ORF1p staining indicates activation of endogenous L1 expression after reprogramming of HFF-1 cells into lines hiPS-SB4, hiPS-SB5 and hFF-iPS4. Cells were analysed at passages (p) 23, 16 and 60, respectively. Oct-3/4 staining confirmed the pluripotent status of the analysed stem cell colonies. Mesenchymal stem cell marker CD105 (endoglin) is reported to be expressed in HFF-1 cells but not expressed in pluripotent stem cells. (**b**) Enlarged areas indicated by boxed dashes in **a** demonstrate cytoplasmic localization of endogenous L1 ORF1p and its accumulation in foci. Scale bars, 20 μm.

Transcription and translation of functional L1 elements are prerequisites for L1-mediated retrotransposition. To confirm that reports of pronounced L1 expression in hiPSCs by Wissing *et al.*^20^ could be extended to the hiPSC lines used in our study, we measured L1 mRNA abundance, L1 promoter methylation status and L1 ORF1 protein (ORF1p) expression in fibroblast (HFF-1)- and hCBEC-derived hiPSCs (hFF-iPS4, hiPS-SB4, hiPS-SB5, hCBiPS1 and hCBiPS2) and their parental cells (Table 1; Fig. 1a–d). TaqMan qRT–PCR (quantitative PCR with reverse transcription) targeting the L1 5′UTR (Fig. 1a; Supplementary Table 1) revealed significantly elevated L1 mRNA levels in each hiPSC line relative to their parental cells (*P*<0.05–*P*<0.0001, analysis of variance (ANOVA)), that peaked in earlier passages of cell lines hiPS-SB4 and hiPS-SB5 (Fig. 1b)^20^,^21^. Northern blot analyses with an L1 5′UTR-specific probe (Fig. 1a) confirmed elevated expression of full-length L1 transcripts in hiPSCs (Fig. 1c). Notably, extended hiPSC culture led to reduced L1 mRNA abundance (Fig. 1b, left panel; hiPS-SB4, hiPS-SB5; *P*<0.05–*P*<0.001, ANOVA) and resembled levels observed in hESCs (HES-3, Fig. 1b). Bisulfite DNA sequencing of the CpG island present in the canonical L1 promoter revealed strong hypomethylation in all tested hiPSC lines compared to parental cells (*P*_1,2_<2 × 10^6^, Fig. 1d; *P*_1_=2.6 × 10^–12^, *P*_2_=1.8 × 10^−5^, Supplementary Fig. 3; *χ*^2^ test). Consistently, L1 ORF1p was abundant in hiPSCs, based on immunoblot (Fig. 1e) and immunofluorescence assays (Fig. 2; Supplementary Fig. 4). In agreement with previous reports of cytoplasmic L1 ORF1p expression in human tumours and cancer cell lines^29^,^30^,^31^, in hiPSCs, we found L1 ORF1p predominantly expressed in cytoplasmic foci (Fig. 2b). However, unlike recent studies focused on other cell types^29^,^32^, we did not resolve whether L1 ORF1p was directed to stress granules in hiPSCs. Finally, quantitative immunoblot analyses (Supplementary Methods) revealed a tenfold increase in L1 ORF1p expression in hiPSCs when compared with parental cells (Supplementary Fig. 5).

Taken together, our results revealed a spike in L1 expression during or immediately after reprogramming, confirming previous findings^20^,^21^, followed by attenuation in later hiPSC passages (Fig. 1b,c). To extend these results, we measured L1 mRNA levels upon differentiation of late passage hiPSCs (hiPS-SB4 (p98) and hFF-iPS4 (p50)) into embryoid bodies. We observed 49% and 58% reductions in L1 mRNA levels after 1 and 10 days of embryoid body differentiation, respectively (Fig. 1b, middle panel). A parallel assay conducted with early passage hiPSCs indicated a gradual and significant decrease of L1 mRNA abundance by up to 65% after 8 days of embryoid body differentiation and a concomitant increase in differentiation markers (Fig. 1b, right panel; Supplementary Fig. 6). Hence, elevated L1 expression in hiPSCs was triggered by reprogramming and attenuated by short-term cultivation, while, in turn, subsequent differentiation gradually reduced L1 expression.

### Endogenous retrotransposition in pluripotent stem cells

To unambiguously determine whether activation of the L1 mobilization machinery produced L1-mediated retrotransposition, we used retrotransposon capture sequencing (RC-seq) to map the genomic integration sites of *de novo* retrotransposon insertions. Briefly, RC-seq involved liquid phase sequence capture to enrich DNA for the 5′ and 3′ junctions of recent L1, *Alu* and SVA insertions and the surrounding genome^33^. Putatively immobile long terminal repeat (LTR) retrotransposons were also probed as negative controls. Multiplexed, paired-end 150mer Illumina sequencing of RC-seq libraries, followed by contig assembly, provided high-fidelity, single nucleotide resolution of insertions absent from the reference genome, even at low read depth^33^.

We analysed all eight hiPSC lines and their matched parental cells by RC-seq. For five fibroblast-derived hiPSC lines (Table 1), we included two separate passages each to detect mobilization events that may have accumulated during cell culture. Similarly, we analysed two passages each of three hESC lines to evaluate endogenous retrotransposition during hESC cultivation (Table 1). RC-seq detected a total number of 40,608 non-reference retrotransposon insertions including on average 214 L1, 1,411 *Alu*, 53 SVA and 14 LTR non-reference genome insertions per hiPSC and hESC sample (Supplementary Fig. 7; Supplementary Data 1). Insertions were annotated as *de novo* in pluripotent cells if they were not (i) reported previously in non-reference retrotransposon insertion databases^9^,^12^,^33^,^34^,^35^,^36^,^37^,^38^, (ii) found in parental cells, (iii) found in an earlier hESC passage or (iv) found in multiple hiPSC or hESC lines. In total, we detected eight L1, seven *Alu* and two SVA putative *de novo* insertions (Supplementary Data 1). We found no *de novo* LTR retrotransposon insertions, despite observing profound upregulation of HERV-K group HML-2 transcription in hiPSCs and hESCs (Supplementary Methods; Supplementary Fig. 8).

**Figure 3:**
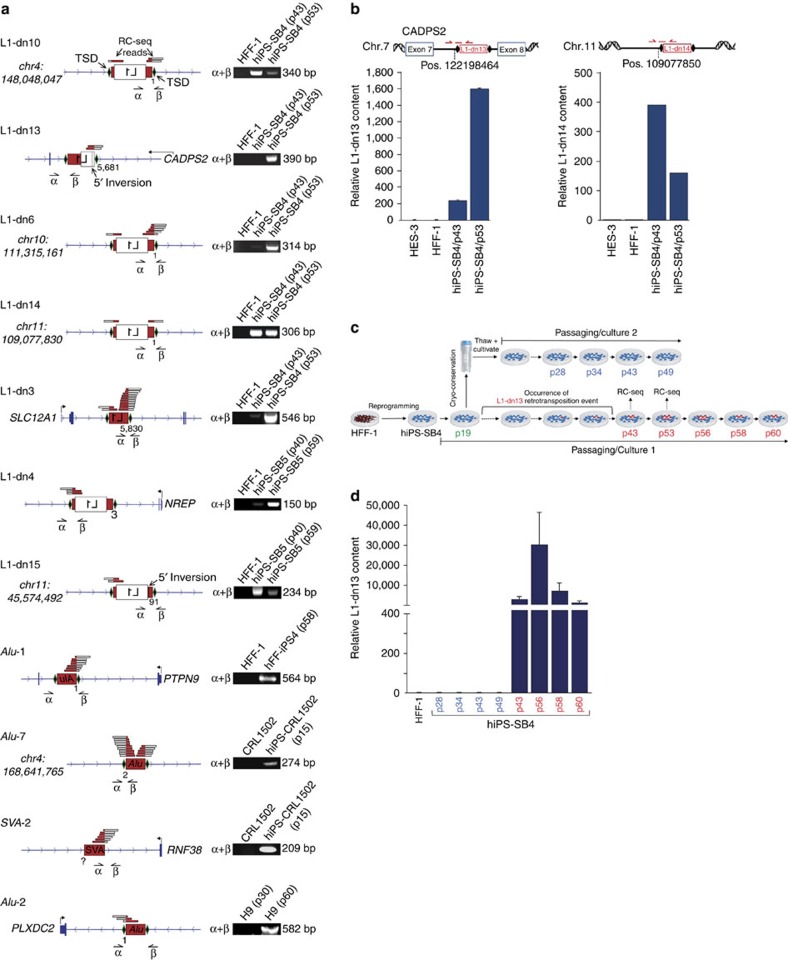
RC-seq reveals endogenous *de novo* L1, *Alu* and SVA retrotransposition in pluripotent stem cells. (**a**) Structures of validated *de novo* L1, *Alu* and SVA retrotransposition events (red box, untranslated region; white box, L1 ORF; green diamonds, TSDs). Names of insertions (for example, L1-dn10), and gene (for example, *SLC12A1*) or chromosomal positions for intergenic insertions are listed. RC-seq reads are aligned above the insertions (red/white bars). Nucleotide positions at 5′ ends of L1 and *Alu* insertions refer to L1.3 and *Alu*Yb8 reference sequences, respectively. Corresponding validation PCRs are presented on the right. α and β, validation primers. (**b**) Relative L1-dn13 and L1-dn14 copy numbers at hiPS-SB4 passages 43 and 53 were determined by qPCR. Binding sites of the TaqMan primer/probe combinations specific for the 5′ junctions of insertions L1-dn13 or L1-dn14 are shown (Top panels, red arrows and lines). Genomic DNAs from parental HFF-1 cells, and HES-3 cells served as negative controls. For normalization, a primer/probe combination specific for the human single-copy gene *RPP25* was used. ΔΔCt values measured the relative L1-dn13 and L1-dn14 insertion content, respectively, normalized to the parental cell line HFF-1. Bars, arithmetic means±s.e.m. of technical triplicates. Due to the minimal s.e.m. observed in the L1-dn14-specific qPCR (right panel), error bars are not visible. (**c**) Passaging scheme of the hiPS-SB4 line harbouring L1-dn13. After reprogramming of HFF-1 cells into the hiPS-SB4 line, hiPSCs were cultivated for 60 passages (culture 1). Genomic DNA (gDNA) was isolated from culture 1 at passages shown in red. Cells of passage 19 were split and half of the culture was cryo-preserved and cultivated again after several weeks of cryo-preservation (culture 2). gDNA was isolated from passages shown in blue. (**d**) Relative L1-dn13 content at passages 43, 56, 58 and 60 of culture 1 (red lettering) and at passages 28, 34, 43 and 49 of culture 2 (blue lettering) were quantified by qPCR. L1-dn13 is present in passages 43 to 60 of culture 1, but absent from culture 2.

Five retrotransposon subfamilies (L1-Ta, L1 pre-Ta, *Alu*Yb8, *Alu*Ya5 and SVA_E_) known to be active in humans contributed putative *de novo* insertions^10^,^11^,^39^. These were first validated by genotyping PCR, with seven L1, two *Alu* and one SVA insertion confirmed as *de novo* in hiPSCs and a single *Alu* insertion (*Alu*-2) in hESCs (Fig. 3a; Supplementary Figs 9 and 10; Supplementary Table 3). The remaining six putative *de novo* insertions (one L1, four *Alu* and one SVA) were detected by PCR in parental cells or an earlier hESC passage, suggesting that these variants were present but were not *de novo*. Next, we determined the entire nucleotide sequence of 10/11 confirmed *de novo* retrotransposon insertions (Supplementary Figs 9 and 10). For one event, SVA-2, a member of the SVA_E_ subfamily, we could sequence only the 3′ junction, which included a poly-A tail characteristic of L1-mediated *trans* mobilization (Fig. 3a; Supplementary Fig. 9). Our efforts to PCR amplify the matching 5′ junction of SVA-2 with multiple primer combinations, intended to detect a possible 5′ SVA truncation or a small proximal genomic deletion, were unsuccessful (see Methods). One reasonable explanation for this outcome was the occurrence of a large 5′ genomic deletion at the SVA-2 integration site, as reported previously^13^,^40^,^41^,^42^. Additional sequence analyses revealed that 9/10 of the remaining insertions exhibited the canonical hallmarks of L1-mediated target-primed reverse transcription^8^,^43^ including: (i) a target site duplication (TSD), (ii) a variable length L1 poly-A tail and (iii) an integration site resembling the L1 endonuclease target motif 5′-TTTT/AA-3′ (refs 44, 45; Fig. 3a; Supplementary Fig. 9). The one exception, insertion L1-dn4, was 3′ truncated within its poly-A signal and devoid of an L1 endonuclease motif, but nevertheless incorporated an 8-bp TSD. These features were consistent with L1 endonuclease-independent retrotransposition^46^. Insertions L1-dn6 and L1-dn14 presented one and two untemplated G nucleotides at their 5′ ends, respectively, as seen elsewhere^40^,^42^. Insertions L1-dn3, L1-dn4, L1-dn13 and L1-dn15 exhibited microcomplementarities of one to five nucleotides at their 5′ end, a structural feature reported previously for L1 integration sites^47^. L1-dn13 and L1-dn15 were also 5′ truncated and inverted, consistent with ‘twin-priming’^48^, and in one instance a 5′ inversion was displaced from the remaining L1 sequence by a 25-bp DNA fragment of unknown origin (L1-dn15). Thus, L1-mediated retrotransposition in hiPSCs and hESCs occurs via mechanisms described previously in mammalian cells.

The rate of L1-mediated retrotransposition occurring in pluripotent stem cells was difficult to accurately assess given the unknown genomic heterogeneity of each population. However, by estimating the sensitivity of RC-seq, we were able to determine the approximate L1 mobilization rate in hiPSCs. First, we identified that the overall RC-seq false positive rate was 1.5%, based on our recent PCR validation rate of 98.5% for insertions found by RC-seq in a cohort of hepatocellular carcinoma patients^33^ using the same detection thresholds as used here. Next, we determined that 88.5, 92.8, 88.3 and 89.8% of germline L1, *Alu*, SVA and LTR insertions, respectively, found in a parental cell line or early hESC passage were also detected in the matched hiPSC or later hESC passage, indicating an overall RC-seq false negative rate of 7.9%. To then model the sensitivity of RC-seq for *de novo* insertions, we randomly sampled each library and determined the fraction of the total germline events detected in that library as a function of sampling depth (Supplementary Fig. 11). At 50% library sampling depth (that is, modelling 50% variant allele fraction) 71.4%, 76.2%, 68.4% and 87.3%, respectively, of the germline L1, *Alu*, SVA and LTR insertions found in hiPSC lines were detected, dropping to 5.9%, 5.8%, 7.4% and 27.1% at 5% sampling depth. The estimated overall false negative rates at 50% and 5% variant allele fraction for *de novo* insertions detected in hiPSC lines were therefore 30.5% and 94.4%, respectively. These figures were similar for hESC lines (31.5 and 94.1%). Thus, we concluded that although RC-seq reliably detected high variant allele fraction retrotransposon insertions, a large pool of low variant allele fraction events may have been overlooked at the RC-seq thresholds used here. This would be particularly acute in the chosen hESC lines where, unlike iPSCs, cells had not undergone a recent population bottleneck *in vitro*. Using these parameters and the observed *de novo* L1 insertion counts, we estimated that hiPSC lines carried 3.7 *de novo* L1 insertions with allele frequencies ≥5%, on average, extrapolating to ∼1 *de novo* L1 insertion per cell (see Methods). However, the low number of insertions identified precluded similar estimates for hESC lines.

### hiPSC cultivation causes individual L1 copy-number variation

Our qualitative L1 insertion site validation PCR experiments (Fig. 3a) indicated that some *de novo* L1 insertions detected by RC-seq were absent from the earlier hiPSC passage surveyed and therefore may have arisen after reprogramming. To better establish the temporal dynamics of L1 retrotransposition in hiPSCs, we performed multiplex TaqMan qPCR incorporating a 5′ junction-spanning probe (Fig. 3b) to quantify L1-dn13 and L1-dn14 copy-number variation in hiPS-SB4. We observed an eightfold increase in L1-dn13 copy number upon extended cultivation (Fig. 3b, left panel) and a ∼two-fold decrease in L1-dn14 copy number (Fig. 3b, right panel), indicating the presence of two different hiPS-SB4 subpopulations carrying insertions L1-dn13 or L1-dn14, respectively, with opposite growth dynamics. L1-dn13 and L1-dn14 were not detected in hESCs (HES-3) or the parental fibroblast (HFF-1) population, again showing that L1-dn13 and L1-dn14 were *de novo* insertions. As the hiPS-SB4 line was cultivated from a single-cell-derived hiPSC clone, these data showed that either one or both of these insertions occurred during or after reprogramming, confirming our RC-seq and genotyping PCR data. To discriminate whether L1-dn13 arose during hiPSC reprogramming or cultivation, we thawed and extensively cultivated a passage (p19) of hiPS-SB4 isolated well before the later passages analysed by RC-seq (p43 and p53) (Fig. 3c). L1-dn13 was not detected by qPCR in this second hiPS-SB4 cultivar (Fig. 3d). Hence, L1-dn13 likely arose in the original hiPS-SB4 cultivar between p19 and p43. We concluded that cultivation of hiPSCs, and hESCs, as described above for the *Alu*-2 insertion (Fig. 3a), can lead to endogenous retrotransposition.

### *De novo* L1 insertions retain retrotransposition competency

**Figure 4:**
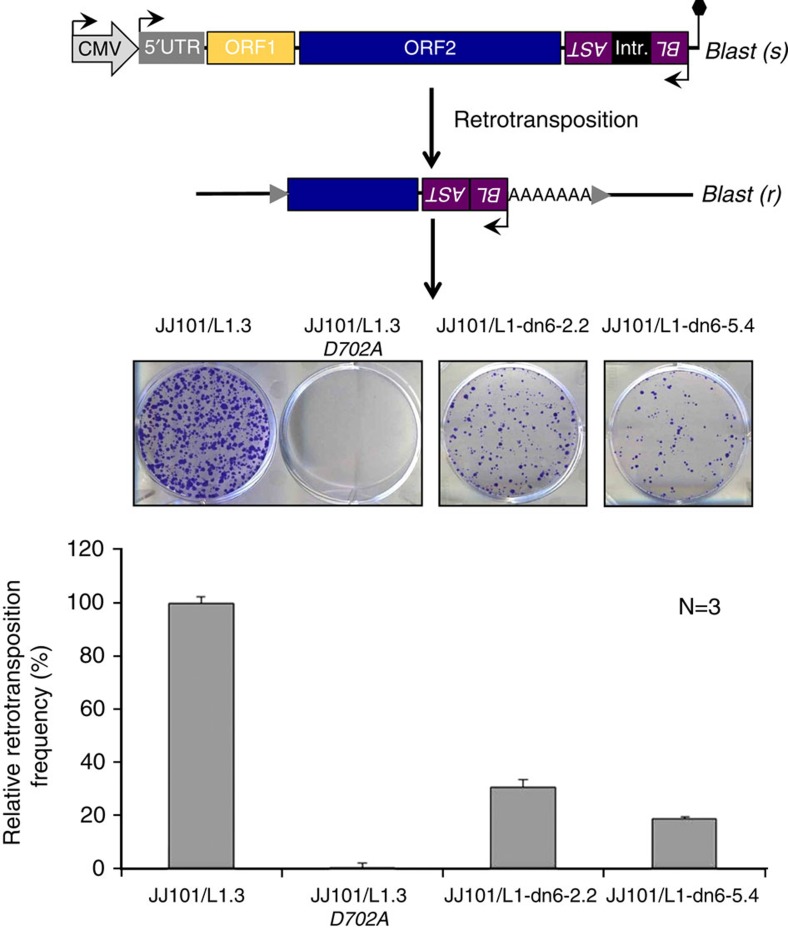
***De novo*** **full-length L1 insertions retain retrotransposition competency** ***in vitro.*** Intact, full-length L1 insertions L1-dn6-2.2 and L1-dn6-5.4 were obtained from two independent genomic PCR reactions amplifying the L1-dn6 *de novo* insertion, tagged with an *mblastI* retrotransposition indicator cassette, and inserted into an episomal expression plasmid where they were transcriptionally controlled by the CMV promoter. Resulting L1 reporter plasmids pJJ101/L1-dn6-2.2 and pJJ101/L1-dn6-5.4 were submitted to the L1 retrotransposition reporter assay (see Methods). HeLa cells were transfected with the L1-dn6 reporter plasmids or with positive and negative control L1 reporter plasmids pJJ101/L1.3 and pJJ101/L1.3-D702A, respectively. Blastidicin-S resistant cells arise only if engineered L1 retrotransposition has occurred. pJJ101/L1.3 was used for normalization (100% activity). pJJ101/L1.3-D702A contains a single point mutation in the L1 reverse transcriptase domain. The bar diagram depicts arithmetic mean±s.d. of three independent retrotransposition reporter assays of the engineered L1-dn6 elements relative to L1.3. Black hexagon, SV40 polyadenylation signal; grey arrows, TSDs flanking a 5′-truncated *de novo* L1 insertion. Blast(s), Blastidicin-S sensitive; Blast(r), Blastidicin-S resistant; SD, splice donor; SA, splice acceptor.

Intriguingly, 4/7 *de novo* L1 insertions were full length, a surprising result given that most preexisting genomic L1 retrotransposition events are 5′ truncated^49^. Indeed, only ∼15% of L1 copies in the reference genome and <1% of somatic L1 insertions found thus far in tumours are full length^33^,^50^,^51^. PCR amplification and sequencing of three full-length *de novo* L1s (L1-dn4, L1-dn6 and L1-dn14) revealed no deleterious nonsense mutations in their ORFs (Supplementary Fig. 10), suggesting each insertion likely retained retrotransposition competency. As a proof-of-principle, we used an established cell culture-based L1 retrotransposition reporter assay^52^ to evaluate the mobility of L1-dn6 in HeLa cells. L1-dn6 subclones retrotransposed at a relative efficiency of 20–30% of that obtained for the benchmark L1.3 (accession no. L19088.1)^53^ element (Fig. 4) and were therefore classified as highly active or ‘hot’^9^,^11^. These data indicated that new, full-length L1 insertions in hiPSCs could retain substantial competence in initiating further rounds of mobilization.

### L1 insertional mutagenesis disrupts *CADPS2* expression

Six *de novo* retrotransposition events mapped to introns of protein-coding genes. These included key factors in neuron (*CADPS2* and *NREP*) and nephron (*SLC12A1*) biology, as well as genes with established and predicted roles in cell cycle regulation and oncogenesis (*PTPN9*, *RNF38* and *PLXDC2*). Insertions showed a marked bias for the 5′ end of genes, with insertions falling on average in the 20th percentile of gene length measured from the annotated RefSeq transcription start site (TSS), a significant deviation from random expectation (*P*<0.006, permutation test). Albeit based on a small sample of insertions, this outcome could be explained by L1 endonuclease preference for open chromatin^54^ and increased chromatin accessibility around transcription start sites^55^.

**Figure 5:**
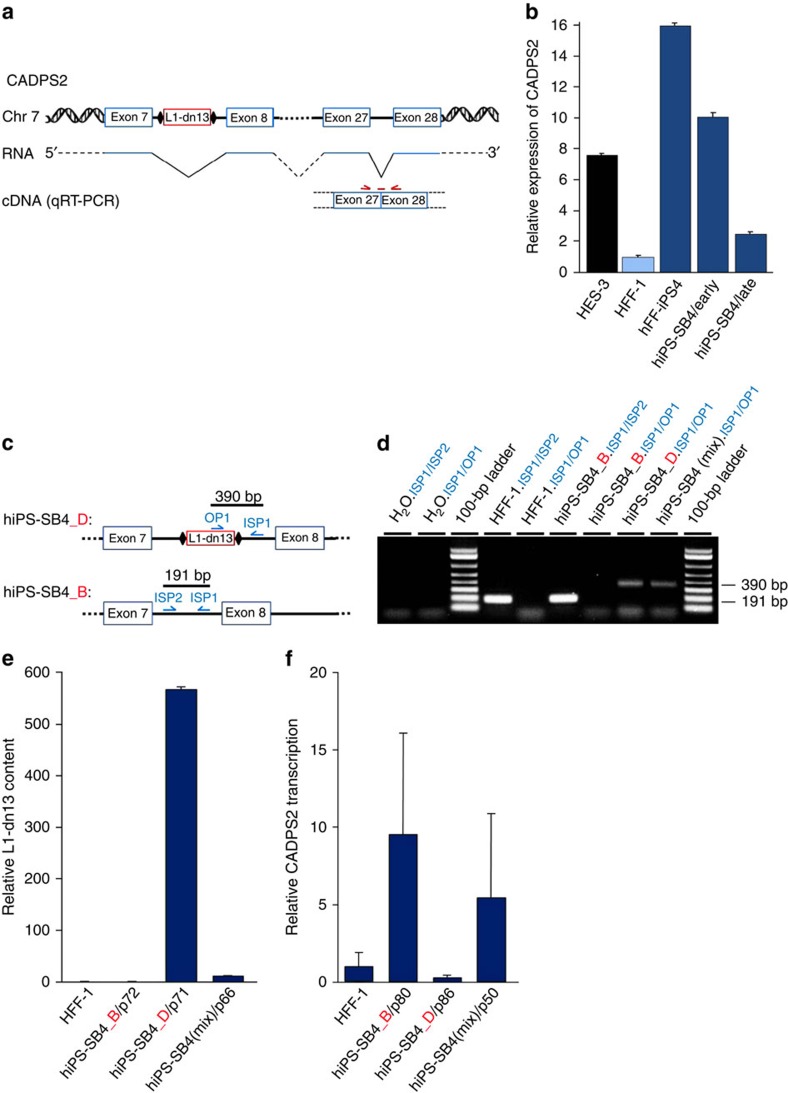
L1-dn13 affects *CADPS2* expression. (**a**) Schematic of the human *CADPS2* allele of the hiPS-SB4 line harbouring insertion L1-dn13. A *CADPS2* transcript including exons 7, 8, 27 and 28 is presented. Binding sites of the TaqMan primer/probe combination spanning the exon27/exon28 junction on *CADPS2* cDNA used for qRT–PCR analysis are shown (red arrows and line). (**b**) Relative CADPS2 mRNA levels in early (p16) and late passage (p50) hiPS-SB4 cells were assessed by qRT–PCR. HES-3 and hFF-iPS4 cells served as positive controls. qRT–PCR results were normalized to 18S rRNA using CADPS2 expression in parental HFF-1 cells as control. Bars, arithmetic means±s.e.m. of technical triplicates. (**c**) Structure of the L1-dn13 integration site in the *CADPS2* gene in hiPS-SB4 subclones. hiPS-SB4_D differs from hiPS-SB4_B by the presence of the L1-dn13 *de novo* insertion in *CADPS2* intron 7. Binding sites of L1-dn13-specific validation PCR primers OP1, ISP1 and ISP2 and expected lengths of the resulting PCR products are indicated. Black diamonds, TSDs. (**d**) Genotyping PCR validating the L1-dn13 presence in subclone hiPS-SB4_D and its absence from hiPS-SB4_B in gDNAs isolated from HFF-1, hiPS_SB4_B, and hiPS_SB4_D cells and from the original mixed population of the hiPS-SB4 culture (hiPS-SB4(Mix)). Primer combinations used are indicated in blue; H_2_O.ISP1/ISP2 and H_2_O.ISP1/OP1, negative control PCRs using H_2_O instead of gDNA; 100-bp ladder, size marker. (**e**) qPCR analyses confirming absence of L1-dn13 from hiPS-SB4_B and HFF-1 cells, and its presence in hiPS-SB4_D cells and the hiPS-SB4 culture. gDNAs from HFF-1 cells and from hiPS-SB4(Mix) cells served as negative and positive controls, respectively. For normalization, a primer/probe combination specific for the human *RPP25* gene was used. ΔΔCt values measured the relative quantity of L1-dn13. Bars, arithmetic means±s.e.m. of technical triplicates. (**f**) Relative *CADPS2* mRNA levels in hiPS-SB4_B, hiPS-SB4_D and hiPS-SB4(Mix) cells were determined by qRT–PCR using cytoplasmic RNA and primer/probe combinations spanning exon 27/exon 28 junction of *CADPS2*. Bars, arithmetic means±s.e.m. of technical triplicates.

Given that intronic L1 insertions can disrupt host gene transcription^8^,^15^,^56^, we noted with interest that all six genes were expressed in hiPSCs and hESCs^57^. For example, L1-dn13 occurred in an intron of *CADPS2* and, as noted above, exhibited copy-number variation during hiPSC cultivation (Fig. 3b,d). This afforded us an opportunity to analyse differential *CADPS2* expression with reference to L1-dn13 copy number. First, we measured and compared *CADPS2* mRNA expression in early versus late hiPS-SB4 passages via TaqMan qRT–PCR (Fig. 5a) and observed a fivefold reduction in *CADPS2* expression in the latter cells (Fig. 5b). Importantly, this assay tested *CADPS2* expression at an exon junction located downstream of the L1-dn13 integration site (Fig. 5a) and indicated opposing changes in L1-dn13 copy number (Fig. 3b, left panel) and *CADPS2* expression for hiPS-SB4 cells in culture, suggesting that L1-dn13 interfered with *CADPS2* expression.

To further test this possibility, we employed a human triose phosphate isomerase/Renilla luciferase reporter assay developed to monitor the effects of different introns on mammalian gene expression^58^. We generated three constructs (Supplementary Methods; Supplementary Fig. 12a,b) respectively containing: (i) 825 bp spanning the empty L1-dn13 target intron of *CADPS2* (pSHM06_01), (ii) 423 bp spanning the same region but in this case containing the 389 bp L1-dn13 insertion and its TSDs to produce a 825 bp sequence (pSHM06_02) and (iii) the 423 bp sequence on its own (pSHM06_03). We cloned each of these fragments into the triose phosphate isomerase/Renilla reporter cassette and quantified their effect on luciferase activity (Supplementary Fig. 12c). Interestingly, the *CADPS2* intron sequence harbouring L1-dn13 (pSHM06_03) had the strongest inhibitory effect and reduced luciferase activity by 62%, a significant decrease beyond the constructs lacking L1-dn13 (*P*=0.022).

As further corollary, we isolated two clones from the original hiPS-SB4 culture by single cell cloning (see Methods) where L1-dn13 was identified by RC-seq, one carrying L1-dn13 (hiPS-SB4_D) and the other not carrying L1-dn13 (hiPS-SB4_B) (Fig. 5c). The identity of each clone was verified by genotyping PCR (Fig. 5d) and qPCR (Fig. 5e). qRT–PCR applied to cytoplasmic RNA extracted from each clone indicated that *CADPS2* expression was ∼95% lower in hiPS-SB4_D than in hiPS-SB4_B (Fig. 5f). Consistently, *CADPS2* expression in the original hiPS-SB4 culture, which was heterogeneous for the L1-dn13 allele, was in between expression levels observed for the hiPS-SB4_D and hiPS-SB4_B clones. We then employed end point quantitative RT-PCR^59^ with subsequent capillary electrophoresis to compare the relative expression of each *CADPS2* allele in hiPS-SB4_D (Supplementary Methods; Supplementary Fig. 13), as distinguished by a single nucleotide polymorphism located in the 3′UTR of *CADPS2*. Notably, L1-dn13 was associated with complete silencing of the *CADPS2* mutant allele in hiPS-SB4_D while, interestingly, the *CADPS2* wild-type allele was also downregulated by >90% relative to hiPS-SB4_B. Again, expression of each *CADPS2* allele in the hiPS-SB4 culture heterogeneous for L1-dn13 lay between levels observed for the hiPS-SB4_B and hiPS-SB4_D subclones. Altogether, these results conclusively indicate that L1-dn13 interfered with *CADPS2* expression.

## Discussion

Here we have demonstrated that endogenous L1-mediated retrotransposition can occur in hiPSCs and hESCs, building upon earlier reports of engineered L1 retrotransposition in stem cells^13^,^20^,^22^,^60^. By contrast, two previous studies reported an absence of endogenous retrotransposition events in mouse or human iPSCs^23^,^24^. A more recent study reported low-level L1 mobilization in hiPSCs^61^, though in this case no insertions could be confirmed by PCR, leaving the validity of the reported putative L1 insertions unclear. We unequivocally demonstrated here by RC-seq, gold-standard PCR validation and capillary sequencing, including L1 integration site structural characterization, that fibroblast-derived hiPSCs clearly can support the mobilization of endogenous non-LTR retrotransposons. We speculate that our use of clonally derived hiPSCs, and the robustness of RC-seq in detecting somatic L1 insertions^33^,^34^, enabled us to discover retrotransposition events that may have otherwise remained undetected.

We estimated that hiPSCs each carried ∼1 *de novo* L1 insertion, with the notable caveat that this calculation was based on a small number of observed events. Nonetheless, this is a much lower rate than recently found for human hippocampal neurons and glia (13.7 and 6.5 somatic L1 insertions per cell, respectively)^62^. Our sensitivity calculations suggested that most *de novo* insertions with a variant allele fraction of <5% in hiPSC and hESC populations were overlooked by RC-seq at the detection thresholds used here, and these were not included in the above rate estimate. This is a major consideration in concluding whether parental cell type or choice of reprogramming vector affects endogenous retrotransposition activity in hiPSCs. Low frequency or subclonal retrotransposition may indeed occur in our hCBEC-derived hiPSC lines, that were reprogrammed via lentiviral systems, and escaped detection by RC-seq here. Therefore, we would propose that additional experiments are required to better define how these and other considerations (for example, cultivation protocol) affect L1 activity. Indeed, one explanation for the low number of insertions characterized in hESCs is that these cell populations were not clonally derived and were therefore likely to present more extensive genomic heterogeneity than hiPSCs. The lone *Alu* insertion found here in H9 cells is nonetheless the first endogenous retrotransposition event reported in hESCs, reinforcing evidence that L1-mediated mobilization can occur in early human development^13^,^63^.

L1 activity was highly dynamic during reprogramming and hiPSC cultivation. Parental cells, early hiPSC passages, later hiPSC passages and re-differentiated cells presented grossly different levels of L1 expression. As corroborated by RC-seq, genotyping PCR and qPCR, the majority of retrotransposition in hiPSCs likely took place during or immediately after reprogramming, where we observed a peak in expression of the L1 mobilization machinery. As a result, each detected variant could affect substantial hiPSC subpopulations. Interestingly, major induction of L1 mRNA and protein expression, far in excess of that seen in hESCs and neural stem cells^13^,^60^, was accompanied by a comparatively modest increase in L1 mobilization rate. Due to drastic epigenetic changes occurring upon reprogramming, it is possible that reprogramming *per se* may activate the expression of cellular L1 restriction factors such as APOBEC proteins^22^ and PIWIL2 (ref. 64). Consistently, APOBEC3B and PIWIL2 have been demonstrated to control engineered L1 retrotransposition in hiPSCs^22^,^64^. Thus, it is tempting to speculate that the cellular milieu of hiPSCs and hESCs may permit L1 upregulation but also limit L1-mediated mutagenesis.

That 4/7 of the *de novo* L1 insertions reported here were full-length was consistent with 2/3 of the engineered L1 *de novo* insertions characterized by Wissing *et al.* also being full-length^20^. This >50% incidence of full-length L1 *de novo* insertions in hiPSCs is unexpected as only ∼15% of L1 copies in the human reference genome and <1% of somatic L1 insertions identified in tumours are full length^33^,^36^,^50^,^51^. However, 7/7 engineered L1 retrotransposition events found in hESCs were recently reported to be significantly 5′ truncated^13^, suggesting that pluripotency factors common to hiPSCs and hESCs might not play any role in the observed overrepresentation of full-length *de novo* L1 insertions found in hiPSCs. The mechanism of L1 5′ truncation is not fully understood. On one hand, the preponderance of 5′ truncated L1 copies in the genome has long been explained by an inability of the L1 reverse transcriptase encoded by L1 ORF2p to copy the entire template L1 RNA, either due to premature dissociation of the L1 reverse transcriptase from its RNA or competition from an unknown cellular RNase that digests the L1 RNA before completion of reverse transcription^65^. Therefore, it is possible that hiPSCs provide a nuclear environment allowing a more stable association of the L1 reverse transcriptase with L1 RNA, or the L1 reverse transcriptase does not have to compete with a cellular RNAse which might be differentially expressed in hiPSCs. On the other hand, a recent study demonstrated that the DNA-damage-signalling protein ATM may control the length or number of *de novo* L1 insertions in human neural stem cells^66^. Thus, it is possible that subtle differences in the DNA repair mechanisms operating in hiPSCs and hESCs could be related to the high frequency of full-length L1 insertions characterized in hiPSCs.

Each *de novo* L1 insertion reported here integrated in a protein-coding gene expressed in pluripotent cells. In one case, we identified an L1 insertion (L1-dn13) that arose during hiPSC cultivation and integrated into an intron of the gene *CADPS2*. It remains to be determined whether acquisition of L1-dn13, and a concurrent reduction in *CADPS2* expression, imbued carrier hiPSCs with a selective advantage *in vitro*. Furthermore, it remains unclear why transcription of the *CADPS2* allele lacking L1-dn13 was reduced by >90%. To speculate, it is possible that *CADPS2* expression involves a direct or indirect positive feedback loop where, for example, transcription from *CADPS2* reinforces open chromatin^67^. A reduction in *CADPS2* expression caused by L1-dn13 could hence have a strongly negative effect on transcription from the wild-type *CADPS2* allele.

In closing, it is notable that intronic L1, *Alu* and SVA insertions can alter cellular phenotype and are associated with numerous instances of human disease^56^. Future in-depth experiments are however required to definitively establish whether endogenous retrotransposition alters the phenotype of hiPSC derivatives sufficiently to impact their use in medical or research applications. We can nevertheless conclude that retrotransposition, in addition to other sources of genetic and epigenetic variation^2^,^3^,^4^,^5^,^6^, can change the functional landscape of the hiPSC genome.

## Methods

### Cell lines and culture conditions

hiPSC lines hiPS-SB4 (hFF-T2-OSKM) and hiPS-SB5 (hFF-T2-OSKML)/hiPS-SB5.1 (hiPS-OSKML#6) were generated by reprogramming HFF-1 cells (ATCC-Number: SCRC-1041) using Sleeping Beauty (SB) transposon-based plasmids pT2-OSKM or pT2-OSKML which contain polycistronic OSKM (*OCT4, SOX2, KLF4* and *c-myc*) or OSKML (OSKM+*LIN28*) expression cassettes^26^. Briefly, HFF-1 cells (4 × 10^5^ cells per well) were transfected by nucleofection (Lonza) according to the manufacturer’s instructions. In each transfection, 2 μg of transposon plasmid (pT2-OSKM or pT2-OSKML) and 0.2 μg of CMV- SB100X vector (harbouring the enhanced Sleeping Beauty transposase gene under control of a CMV promoter^26^) were used. After transfection cells were plated onto Matrigel-coated six-well plates (hESC-qualified Matrix, BD Biosciences) and were grown in MEF-conditioned ESC medium used for the cultivation of hESCs and hiPSCs. ESC medium consisted of Knockout DMEM (Life Technologies) supplemented with 4 ng ml^−1^ basic fibroblast growth factor 2 (FGF2, Invitrogen), 20% Knockout Serum Replacement (Gibco), 1 mM L-glutamine (Biochrom AG), 50 μM β-mercaptoethanol and 0.1 mM nonessential amino acids. The medium was replaced every day. Newly formed hiPSC colonies were picked, transferred to Matrigel-coated 24-well plates, and expanded for 4–6 days in MEF-conditioned ESC medium. Subsequently, cells were trypsin dissociated, plated onto feeder cells and cultivated in ESC medium.

In this experiment, cells nucleofected with SB-OSKM gave rise to only one hiPSC colony. Nucleofection with SB-OSKML resulted in several hiPSC colonies. Multiple SB-OSKML colonies were picked and transferred onto the same Matrigel-coated wells. After establishing a mixed culture of hiPSCs generated with either SB-OSKM or SB-OSKML, single-cell-derived hiPSC clones were generated by single-cell dilution using cell sorting (see below) based on their positivity for SSEA4. Six SB-OSKML hiPSC clones and one SB-OSKM hiPSC clone were then characterized for pluripotency and differentiation potential as described^26^. Two SB-OSKM hiPSC clones (hiPS-SB5 and hiPS-SB5.1) and the only SB-OSKM hiPSC clone (hiPS-SB4) obtained were used in this study.

The lines hiPS-CRL1502 (ref. 25), hiPS-CRL2429 (ref. 25), hCBiPS1 (ref. 27), hCBiPS2 (ref. 27) and hiPS-FB^68^ have been described previously. hFF-iPS4 was produced using HFF-1 cells and a lentiviral vector expressing reprogramming factors Oct-4, Sox2, Nanog and Lin28 (ref. 27). Successful reprogramming for the hFF-iPS4 cell line was verified by morphology, pluripotency marker expression (Supplementary Fig. 2), karyotype analysis and the ability to generate teratomas on immunocompromised mice.

hESC lines H1, H9 and HES-3 were purchased from the WiCell Research Institute (Madison, WI, USA) and Cythera Inc. (San Diego, CA, USA). The H1 line was used exclusively for the isolation of cell lysate that was loaded as positive control of the immunoblot analysis in Fig. 1e, right panel. hESC line HESG (GENEA23) was purchased from GENEA Biocells (http://www.geneastemcells.com.au). It formed well-defined colonies with compact cells displaying a high nuclear to cytoplasmic ratio and prominent nucleoli. Karyotype analysis (46 chromosomes, XY male) did not uncover any abnormalities at passage 42. HESG cells express pluripotency markers Nanog, Oct-4, Tra1-60 and SSEA4, stain positive for alkaline phosphatase and form teratomas. As for hiPSCs, hESCs were grown on gelatin-coated six-well plates (Greiner) on inactivated mouse embryonic fibroblasts (MEFs, passage 3, strain CF1; Merck Millipore, Catalogue Number: PMEF-CFL). MEFs were expanded and mitotically inactivated by γ-irradiation with a Cesium source with 30 Gy after 3–7 passages, and stored in liquid nitrogen until further use. After thawing, MEFs were seeded at a density of 6 × 10^5^ cells per well of a six-well plate. hESC medium was replaced daily and cells were passaged at a 1:2 dilution every 5 days using splitting medium (1 mg ml^−1^ collagenase IV (Gibco, Darmstadt, Germany) in KO-DMEM).

### Cell sorting

hiPSCs were washed once in PBS containing 0.5% bovine serum albumin, and incubated for 30 min with allophycocyanin-conjugated anti-human SSEA4 antibody (R&D Systems). In all samples an anti-mouse Sca-1 (Ly-6A/E) (FITC or PE conjugated, BD Pharmingen) antibody was employed, for gating out the positively labelled mouse feeder cells. Samples were analysed and sorted using an Aria High Speed Cell Sorter (Becton-Dickinson).

### Differentiation of hiPSCs into embryoid bodies and RNA extraction

In all experiments, hiPSCs grown on MEFs were detached from the feeder layer by adding 250 μl Collagenase Type IV (1 mg ml^−1^; Gibco) per well of a six-well tissue culture plate. Next, cells were resuspended in 750 μl of ESC medium, transferred to a 15 ml conical tube and centrifuged at 800 r.p.m. in a Heraeus Multifuge 4KR for 3 min at room temperature. Subsequently, medium was removed, cells were resuspended in 3 ml of ESC medium without FGF2 and cultured for 1–16 days in T25 flasks (Greiner) containing 10 ml of ESC medium without FGF2. At the indicated time, embryoid bodies were harvested and cytoplasmic RNA was isolated as described below. Passage 10 of the hiPSC line hiPS-SB5.1 was cultured in one well of a GeltrexTM-coated six-well culture dish, and treated with collagenase IV (1 mg ml^−1^) for 5 min. Cells were washed with warm PBS twice, and fed with 1 ml embryoid body formation medium (Knockout DMEM, 20% Knockout Serum Replacement, 1 mM L-Glutamine, 1% nonessential amino acids, 0.1 mM β-mercaptoethanol and Primocin (Invivogen)) and split into small cell clumps. hiPSC colonies were then dissociated with collagenase IV (1 mg ml^−1^) for 5 min, and split into small cell clumps. Cell clumps were transferred into three 10-cm low-attachment dishes and fed with embryoid body medium. The medium was changed every 2 days. Embryoid bodies were cultured for 8 days in total. Embryoid bodies were collected by sedimentation under gravity from three dishes on day 0 (undifferentiated hiPSCs), 2, 4, 6 and 8, respectively (Fig. 1b, right panel; Supplementary Fig. 6). Total RNA was extracted from each well using Trizol (Invitrogen) following the instructions of the manufacturer.

### Analysis of expression in embryoid bodies by qRT–PCR

To analyse the expression of both pluripotency markers and L1, real-time quantitative RT–PCR was applied. To this end, 0.1 μg total RNA per well was used for reverse transcription by using the High Capacity RNA-to-cDNA kit (Applied Biosystems). For each time point and transcript to be quantified, qRT–PCR analyses were done in triplicate. qRT–PCR for pluripotency/differentiation markers was carried out using Power SYBR Green PCR Master Mix (Applied Biosystems) on the ABI7900HT sequence detector (Applied Biosystems), and data was normalized to GAPDH expression. qRT–PCR for L1 was performed with ABsolute QPCR Mix (ABgene), and data was normalized to 18S rRNA expression.

### qRT–PCR using TaqMan fluorogenic probes

Cytoplasmic RNA was extracted from 5 × 10^6^ to 3 × 10^7^ somatic cells, hiPSCs or embryoid body cells using the RNeasy Midi Kit (Qiagen, Hilden, Germany) according to the manufacturer’s instructions. Cytoplasmic RNA (0.5–1 μg) was incubated with 2 U of RNAse-free DNaseI (Life Technologies, Darmstadt, Germany) for 30 min at room temperature. DNAseI digestion was stopped by adding 2 μl of 25 mM EDTA and incubation for 10 min at 65 °C. DNAseI-digested cytoplasmic RNA (0.1–0.5 μg) was used for cDNA synthesis using the SuperScript III First-Strand Synthesis Kit (Invitrogen) in combination with a Random Hexamer Primer (0.5 μg μl^−1^; Invitrogen) according to the manufacturer’s instructions. Quantitative real-time PCR was carried out in ABgene plates using an Applied Biosystems 7900HT Fast Real-Time PCR System. The primer and probe combination L1 5′UTR#2 (ref. 60) was used to quantify transcripts expressed from endogenous L1-Ta copies. Sequences of oligonucleotides and probes used for qRT–PCR are listed in Supplementary Table 1. The probe specific for the L1 5'UTR was labelled with the reporter fluorochrome 6-carboxy-fluorescein (FAM) and a non-fluorescent quencher. 18S rRNA expression was quantified using Eukaryotic 18S rRNA endogenous control (VIC/TAMRA Probe, Primer Limited; Part number 4310893E, Applied Biosystems). Transcript levels of the human *CADPS2* gene were monitored using a gene specific assay (Life Technologies, Hs00604528_m1) spanning exon sequences (Fig. 5a). Cycling conditions were the following: 95 °C for 15 min (one cycle), 95 °C for 15 s and 60 °C for 1 min (40 cycles). A total of 1–5 μl of cDNA per sample were used for the quantification of endogenous L1 and CADPS2 mRNA levels. Analysis of real-time and end point fluorescence was performed using the software SDS version 2.3 as well as RQ manager 1.2 (Applied Biosystems).

### Northern blot analysis

Total RNA was isolated from the cell lines HFF-1, 2102Ep (ref. 69), HES-3 and hiPS-SB4 using TRIzol (Invitrogen) according to the manufacturer’s instructions. Poly(A)+ RNA was isolated applying the Dynabeads mRNA Purification Kit (Life Technologies) according to the manufacturer’s instructions. Denatured mRNA (2.8 μg) from each cell line was subjected to denaturing electrophoresis in a horizontal 1% agarose gel containing morpholinepropanesulfonic acid buffer and 6% formaldehyde, and transferred onto a Hybond-N^+^-Nylon membrane (Amersham) by overnight capillary transfer using 10 × SSC as transfer buffer. A total of 4 μl RiboRuler High Range RNA ladder (MBI Fermentas, St.Leon-Rot, Germany) were loaded as size marker. After crosslinking the RNA onto the membrane by ‘baking’ at 80 °C for 2 h, the membrane was prehybridized overnight in 50% Formamide/4xSSC/1%SDS/2 × Denhardt’s at 42 °C. The full-length L1 mRNA-specific probe was generated by PCR amplification of a 1299-bp L1 fragment ranging from position numbers (pos.) 58–1356 of a full-length L1 element by using primers L1_FW1 and L1_RV1 (Supplementary Table 1) and pJM101/L1_RP_ΔCMV^70^ as template. Pos. refer to the L1.3 element^53^ sequence (accession number L19088.1). A 491-bp β-actin mRNA-specific probe was generated by PCR amplification using primers actin_FW and actin_RV_(Supplementary Table 1) and plasmid 31502 (Addgene^71^) as template. PCR fragments were labelled with [α-^32^P]dCTP by applying the Nick Translation System (Invitrogen) according to the manufacturer’s instructions.

After denaturing the probe for 10 min in boiling water and subsequent incubation for 10 min in ice water, the probe was added to the hybridization buffer (50% Formamide/4 × SSC/1% SDS/1 × Denhardt’s) and the membrane was incubated in the probe-containing hybridization buffer overnight at 42 °C. Subsequently, the membrane was subjected to two 5 min low-stringency washes (2 × SSC) at room temperature and one 30 min high-stringency wash (2 × SSC/0.5%SDS) at 65 °C. The membrane was stripped by being boiled for 30 min in a solution of 10 mM tris-HCl (pH 7.5)/1 mM EDTA/1 mM SDS. The hybridized membrane was exposed to X-ray films for 5–10 days with intensifying screens.

### Bisulfite DNA sequencing analyses

Bisulfite DNA sequencing analyses were performed as previously described^20^,^60^. Briefly, genomic DNA from hiPSCs and parental cells was isolated at the indicated passage using DNAzol Genomic DNA Isolation Reagent (MRC Inc, Cincinnati, OH, USA) according to the manufacturer’s instructions. Next, 2 μg of genomic DNA were bisulfite converted using an EpiTect Bisulfite Kit (Qiagen, Hilden, Germany) following manufacturer instructions, with a conversion efficiency of ∼95%. To determine the DNA methylation status of L1-Ta promoters, we performed PCR sequencing using primers L1-FW2: 5′-AAGGGGTTAGGGAGTTTTTTT and L1-RV2: 5′-TATCTATACCCTACCCCCAAAA. To this end, 300–500 ng of converted genomic DNA were used in a 50 μl PCR reaction as follows: 2 min at 95 °C, 35 cycles of 30 s at 94 °C followed by 30 s at 54 °C and 60 s at 72 °C, and a final extension of 10 min at 72 °C. Amplified products were gel purified (QIAquick gel extraction kit, Qiagen), cloned in pGEM-T Easy (Promega) and at least 30 individual clones were sequenced for each sample. The unique sequence in each clone was analysed using Repeatmasker at http://www.repeatmasker.org/cgi-bin/WEBRepeatMasker. Next, the fraction of unmethylated CpG sites was calculated by comparison to a consensus L1-Ta sequence. In addition, each individual sequence was compared to L1.3 and only the sequences with the highest homology to this sequence were used to plot methylation data in single clones (Supplementary Fig. 3d). The proportion of CpG converted to TpG by bisulfite treatment was compared between samples using the *χ*^2^ test (d.f.=1; *α*=0.05).

### Immunoblot analysis

hiPSC colonies were detached from their tissue culture dish by incubation with 250 μl of a 1 mg ml^−1^ collagenase type IV/DMEM and washed subsequently in 1 × PBS. Cells were spun down, resuspended in lysis buffer (50 mM Tris-HCl (pH 7.4), 150 mM NaCl, 10% Glycerin, 1% Triton X-100; 2 mM EDTA, 2 mM EGTA, 40 mM β-Glycerolphosphate disodium salt hydrate, 50 mM NaF, 10 mM Na_4_P_2_O_7_, 200 μM Na_3_VO_4_, 2 mM DTT; 1 × complete protease inhibitor cocktail (Roche Applied Science)), homogenized by passing the lysate ten times through a 26 G needle, and lysates were cleared by centrifugation. A total of 50 μg of each protein lysate were boiled in 3 × SDS sample buffer (NEB), loaded on 4–12% Bis/Tris gels (Invitrogen), subjected to SDS–polyacrylamide gel electrophoresis, and electroblotted onto nitrocellulose membranes. After protein transfer, membranes were blocked for 2 h at room temperature in a 10% solution of non-fat milk powder in 1 × PBS-T (137 mM NaCl, 3 mM KCl, 16.5 mM Na_2_HPO_4_, 1.5 mM KH_2_PO_4_, 0.05% Tween 20 (Sigma-Aldrich Chemie GmbH, Mannheim, Germany)), washed in 1 × PBS-T, and incubated overnight with the respective primary antibody at 4 °C.

L1 ORF1p and Oct-4 proteins were detected using the polyclonal rabbit-anti-L1 ORF1p antibody #984 (ref. 41) at a 1:2,000 dilution and the Oct-3/4 (C10) antibody (sc-5279, Santa Cruz Biotechnology Inc., Santa Cruz, CA, USA) at a 1:750 dilution, respectively, in 1 × PBS-T containing 5% milk powder as primary antibodies. Subsequently, membranes were washed thrice in 1 × PBS-T. As secondary antibodies, we used HRP-conjugated donkey anti-rabbit IgG antibody at a 1:30,000 dilution to detect L1 ORF1p, and HRP-conjugated donkey anti-mouse IgG antibody at a 1:10,000 dilution (Amersham Biosciences) to detect Oct-3/4, in 1 × PBS/5% milk powder and incubated the membrane for 2 h. Subsequently, the membrane was washed thrice for 10 min in 1 × PBS-T. β-Actin expression was detected using a monoclonal anti-β-actin antibody (clone AC-74, Sigma-Aldrich Chemie GmbH, Steinheim, Germany) at a dilution of 1:30,000 as primary antibody and an anti-mouse HRP-linked species-specific antibody (from sheep) at a dilution of 1:10,000 as secondary antibody. Immunocomplexes were visualized using lumino-based ECL immunoblot reagent (Amersham Biosciences Europe GmbH, Freiburg, Germany). Details of the applied antibodies are listed in Supplementary Table 2. Full scans of immunoblots are presented in Supplementary Fig. 14.

### Immunofluorescence staining

hiPSCs as well as their parental HFF-1 or hCBEC cells were grown on glass cover slips in 12-well plates. Cells were washed with 1 × PBS, fixed with 4% paraformaldehyde in 1 × PBS (pH 7.4) for 15 min at room temperature and permeabilized with 1% Triton X-100 (Sigma) in 1 × PBS for 10 min at room temperature. Subsequently, cells were washed thrice for 2 min in 1 × PBS. Cells were blocked by incubation with 5% (w/v) BSA/0,1% Triton X-100/1 × PBS (pH 7.4) for 30 min at room temperature followed by incubation with the respective primary antibodies, which are listed in Supplementary Table 2, for 1 h at room temperature in 5% BSA/1 × PBS (pH 7.4). Subsequently, cells were washed three times with 1 × PBS for 5 min each at room temperature. Cells were incubated with the appropriate secondary antibody: goat-anti-mouse IgG Alexa 488 or goat-anti-rabbit IgG Alexa 643 (Invitrogen) at 1:1,000 dilution in 5% BSA/1 × PBS (pH 7.4) for 30 min at room temperature in the dark. Finally, preparations were washed thrice for 5 min each at room temperature using 1 × PBS. Subsequently, cells were counterstained with DAPI (4,6-diamidino-2-phenylindole; Sigma-Aldrich), washed thrice with 1 × PBS for 10 min at room temperature, embedded in Fluoromount G (Southern Biotech) and kept at 4 °C until further analysis. The analysis was performed using an Axio Observer A1 microscope (Carl Zeiss MicroImaging, Goettingen, Germany).

### RC-seq library preparation, sequencing and analysis

Genomic DNA was isolated from 1 × 10^6^ cells from each hESC and hiPSC line and their respective parental cells using DNAzol Genomic DNA Isolation Reagent (MRC Inc, Cincinnati, OH, USA) according to the manufacturer’s instructions. RC-seq and subsequent computational analyses were performed as described using the hg19 reference genome sequence^33^. A total of 665,008,770 2 × 150mer reads were generated from 24 libraries. A complete list of annotated *de novo* insertions supported by at least two unique amplicons separated by ≥5 nt (the minimum threshold for reporting) is provided in Supplementary Data 1. To assess the RC-seq false negative rate, we randomly sampled each library in increments of 1% (10 samplings per percentile) and determined how many germline insertions were detected at the sampled depth by ≥2 unique reads (Supplementary Fig. 11). To approximately assess the rate of L1 mobilization in hiPSCs, we again randomly sampled each RC-seq library to determine the probability of detecting each *de novo* L1 insertion with ≥2 unique reads at a given sampling depth, normalized to the corresponding false negative rate identified above and then determined the cumulative sum of this distribution for frequencies of 5–100%, leading to an estimate of ∼1 *de novo* L1 insertion per hiPSC. We did not consider *de novo* L1 insertions carried by fewer than 5% of hiPSCs in this estimate as none of the validated examples were routinely identified at that sampling depth. We also did not analyse the L1 mobilization rate in hESCs or the *Alu* or SVA rate in hiPSCs or hESCs due to the small number of confirmed true positive examples.

A permutation test showing enrichment for validated *de novo* L1 insertions at the 5′ end of genes was performed by random sampling of genomic coordinates, with respect to RefSeq annotations. 1 × 10^6^ permutations were performed and in 6,000 instances the average position was less than the 20th percentile of gene length, indicating *P*<0.006.

### PCR validation of *de novo* insertions

Seventeen *de novo* insertions (eight L1, seven *Alu* and two SVA) detected by RC-seq were first assayed with PCR using a standard empty site/filled site genotyping assay. Primers were positioned on either side of the insertion site so that the predicted PCR product of the empty site covered <300 bp. Additional retrotransposon specific primers were designed and paired with the existing insertion site-specific primers if required. In cases where an insertion was detected by RC-seq at one terminus only, PCR and capillary sequencing were applied to the remaining end to resolve integration site structure. PCR reactions contained 0.125 μl Crimson Taq (New England Biolabs), 5 × PCR-buffer, 10pMol of each Primer, 10 mM dNTPs and 10–20 ng genomic template DNA in a total volume of 25 μl. The following cycling conditions were used: 95 °C for 2 min, then 35 cycles of 95 °C for 30 s, 58 °C for 30 s, 68 °C for 40 s, followed by a single extension step at 68 °C for 5 min. Optimization in some cases required adjusted annealing temperatures and cycle number. PCR products of the correct size (Fig. 3a) that were obtained with the retrotransposon primer in combination with the genomic primer were TA-cloned and sequenced. The same method was applied to both the 5′ and the 3′ ends of all *de novo* insertions to fully characterize each, apart from SVA-2. To PCR amplify the 5' junction of the SVA-2 insertion from genomic DNA, we designed three SVA_E_-specific primers and three oligonucleotides binding 50–300 bp upstream of the SVA-2 integration site. To facilitate the detection of a potentially 5′-truncated SVA, the SVA-specific primers were placed within the sequenced 123 bp of the SVA-2 3′ end (Supplementary Fig. 9), at the junctions of the SVA_E_-specific *Alu*-like and VNTR region, and the (CCCTCT)*n* repeat and *Alu*-like region, respectively. Combinatorial use of these genome-SVA primer pairs did either not result in a PCR product or generated non-specific products. For a complete list of used primers see Supplementary Table 3. Eleven *de novo* insertions (seven L1, three *Alu* and one SVA) were confirmed by PCR as *de novo*. Six additional insertions were determined as germline insertions, already present in the parental cell line or an early hESC passage. Control genotyping PCR of the single-copy gene GAPDH in genomic DNA preparations of parental and hiPSC lines used for RC-seq and PCR validations of *de novo* insertions is presented in Supplementary Fig. 15. PCR amplification was performed using primers GAPDH-α (5′-CAAAGCTTGTGCCCAGACTGTG3′) and GAPDH-β (5′-GAGAGCTGGGGAATGGGACT3′) which bind in exon 8 (chr12:6646561-6646580) and intron 7 (chr12:6647005-6647026), respectively, resulting in a 466-bp DNA fragment. Cycling conditions were identical to those described above.

### Quantification of L1-dn13 and L1-dn14 copy numbers by qPCR

To determine relative copy numbers of *de novo* insertions L1-dn13 and L1-dn14 within the hiPS-SB4 culture, we applied real-time qPCR using TaqMan fluorogenic probes. To this end, genomic DNA was isolated using 1 ml DNAzol Genomic DNA Isolation Reagent (MRC Inc, Cincinnati, OH, USA) from 1 × 10^6^ cells, according to the manufacturer’s instructions. A total of 100 ng of genomic DNA was used for quantitative real-time PCR (qPCR). Primer and probe combinations specific to the genomic 5′ junctions of the *de novo* insertions L1-dn13 and L1-dn14 (Fig. 3b) were used to quantify the copy number of the respective insertion in hiPSC cultivars. Each probe was labelled with flourochrom6-carboxyfluorescein and a non-fluorescent quencher. For normalization the single-copy gene *RPP25* (Ribonuclease P/MRP 25kDa subunit; FAM/non-fluorescent quencher, primer limited, HS00706565_S1; Applied Biosystems) was used. Cycling conditions were: 95 °C for 15 min (one cycle), 95 °C for 15 s and 60 °C for 1 min (40 cycles). For analysis of real-time and end point fluorescence, the software SDS version 2.3 as well as RQ manager 1.2 (Applied Biosystems) were used.

### Isolation of hiPS-SB4 single-cell subclones

To isolate single cell subclones from the hiPS-SB4 culture by limiting dilution, hiPS-SB4 cells of passage 64 representing a mixed population of cells with and without the L1-dn13 *de novo* retrotransposition event, were magnetically separated from feeder cells by applying a Feeder Removal Kit (Miltenyi Biotech GmbH, Bergisch Gladbach, Germany) according to the manufacturer’s instructions. hiPSCs were counted and seeded on feeder-coated 96-well plates (Catalogue no.: 167008, Thermo Fisher/Nunc, Roskilde, Denmark) at a cell density of one cell per well or 0.3 cells per well. hiPSCs were grown for 24 h in the presence of 10 μM ROCK inhibitor (Y-27632, Sigma-Aldrich). Subsequently, cells were cultivated until they formed a single colony per well. Single colonies were transferred to feeder-coated 12-well plates (Thermo Fisher/Nunc) and further expanded. To isolate genomic DNA from each clone, cells were harvested after collagenase IV treatment, centrifuged, washed and pelleted again. Genomic DNA was isolated as described in the previous paragraph. Genotyping PCR conditions applied to screen for the presence of the L1-dn13 insertion and to demonstrate its presence/absence (Fig. 5d) are identical to those described above for insertion PCR validation. Primers used to demonstrate presence/absence of L1-dn13 are provided in Supplementary Data 1 and Supplementary Table 3. PCR products were visualized on a 1.5% agarose gel after ethidium bromide staining.

### L1 retrotransposition reporter assays

*De novo* full-length L1 insertions were amplified from genomic DNA using an Expand Long Template PCR system (Roche) and primers located 50 bp upstream/downstream the insertion site (available upon request). For each PCR we used: 0.3 μl Expand Long Template Taq (Roche), 1 × buffer#1, 400 μM dNTPs, 1 μM each Primer and 300 ng genomic DNA in 50 μl per tube. Cycling conditions were: 95 °C for 5 min, then 30 cycles of 95 °C for 1 min, 56 °C for 30 s, 68 °C for 6 min, followed by a single extension step at 68 °C for 10 min. To avoid the generation of mutations that may lead to retrotransposition defective elements, we conducted at least four independent PCRs per L1. PCR products were resolved on 0.9% agarose gels, and fragments of the expected length of ∼6 kb representing potential full-length L1 elements were excised and purified using a Qiaquick kit (Qiagen) and cloned in the Topo-XL plasmid (Invitrogen). Each of the cloned PCR products carrying full-length L1 elements L1-dn4, L1-dn6 and L1-dn14 were sequenced (Supplementary Fig. 10). To evaluate retrotransposition competence of the L1-dn6 *de novo* insertion, two independent genomic PCR amplicons, L1-dn6-5.4 and L1-dn6-2.2, were sequenced and inserted into the pJJ101/L1.3 backbone after the deletion of its L1.3 sequence by *Not* I/*Bst*Z17I restriction^9^,^72^. pJJ101/L1.3 contains the active full-length L1.3 element tagged with an *mblastI* retrotransposition indicator cassette^72^ cloned in vector pCEP4 (Invitrogen). In total, we generated five JJ101-derived plasmids containing an L1-dn6 element amplified from genomic DNA by PCR. For retrotransposition assays, these L1 reporter plasmids were purified using a Qiagen Midiprep system (Qiagen) and only highly supercoiled preparations were used in the following assays.

Retrotransposition assays in HeLa cells were conducted as described previously^9^,^46^,^52^,^72^. HeLa cells were purchased from ATCC. Cytogenetic authentication of HeLa cells was performed by spectral karyotyping (SKY)-FISH. HeLa cells used in this study were tested for mycoplasma contamination monthly. Briefly, HeLa cells were cultured using DMEM-high glucose (4.5 g l^−1^) supplemented with L-glutamine, Penicillin/Streptomycin, and 10% fetal bovine serum (all reagents from GIBCO-Invitrogen) and passaged using Trypsin 0.05% (GIBCO-Invitrogen). 10^4^ HeLa cells per well were plated in triplicate using six-well tissue culture plates. After 18 h, cells were transfected with 1 μg per well of plasmid using 3 μl of Fugene6 (Promega) following manufacturer’s instructions. Next day, medium was replaced and cells cultured for five additional days. Six days after transfection, Blasticidin-S (Invitrogen) was added to a final concentration of 10 μg ml^−1^ and cells were cultured for seven days in the presence of the antibiotic. Next, plates were fixed and stained with crystal violet, and foci counted manually.

### Statistical analyses of relative L1 RNA levels

The statistical evaluation of relative L1 mRNA levels determined by qRT–PCR was performed by ANOVA, using Bonferroni correction for multiple comparisons with the same control group. Reduction in full-length transcript levels in the embryoid body time kinetics experiment was evaluated by means of Linear Regression for data from day 0 to day 8 (*R*^2^=0.79). Analyses were performed with SAS/STAT software, version 9.2 SAS system for Windows.

## Additional Information

**Accession codes:** The RC-seq FASTQ data have been deposited in the Sequence Read Archive (SRA) under accession code PRJEB3191.

**How to cite this article:** Klawitter, S. *et al.* Reprogramming triggers endogenous L1 and *Alu* retrotransposition in human induced pluripotent stem cells. *Nat. Commun.* 7:10286 doi: 10.1038/ncomms10286 (2016).

## Supplementary Material

Supplementary Information Supplementary Figures 1-15, Supplementary Tables 1-3, Supplementary Methods and Supplementary References

Supplementary Data 1 Endogenous retrotransposon insertions in analyzed hiPSC and hESC lines identified by RC-seq

## Figures and Tables

**Table 1:** Analysed pluripotent stem cell lines and their characteristics.

**Stem cell line**	**Parental cells**		**Reprogramming factors**	**Factor delivery by**	**Reference**	**Passages (p) assayed by RC-seq**
	**Name**	**Description**				
hiPS-SB4	HFF-1	Foreskin fibroblasts (male)	OCT-4, SOX2, KLF4 and c-MYC	Sleeping Beauty transposon	26	43, 53
hiPS-SB5			OCT-4, SOX2, KLF4, c-MYC and LIN28			40, 59
hiPS-CRL1502	CRL1502	Dermal fibroblasts (female)	OCT-4, SOX2, NANOG, LIN28, KLF4 and c-MYC	oriP/EBNA1-based pCEP4 episomal vector	25	15, 40
hiPS-CRL2429	CRL2429	Dermal fibroblasts (male)				11, 40
hiPS-FB	FB	Dermal fibroblasts (female)	OCT-4, SOX2, KLF4 and c-MYC	Lentiviral vector	68	7, 23
hFF-iPS4	HFF-1	Foreskin fibroblasts (male)	OCT-4, SOX2, NANOG and LIN28		Unpublished	58
hCBiPS1hCBiPS2	hCBEC	Cord blood-derived endothelial cells (male)	OCT-4, SOX2, NANOG and LIN28		27	3023
HES-3	hESC lines		NA	NA	73	92, 102
H9					74	30, 60
HESG					Unpublished	10, 23

## References

[b1] Yamanaka S. Induced pluripotent stem cells: past, present, and future. Cell Stem Cell 10, 678–684 (2012) . 22704507 10.1016/j.stem.2012.05.005

[b2] Gore A. *et al.* Somatic coding mutations in human induced pluripotent stem cells. Nature 471, 63–67 (2011) . 21368825 10.1038/nature09805 PMC3074107

[b3] Hussein S. M. *et al.* Copy number variation and selection during reprogramming to pluripotency. Nature 471, 58–62 (2011) . 21368824 10.1038/nature09871

[b4] Laurent L. C. *et al.* Dynamic changes in the copy number of pluripotency and cell proliferation genes in human ESCs and iPSCs during reprogramming and time in culture. Cell Stem Cell 8, 106–118 (2011) . 21211785 10.1016/j.stem.2010.12.003 PMC3043464

[b5] Lister R. *et al.* Hotspots of aberrant epigenomic reprogramming in human induced pluripotent stem cells. Nature 471, 68–73 (2011) . 21289626 10.1038/nature09798 PMC3100360

[b6] Mayshar Y. *et al.* Identification and classification of chromosomal aberrations in human induced pluripotent stem cells. Cell Stem Cell 7, 521–531 (2010) . 20887957 10.1016/j.stem.2010.07.017

[b7] Ben-David U. & Benvenisty N. The tumorigenicity of human embryonic and induced pluripotent stem cells. Nat. Rev. Cancer 11, 268–277 (2011) . 21390058 10.1038/nrc3034

[b8] Levin H. L. & Moran J. V. Dynamic interactions between transposable elements and their hosts. Nat. Rev. Genet. 12, 615–627 (2011) . 21850042 10.1038/nrg3030 PMC3192332

[b9] Beck C. R. *et al.* LINE-1 retrotransposition activity in human genomes. Cell 141, 1159–1170 (2010) . 20602998 10.1016/j.cell.2010.05.021 PMC3013285

[b10] Mills R. E., Bennett E. A., Iskow R. C. & Devine S. E. Which transposable elements are active in the human genome? Trends Genet. 23, 183–191 (2007) . 17331616 10.1016/j.tig.2007.02.006

[b11] Brouha B. *et al.* Hot L1s account for the bulk of retrotransposition in the human population. Proc. Natl Acad. Sci. USA 100, 5280–5285 (2003) . 12682288 10.1073/pnas.0831042100 PMC154336

[b12] Ewing A. D. & Kazazian H. H. Jr. Whole-genome resequencing allows detection of many rare LINE-1 insertion alleles in humans. Genome Res. 21, 985–990 (2011) . 20980553 10.1101/gr.114777.110 PMC3106331

[b13] Garcia-Perez J. L. *et al.* LINE-1 retrotransposition in human embryonic stem cells. Hum. Mol. Genet. 16, 1569–1577 (2007) . 17468180 10.1093/hmg/ddm105

[b14] Kano H. *et al.* L1 retrotransposition occurs mainly in embryogenesis and creates somatic mosaicism. Genes Dev. 23, 1303–1312 (2009) . 19487571 10.1101/gad.1803909 PMC2701581

[b15] Han J. S., Szak S. T. & Boeke J. D. Transcriptional disruption by the L1 retrotransposon and implications for mammalian transcriptomes. Nature 429, 268–274 (2004) . 15152245 10.1038/nature02536

[b16] Hancks D. C. & Kazazian H. H. Jr. Active human retrotransposons: variation and disease. Curr. Opin. Genet. Dev. 22, 191–203 (2012) . 22406018 10.1016/j.gde.2012.02.006 PMC3376660

[b17] Beck C. R., Garcia-Perez J. L., Badge R. M. & Moran J. V. LINE-1 elements in structural variation and disease. Annu. Rev. Genomics Hum. Genet. 12, 187–215 (2011) . 21801021 10.1146/annurev-genom-082509-141802 PMC4124830

[b18] Bourc'his D. & Bestor T. H. Meiotic catastrophe and retrotransposon reactivation in male germ cells lacking Dnmt3L. Nature 431, 96–99 (2004) . 15318244 10.1038/nature02886

[b19] Maherali N. *et al.* Directly reprogrammed fibroblasts show global epigenetic remodeling and widespread tissue contribution. Cell Stem Cell 1, 55–70 (2007) . 18371336 10.1016/j.stem.2007.05.014

[b20] Wissing S. *et al.* Reprogramming somatic cells into iPS cells activates LINE-1 retroelement mobility. Hum. Mol. Genet. 21, 208–218 (2012) . 21989055 10.1093/hmg/ddr455 PMC3235014

[b21] Friedli M. *et al.* Loss of transcriptional control over endogenous retroelements during reprogramming to pluripotency. Genome Res. 24, 1251–1259 (2014) . 24879558 10.1101/gr.172809.114 PMC4120079

[b22] Wissing S., Montano M., Garcia-Perez J. L., Moran J. V. & Greene W. C. Endogenous APOBEC3B restricts LINE-1 retrotransposition in transformed cells and human embryonic stem cells. J. Biol.Chem. 286, 36427–36437 (2011) . 21878639 10.1074/jbc.M111.251058 PMC3196128

[b23] Cheng L. *et al.* Low incidence of DNA sequence variation in human induced pluripotent stem cells generated by nonintegrating plasmid expression. Cell Stem Cell 10, 337–344 (2012) . 22385660 10.1016/j.stem.2012.01.005 PMC3298448

[b24] Quinlan A. R. *et al.* Genome sequencing of mouse induced pluripotent stem cells reveals retroelement stability and infrequent DNA rearrangement during reprogramming. Cell Stem Cell 9, 366–373 (2011) . 21982236 10.1016/j.stem.2011.07.018 PMC3975295

[b25] Briggs J. A. *et al.* Integration-free induced pluripotent stem cells model genetic and neural developmental features of down syndrome etiology. Stem Cells 31, 467–478 (2013) . 23225669 10.1002/stem.1297

[b26] Grabundzija I. *et al.* Sleeping Beauty transposon-based system for cellular reprogramming and targeted gene insertion in induced pluripotent stem cells. Nucleic Acids Res. 41, 1829–1847 (2013) . 23275558 10.1093/nar/gks1305 PMC3561994

[b27] Haase A. *et al.* Generation of induced pluripotent stem cells from human cord blood. Cell Stem Cell 5, 434–441 (2009) . 19796623 10.1016/j.stem.2009.08.021

[b28] Abyzov A. *et al.* Somatic copy number mosaicism in human skin revealed by induced pluripotent stem cells. Nature 492, 438–442 (2012) . 23160490 10.1038/nature11629 PMC3532053

[b29] Goodier J. L., Zhang L., Vetter M. R. & Kazazian H. H. Jr. LINE-1 ORF1 protein localizes in stress granules with other RNA-binding proteins, including components of RNA interference RNA-induced silencing complex. Mol. Cell. Biol. 27, 6469–6483 (2007) . 17562864 10.1128/MCB.00332-07 PMC2099616

[b30] Doucet A. J. *et al.* Characterization of LINE-1 ribonucleoprotein particles. PLoS Genet. 6, e1001150 (2010) . 20949108 10.1371/journal.pgen.1001150 PMC2951350

[b31] Rodic N. *et al.* Long interspersed element-1 protein expression is a hallmark of many human cancers. Am. J. Pathol. 184, 1280–1286 (2014) . 24607009 10.1016/j.ajpath.2014.01.007 PMC4005969

[b32] Moldovan J. B. & Moran J. V. The zinc-finger antiviral protein ZAP inhibits LINE and Alu retrotransposition. PLoS Genet. 11, e1005121 (2015) . 25951186 10.1371/journal.pgen.1005121 PMC4423928

[b33] Shukla R. *et al.* Endogenous retrotransposition activates oncogenic pathways in hepatocellular carcinoma. Cell 153, 101–111 (2013) . 23540693 10.1016/j.cell.2013.02.032 PMC3898742

[b34] Baillie J. K. *et al.* Somatic retrotransposition alters the genetic landscape of the human brain. Nature 479, 534–537 (2011) . 22037309 10.1038/nature10531 PMC3224101

[b35] Ewing A. D. & Kazazian H. H. Jr. High-throughput sequencing reveals extensive variation in human-specific L1 content in individual human genomes. Genome Res. 20, 1262–1270 (2010) . 20488934 10.1101/gr.106419.110 PMC2928504

[b36] Iskow R. C. *et al.* Natural mutagenesis of human genomes by endogenous retrotransposons. Cell 141, 1253–1261 (2010) . 20603005 10.1016/j.cell.2010.05.020 PMC2943760

[b37] Wang J. *et al.* dbRIP: a highly integrated database of retrotransposon insertion polymorphisms in humans. Hum. Mutat. 27, 323–329 (2006) . 16511833 10.1002/humu.20307 PMC1855216

[b38] Mir A. A., Philippe C. & Cristofari G. euL1db: the European database of L1HS retrotransposon insertions in humans. Nucleic Acids Res. 43, D43–D47 (2015) . 25352549 10.1093/nar/gku1043 PMC4383891

[b39] Wang H. *et al.* SVA elements: a hominid-specific retroposon family. J. Mol. Biol. 354, 994–1007 (2005) . 16288912 10.1016/j.jmb.2005.09.085

[b40] Symer D. E. *et al.* Human l1 retrotransposition is associated with genetic instability *in vivo*. Cell 110, 327–338 (2002) . 12176320 10.1016/s0092-8674(02)00839-5

[b41] Raiz J. *et al.* The non-autonomous retrotransposon SVA is trans-mobilized by the human LINE-1 protein machinery. Nucleic Acids Res. 40, 1666–1683 (2012) . 22053090 10.1093/nar/gkr863 PMC3287187

[b42] Gilbert N., Lutz S., Morrish T. A. & Moran J. V. Multiple fates of L1 retrotransposition intermediates in cultured human cells. Mol. Cell. Biol. 25, 7780–7795 (2005) . 16107723 10.1128/MCB.25.17.7780-7795.2005 PMC1190285

[b43] Luan D. D., Korman M. H., Jakubczak J. L. & Eickbush T. H. Reverse transcription of R2Bm RNA is primed by a nick at the chromosomal target site: a mechanism for non-LTR retrotransposition. Cell 72, 595–605 (1993) . 7679954 10.1016/0092-8674(93)90078-5

[b44] Cost G. J., Feng Q., Jacquier A. & Boeke J. D. Human L1 element target-primed reverse transcription *in vitro*. EMBO J. 21, 5899–5910 (2002) . 12411507 10.1093/emboj/cdf592 PMC131089

[b45] Jurka J. Sequence patterns indicate an enzymatic involvement in integration of mammalian retroposons. Proc. Natl Acad. Sci USA 94, 1872–1877 (1997) . 9050872 10.1073/pnas.94.5.1872 PMC20010

[b46] Morrish T. A. *et al.* DNA repair mediated by endonuclease-independent LINE-1 retrotransposition. Nat. Genet. 31, 159–165 (2002) . 12006980 10.1038/ng898

[b47] Zingler N. *et al.* Analysis of 5' junctions of human LINE-1 and Alu retrotransposons suggests an alternative model for 5'-end attachment requiring microhomology-mediated end-joining. Genome Res. 15, 780–789 (2005) . 15930490 10.1101/gr.3421505 PMC1142468

[b48] Ostertag E. M. & Kazazian H. H. Jr. Twin priming: a proposed mechanism for the creation of inversions in L1 retrotransposition. Genome Res. 11, 2059–2065 (2001) . 11731496 10.1101/gr.205701 PMC311219

[b49] Grimaldi G., Skowronski J. & Singer M. F. Defining the beginning and end of KpnI family segments. EMBO J. 3, 1753–1759 (1984) . 6090124 10.1002/j.1460-2075.1984.tb02042.x PMC557592

[b50] Lee E. *et al.* Landscape of somatic retrotransposition in human cancers. Science 337, 967–971 (2012) . 22745252 10.1126/science.1222077 PMC3656569

[b51] Solyom S. *et al.* Extensive somatic L1 retrotransposition in colorectal tumors. Genome Res. 22, 2328–2338 (2012) . 22968929 10.1101/gr.145235.112 PMC3514663

[b52] Moran J. V. *et al.* High frequency retrotransposition in cultured mammalian cells. Cell 87, 917–927 (1996) . 8945518 10.1016/s0092-8674(00)81998-4

[b53] Sassaman D. M. *et al.* Many human L1 elements are capable of retrotransposition. Nat. Genet. 16, 37–43 (1997) . 9140393 10.1038/ng0597-37

[b54] Cost G. J., Golding A., Schlissel M. S. & Boeke J. D. Target DNA chromatinization modulates nicking by L1 endonuclease. Nucleic Acids Res. 29, 573–577 (2001) . 11139628 10.1093/nar/29.2.573 PMC29664

[b55] Thurman R. E. *et al.* The accessible chromatin landscape of the human genome. Nature 489, 75–82 (2012) . 22955617 10.1038/nature11232 PMC3721348

[b56] Kaer K. & Speek M. Retroelements in human disease. Gene 518, 231–241 (2013) . 23333607 10.1016/j.gene.2013.01.008

[b57] Guenther M. G. *et al.* Chromatin structure and gene expression programs of human embryonic and induced pluripotent stem cells. Cell Stem Cell 7, 249–257 (2010) . 20682450 10.1016/j.stem.2010.06.015 PMC3010384

[b58] Nott A., Meislin S. H. & Moore M. J. A quantitative analysis of intron effects on mammalian gene expression. RNA 9, 607–617 (2003) . 12702819 10.1261/rna.5250403 PMC1370426

[b59] Schmittgen T. D. *et al.* Quantitative reverse transcription-polymerase chain reaction to study mRNA decay: comparison of endpoint and real-time methods. Anal. Biochem. 285, 194–204 (2000) . 11017702 10.1006/abio.2000.4753

[b60] Coufal N. G. *et al.* L1 retrotransposition in human neural progenitor cells. Nature 460, 1127–1131 (2009) . 19657334 10.1038/nature08248 PMC2909034

[b61] Arokium H. *et al.* Deep sequencing reveals low incidence of endogenous LINE-1 retrotransposition in human induced pluripotent stem cells. PLoS ONE 9, e108682 (2014) . 25289675 10.1371/journal.pone.0108682 PMC4188539

[b62] Upton K. R. *et al.* Ubiquitous L1 mosaicism in hippocampal neurons. Cell 161, 228–239 (2015) . 25860606 10.1016/j.cell.2015.03.026 PMC4398972

[b63] van den Hurk J. A. *et al.* L1 retrotransposition can occur early in human embryonic development. Hum. Mol. Genet. 16, 1587–1592 (2007) . 17483097 10.1093/hmg/ddm108

[b64] Marchetto M. C. *et al.* Differential L1 regulation in pluripotent stem cells of humans and apes. Nature 503, 525–529 (2013) . 24153179 10.1038/nature12686 PMC4064720

[b65] Ostertag E. M. & Kazazian H. H. Jr. Biology of mammalian L1 retrotransposons. Annu. Rev. Genet. 35, 501–538 (2001) . 11700292 10.1146/annurev.genet.35.102401.091032

[b66] Coufal N. G. *et al.* Ataxia telangiectasia mutated (ATM) modulates long interspersed element-1 (L1) retrotransposition in human neural stem cells. Proc. Natl Acad. Sci. USA 108, 20382–20387 (2011) . 22159035 10.1073/pnas.1100273108 PMC3251057

[b67] Kapranov P. *et al.* RNA maps reveal new RNA classes and a possible function for pervasive transcription. Science 316, 1484–1488 (2007) . 17510325 10.1126/science.1138341

[b68] Becherel O. J. *et al.* A new model to study neurodegeneration in ataxia oculomotor apraxia type 2. Hum. Mol. Genet. 24, 5759–5774 (2015) . 26231220 10.1093/hmg/ddv296 PMC4581605

[b69] Andrews P. W., Bronson D. L., Benham F., Strickland S. & Knowles B. B. A comparative study of eight cell lines derived from human testicular teratocarcinoma. Int. J. Cancer 26, 269–280 (1980) . 6169654 10.1002/ijc.2910260304

[b70] Kimberland M. L. *et al.* Full-length human L1 insertions retain the capacity for high frequency retrotransposition in cultured cells. Hum. Mol. Genet. 8, 1557–1560 (1999) . 10401005 10.1093/hmg/8.8.1557

[b71] Watanabe N. & Mitchison T. J. Single-molecule speckle analysis of actin filament turnover in lamellipodia. Science 295, 1083–1086 (2002) . 11834838 10.1126/science.1067470

[b72] Kopera H. C., Moldovan J. B., Morrish T. A., Garcia-Perez J. L. & Moran J. V. Similarities between long interspersed element-1 (LINE-1) reverse transcriptase and telomerase. Proc. Natl Acad. Sci. USA 108, 20345–20350 (2011) . 21940498 10.1073/pnas.1100275108 PMC3251045

[b73] Richards M., Fong C. Y., Chan W. K., Wong P. C. & Bongso A. Human feeders support prolonged undifferentiated growth of human inner cell masses and embryonic stem cells. Nat. Biotechnol. 20, 933–936 (2002) . 12161760 10.1038/nbt726

[b74] Thomson J. A. *et al.* Embryonic stem cell lines derived from human blastocysts. Science 282, 1145–1147 (1998) . 9804556 10.1126/science.282.5391.1145

