# Author Correction: Reprogramming triggers endogenous L1 and *Alu* retrotransposition in human induced pluripotent stem cells

**DOI:** 10.1038/s41467-018-07917-0

**Published:** 2018-12-19

**Authors:** Sabine Klawitter, Nina V. Fuchs, Kyle R. Upton, Martin Muñoz-Lopez, Ruchi Shukla, Jichang Wang, Marta Garcia-Cañadas, Cesar Lopez-Ruiz, Daniel J. Gerhardt, Attila Sebe, Ivana Grabundzija, Sylvia Merkert, Patricia Gerdes, J. Andres Pulgarin, Anja Bock, Ulrike Held, Anett Witthuhn, Alexandra Haase, Balázs Sarkadi, Johannes Löwer, Ernst J. Wolvetang, Ulrich Martin, Zoltán Ivics, Zsuzsanna Izsvák, Jose L. Garcia-Perez, Geoffrey J. Faulkner, Gerald G. Schumann

**Affiliations:** 1 Division of Medical Biotechnology, Paul-Ehrlich-Institute, D-63225 Langen, Germany; 2 Max-Delbrück-Center for Molecular Medicine, D-13125 Berlin, Germany; 3 Mater Research Institute, University of Queensland, TRI Building, Woolloongabba, Brisbane, Queensland 4102 Australia; 4 Department of Human DNA Variability, Pfizer/University of Granada and Andalusian Regional Government Center for Genomics and Oncology (GENYO), PTS Granada, 18016 Granada, Spain; 5 MRC Human Genetics Unit, Institute of Genetics and Molecular Medicine, University of Edinburgh, Crewe Road, Edinburgh, EH4 2XU UK; 6 Leibniz Research Laboratories for Biotechnology and Artificial Organs (LEBAO), Department of Cardiac, Thoracic, Transplantation, and Vascular Surgery; REBIRTH, Cluster of Excellence, Hannover Medical School, D-30625 Hannover, Germany; 7 Department of Biophysics and Radiation Biology, Semmelweis University, H-1094 Budapest, Hungary; 8 Australian Institute for Bioengineering and Nanotechnology, The University of Queensland, St Lucia, Queensland 4072 Australia; 9 Queensland Brain Institute, University of Queensland, Brisbane, Queensland 4072 Australia; 10 Present Address: Division of Inborn Metabolic Diseases, University Children’s Hospital, D-69120 Heidelberg, Germany

Correction to: *Nature Communications*; 10.1038/ncomms10286; published online 8 January 2016

This Article contains an error in the author affiliations. The correct affiliation for author Ruchi Shukla is ‘MRC Human Genetics Unit, Institute of Genetics and Molecular Medicine, University of Edinburgh, Crewe Road, Edinburgh, EH4 2XU, UK’, and is not ‘Mater Research Institute – University of Queensland, TRI Building, Woolloongabba QLD 4102, Australia’.

