# Supplementary material for: Reprogramming triggers endogenous L1 and Alu retrotransposition in human induced pluripotent stem cells
Source: Nat Commun. 2016 Jan 8;7:10286. doi: 10.1038/ncomms10286 (PMC4729875; doi:10.1038/ncomms10286)
Supplement: Supplementary Information — Supplementary Figures 1-15, Supplementary Tables 1-3, Supplementary Methods and Supplementary References [file ncomms10286-s1.pdf]

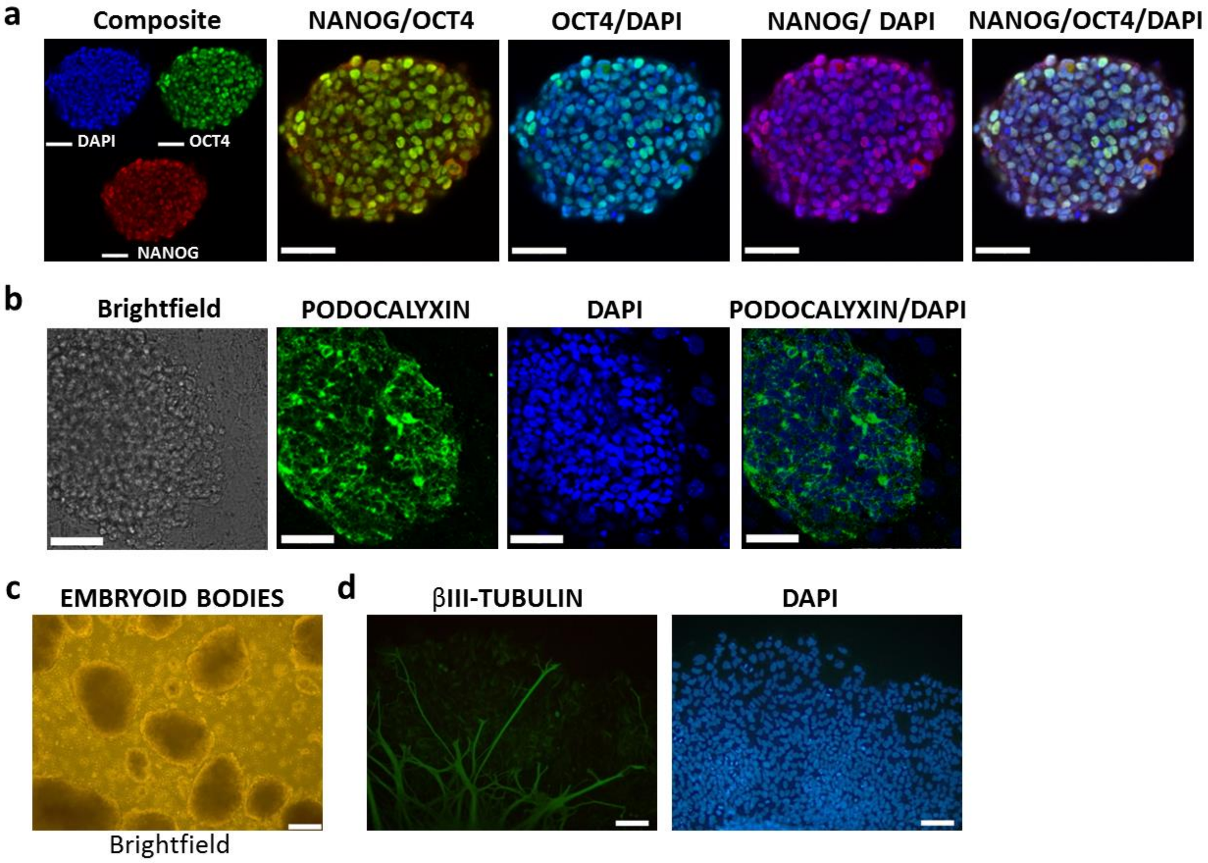

Supplementary Figure 1: Characterization of the hiPSC line hiPS-SB4. Undifferentiated hiPS-SB4 cells stain positively for the typical pluripotency markers **(a)** OCT4, NANOG, and **(b)** PODOCALYXIN. **(c)** hiPS-SB4 cells were kept in suspension on polyHEMA-coated plates in embryoid body (EB)-medium to allow formation of cystic EBs. **(d)** After 6 days, EBs were placed onto gelatin-coated plates, and the spontaneously differentiating, attached cells were positive for the ectodermal marker βIII-TUBULIN. Scale bars indicate 50 μm **(a, b)** and 200 μm **(c, d)**, respectively.

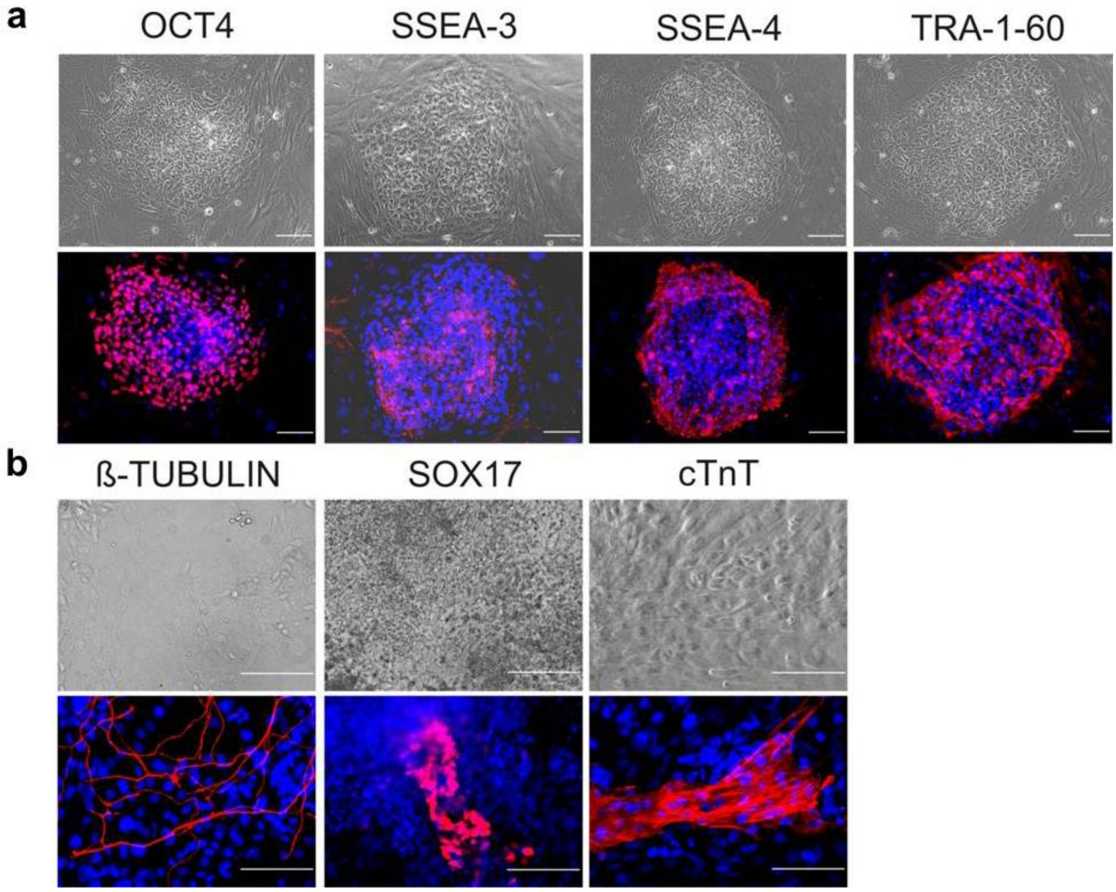

Supplementary Figure 2: Characterization of the hFF-iPS4 cell line. **(a)** Undifferentiated hFF-iPS4 cells (passage 8) express typical markers of pluripotency like OCT4, SSEA-3, SSEA-4 and TRA-1-60. **(b)** Cells are differentiated via an embryoid body (EB)-based protocol. On day 23 of differentiation, cultures are stained for markers of all three germ layers:βIII-tubulin for ectoderm, SOX17 for endoderm and cardiac TroponinT (cTnT) for mesoderm. Nuclei are stained with DAPI. Scale bars represent 100 µm;

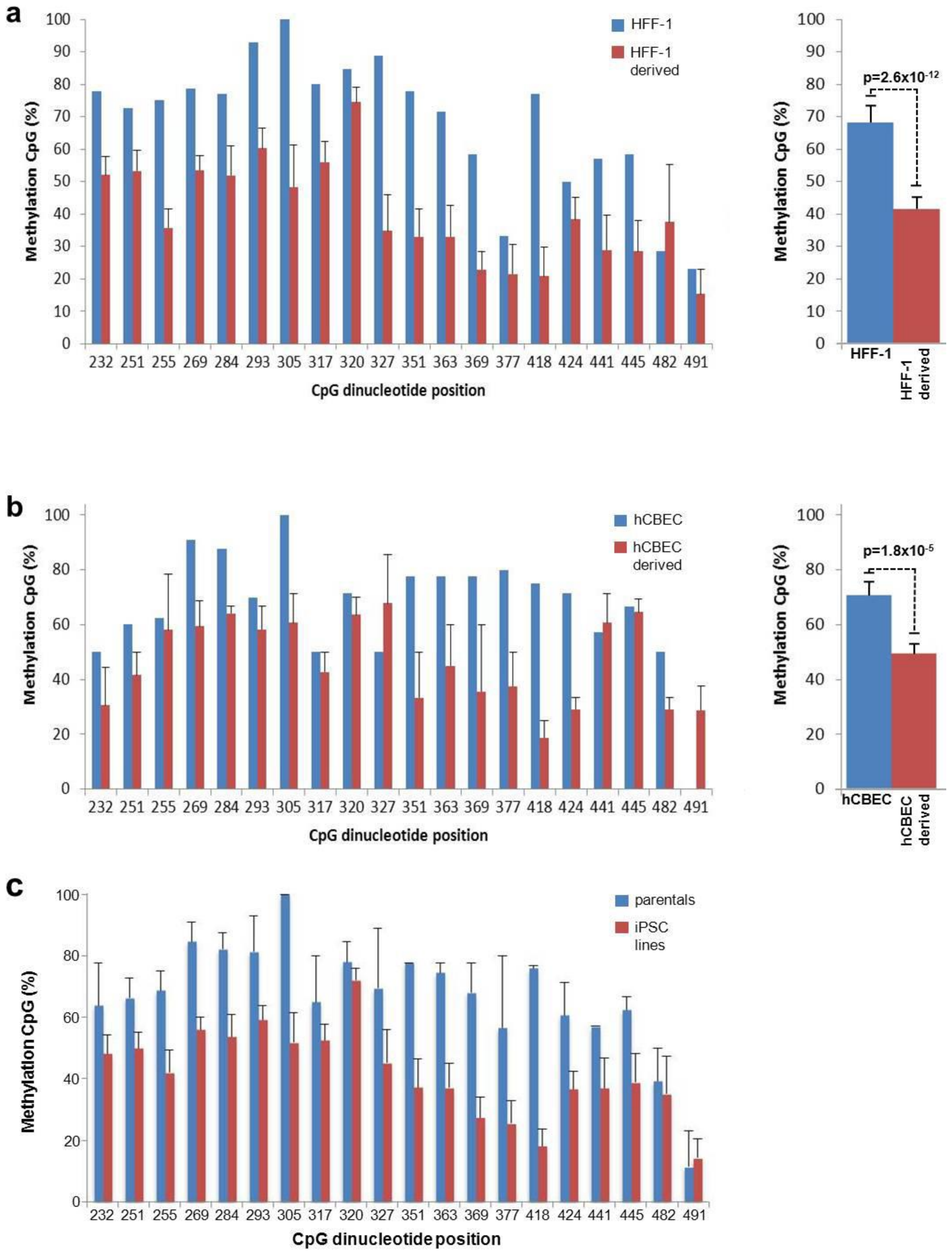

Supplementary Figure S3

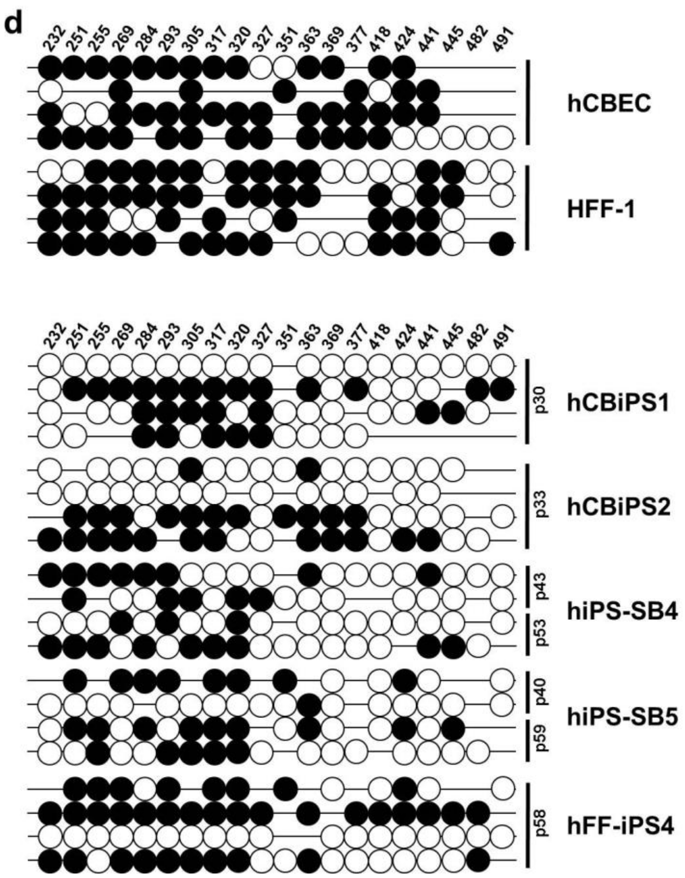

Supplementary Figure 3: Reprogramming into iPSC lines results in global demethylation of L1 promoter sequences. Comparison of L1 promoter methylation levels in parental HFF-1 **(a)** and hCBEC **(b)** cells with their respective derived iPSC lines. Each bar represents the arithmetic mean of methylation frequencies of the parental HFF-1 (**a**, left panel, blue bars, n=18 from one sample) and hCBEC cells (**b**, left panel, blue bars, n=11 from one sample), respectively, and their derived iPSC lines (**a**, left panel, red bars, n=66 from five independent samples; left panel, red bars, n=29 from two independent samples) at defined CpG positions. Error bars indicate s.e.m for each CpG position. Right panels represent the overall percentage methylation status of 20 CpG residues in lines hiPS-SB4, hiPS-SB5, and hFF-iPS4 (red bar, 41.35%) relative to their parental cell lines HFF-1 (blue bar, 67.8%) **(a),** and in lines hCBiPS1 and hCBiPS2 (red bar, 49.11%) relative to their parental cell line hCBEC (blue, 70,37%) **(b);**). p-values calculated using the $\chi 2$ test. **(c)** Overall methylation status of each CpG position in the genomic L1-Ta promoter sequences of the five hiPSC lines and their parental cells. The graph was generated by pooling the data presented in Supplementary Figure 3a and 3b for each CpG position. Each bar represents the arithmetic mean of methylation frequencies of both parental cells/cell lines HFF-1 and hCBEC (blue bars, n=371, from two independent samples) and the derived iPSC lines (red bars, n=1116, from seven independent samples). Error bars indicate s.e.m. n, number of independent bisulfite sequencing reads. **(d)** Bisulfite sequencing of L1 promoter regions in hCBEC and HFF-1 cells and their derived iPSC lines (hCBiPS1, hCBiPS2, hiPS-SB4, hiPS-SB5, hFF-iPS4). Genomic DNA samples were isolated at different passages (p30 to p59). The individual methylation status of sequences showing the highest similarity to the retrotransposition-competent L1.3 reference element is presented. Each line depicts an independent clone. Open and closed circles indicate unmethylated and methylated CpG dinucleotides, respectively. Mutated CpG sites are indicated by the absence of any circle. Numbers refer to the position of each CpG site in L1.3.

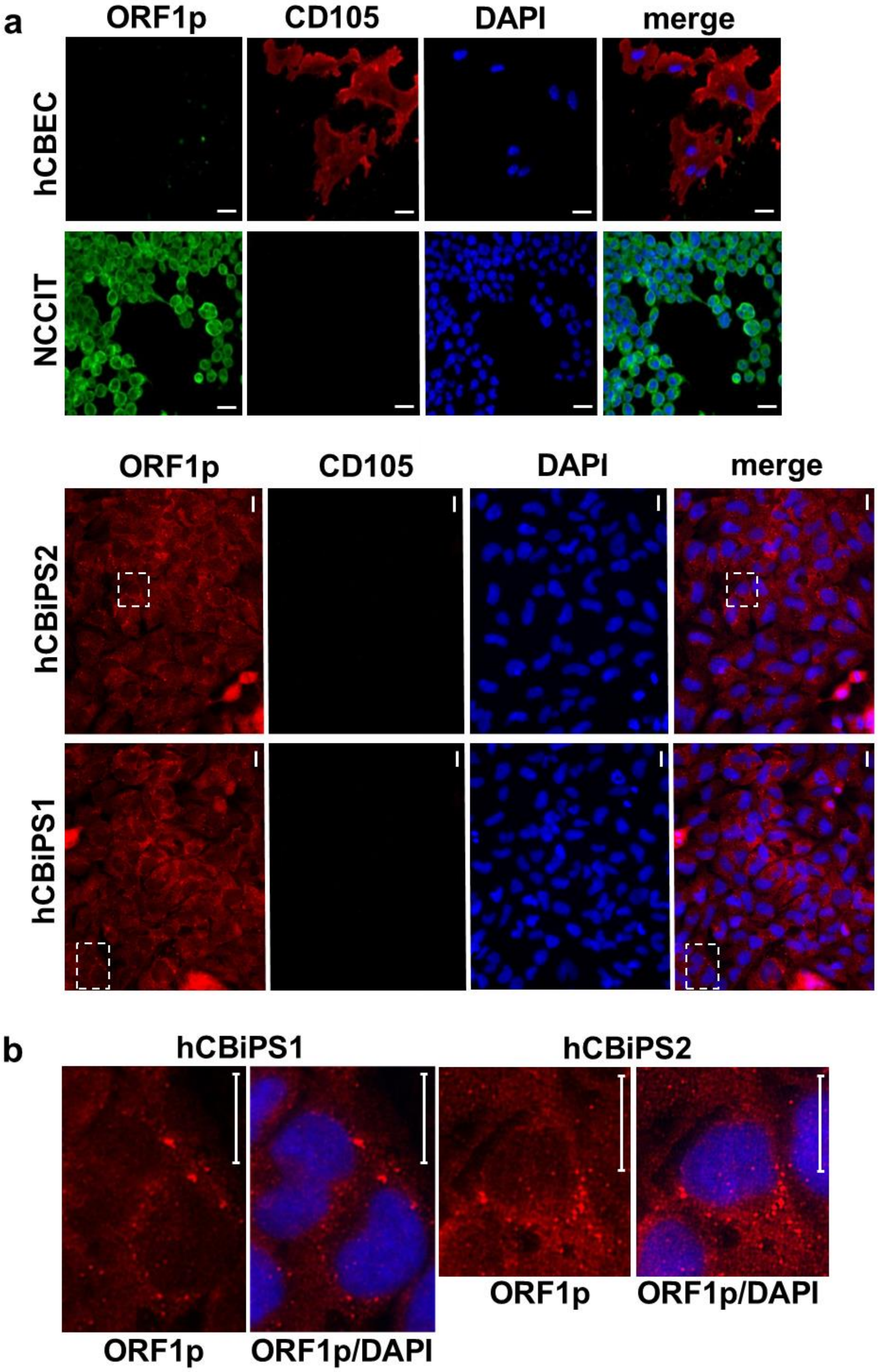

Supplementary Figure 4: Immunostaining of hiPSC lines hCBiPS1 and hCBiPS2 and their parental cells to monitor induction of endogenous L1 ORF1p expression in hCBEC-derived iPSCs. (a) Cytoplasmic ORF1p staining indicates activation of endogenous L1 expression after reprogramming of human cord blood-derived endothelial cells into iPSC lines hCBiPS1 and hCBiPS2. Cell lines were assayed at passages number 34 and 27, respectively. The pluripotent embryonal carcinoma cell line NCCIT served as positive control for L1 ORF1p expression. CD105, marker protein expressed on vascular endothelial cells, but absent from pluripotent stem cells. Nuclei are stained with DAPI (blue). Scale bars represent 20 μm. Stippled white boxes indicate the enlarged areas presented in **(b)** showing cytoplasmic localization of endogenous L1 ORF1p accumulated in foci.

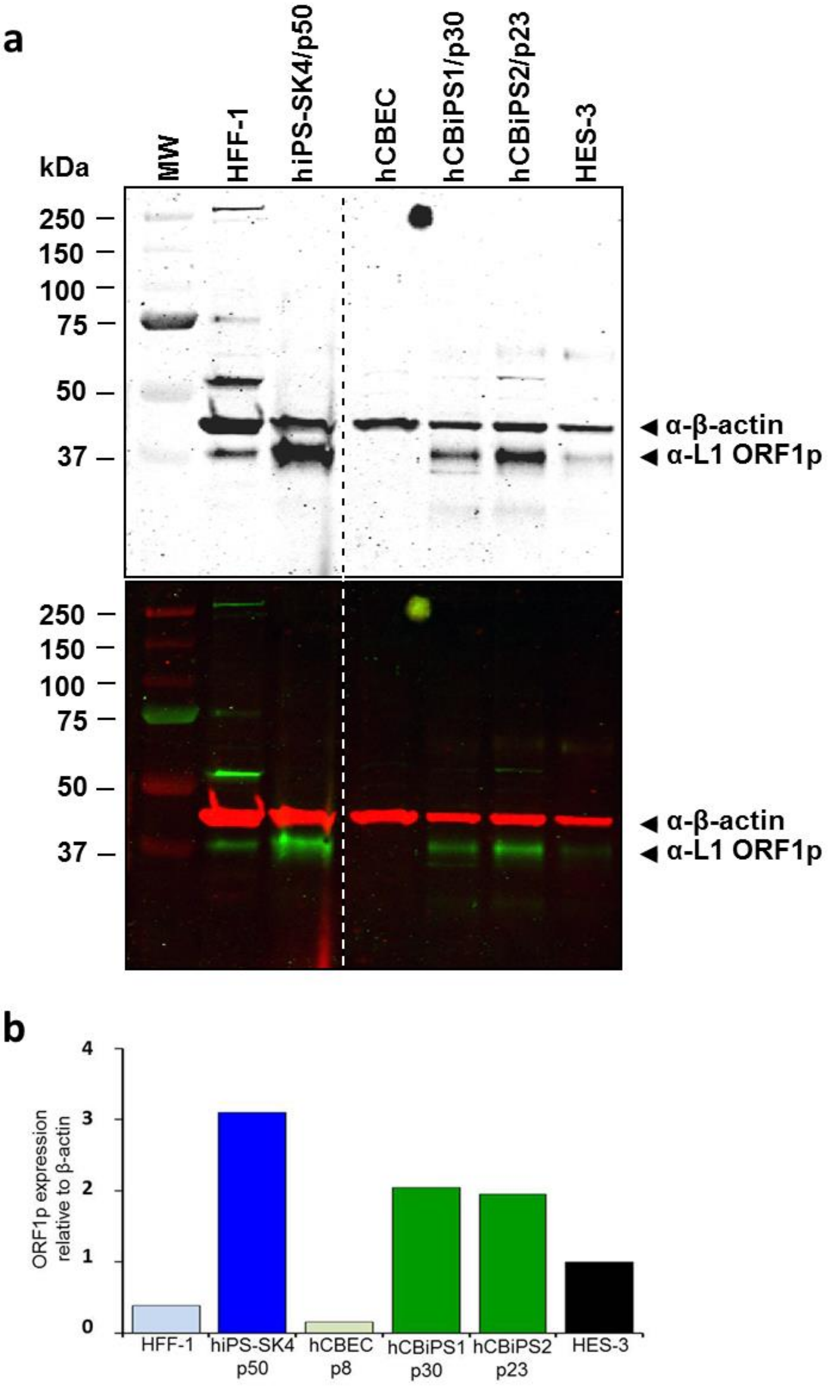

Supplementary Figure 5: Quantification of the relative increase of endogenous L1 ORF1 protein levels triggered by reprogramming of HFF-1 and hCBEC cells into iPSC lines. (a) Immunoblot analysis of whole cell lysates from the parental HFF-1 and hCBEC cells and their respective descendant hiPSC lines hFF-iPS4, hCBiPS1, hCBiPS2 was performed using polyclonal α-ORF1p and α-β-actin antibodies (upper two panels). 20 μg of each cell lysate were loaded per lane. Cells were harvested at indicated passages (p) after reprogramming. Stippled line indicates that two lanes of this gel were excised here because the loaded cell lysates were degraded. The image of the multiplexed detection of ORF1p and β-actin using two infrared fluorescence fluorophores is shown as bottom panel (see Methods). ORF1p expression levels were determined by quantifying the ORF1p/β-actin expression rate (see Methods). **(b)** Relative ORF1p expression in the different cell lines is presented as bar diagram. Endogenous L1 ORF1p levels are increased by ~8 and ~12-fold in late passage hiPSCs relative to their parental HFF-1 and hCBEC cells, respectively. Endogenous L1 protein levels in HFF-1 and hCBEC-derived hiPSCs are also elevated by 2 and 3-fold, respectively, relative to embryonic HES-3 stem cells. The ratio of ORF1p/ β-actin signal in HES-3 cells was set as 1.

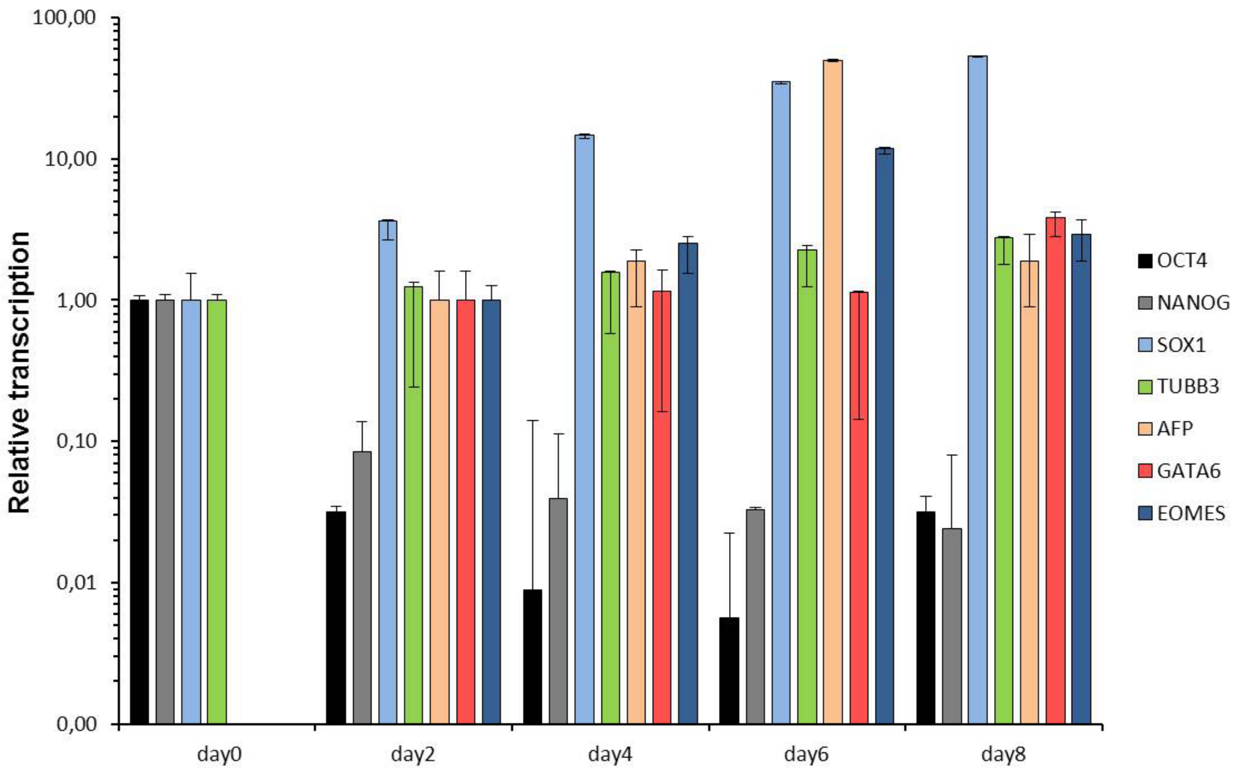

Supplementary Figure 6: Real-Time qRT PCR analysis to follow multi-lineage differentiation of hiPS-SB5.1 cells into EBs. Expression of endogenous (NANOG) and transgenic (OCT4) pluripotency-related factors as well as endoderm (α-fetoprotein [AFP], GATA-binding factor 6 [GATA6]), mesoderm (eomesodermin [EOMES]), and ectoderm-specific (SOX1, βIII-tubulin [TUBB3]) marker proteins was quantified before (p10/day 0) and after 2, 4, 6 and 8 days of differentiation into EBs. mRNA levels of the factors OCT4, NANOG, SOX1 and TUBB3 in iPSCs at passage 10 (day 0) are set as 1. mRNA levels of the factors AFP, GATA6 and EOMES in iPSCs that were differentiated for 2 days (day 2) were set as 1 because transcript levels of these factors were not determined at day 0. Data were normalized to GAPDH transcript levels. Each bar represents the arithmetic mean ± s.e.m of data obtained from experiments that were performed as biological triplicates (n=3).

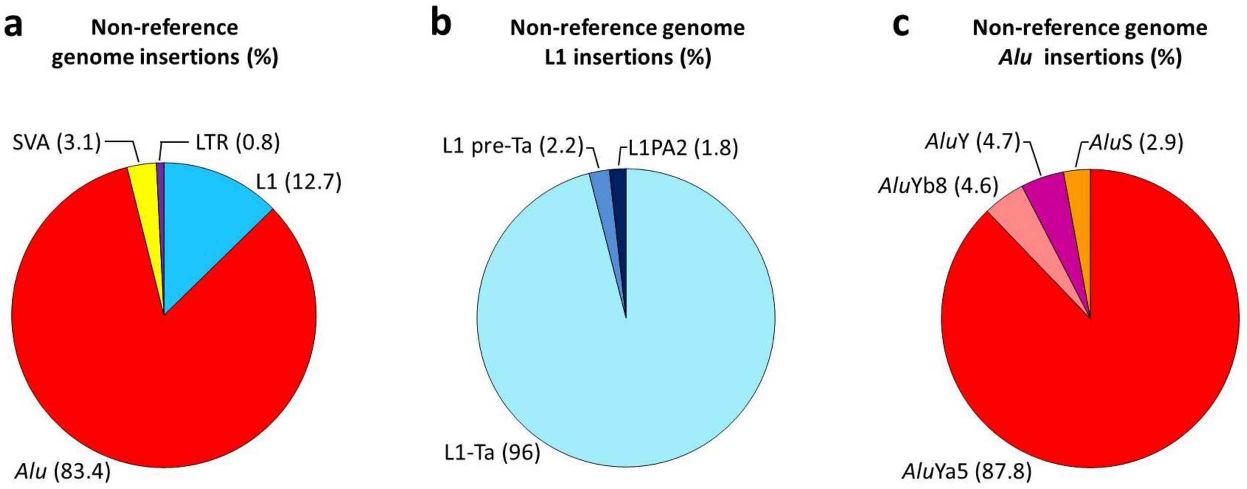

Supplementary Figure 7: Relative frequencies of non-reference genome retrotransposon insertions of a) different retrotransposon families as detected by RC-seq, and of non-reference genome insertions of L1 (b) and *Alu* (c) subfamilies. Total number of non-reference inserions detected by RC-seq, and defined as 100% in (a), n=40,608.

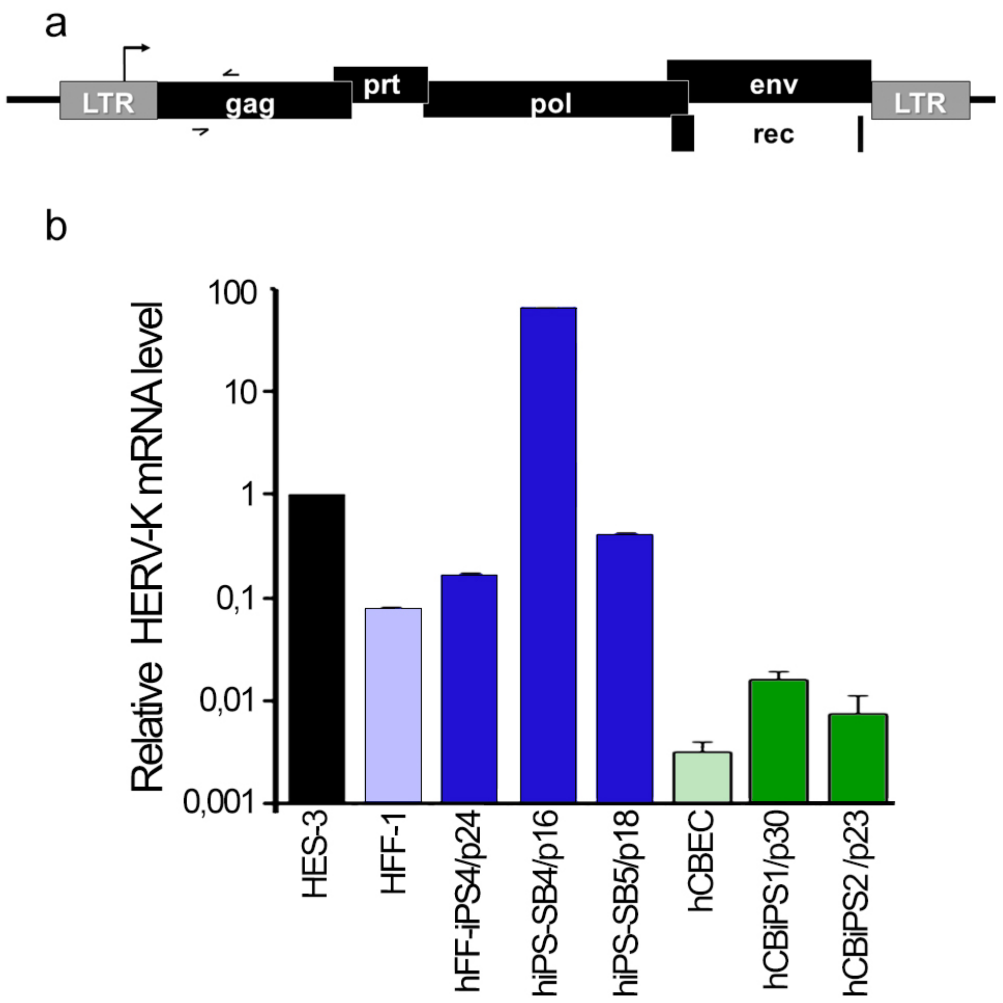

Supplementary Figure 8: HERV-K/HML-2 mRNA levels are upregulated in pluripotent stem cells - Reprogramming causes activation of HERV-K/HML-2 transcription in iPSCs. **(a)** Structure of the HERV-K/HML-2 endogenous retrovirus. Primers (arrows) used for qRT-PCR anneal within the *gag*-region of cDNAs generated from HERV-K/HML-2 full length mRNA (Supplementary Table 1) and amplify a 167-bp product. LTR, long terminal repeat; gag, group-specific antigen; prt, protease, pol, polymerase; env, envelope; rec, HERV-K-specific accessory protein. **(b)** HERV-K/HML-2 transcription is upregulated at early passages after reprogramming into iPSC lines. Real-Time qRT-PCR analyses were performed with cDNAs generated from cytoplasmic RNA preparations isolated from parental cells (HFF-1 and hCBEC) and from earlier passages of their derived iPSC lines (Fig. 1b/left panel; Table 1). Reprogramming lead to a 2 to 827- fold increase of relative HERV-K expression levels in HFF-1-derived iPSC lines hFF-iPS4, hiPS-SB4 and hiPS-SB5, and a 2 to 5-fold increase in hCBEC-derived iPSC lines hCBiPS1 and hCBiPS2. The endogenous HERV-K/HML-2 transcript level in HES-3 cells was set as 1. Each bar depicts the arithmetic mean ±s.d. from biological triplicates (n=3).

a) *De novo* retrotransposition events in hiPSCs

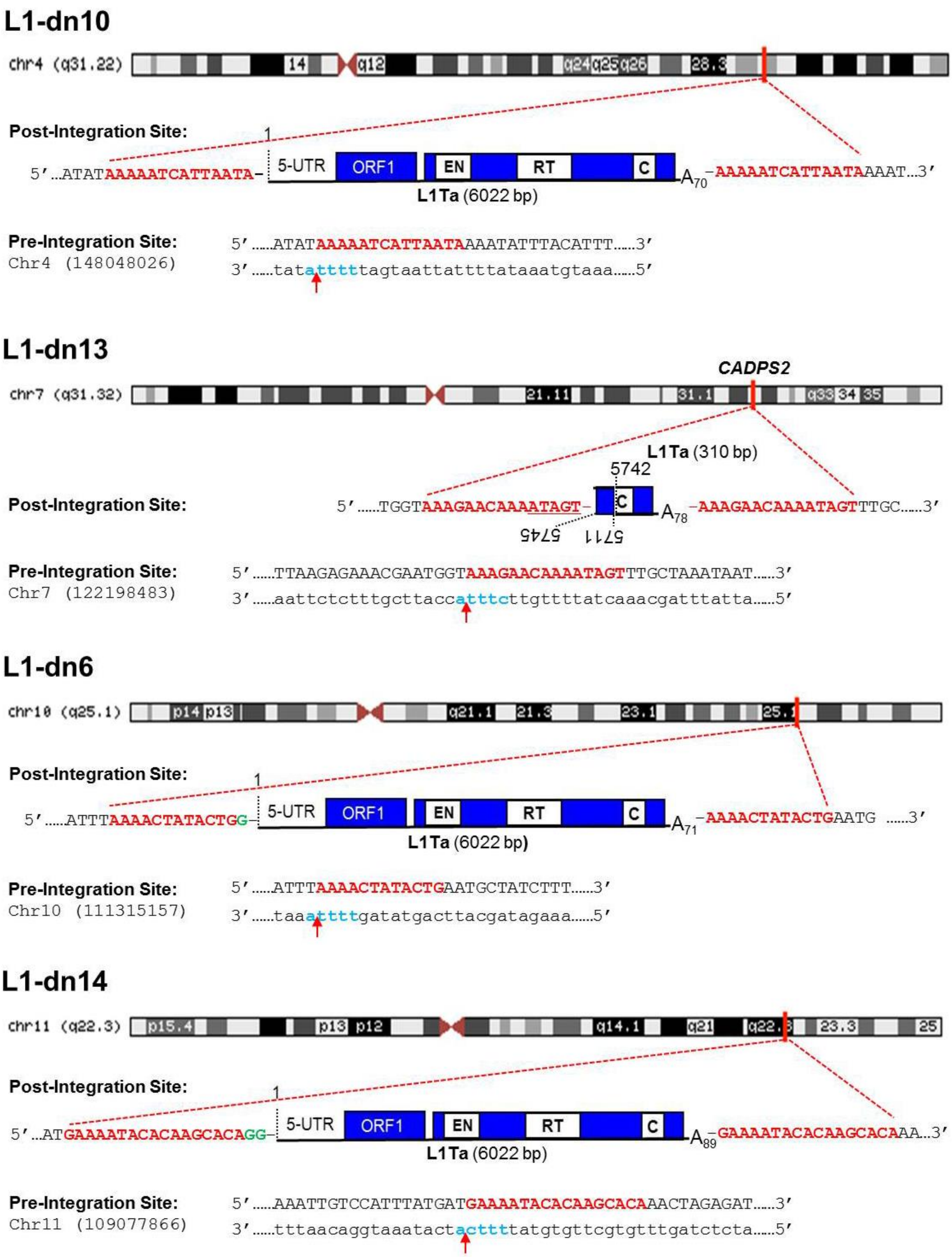

Supplementary Figure S9

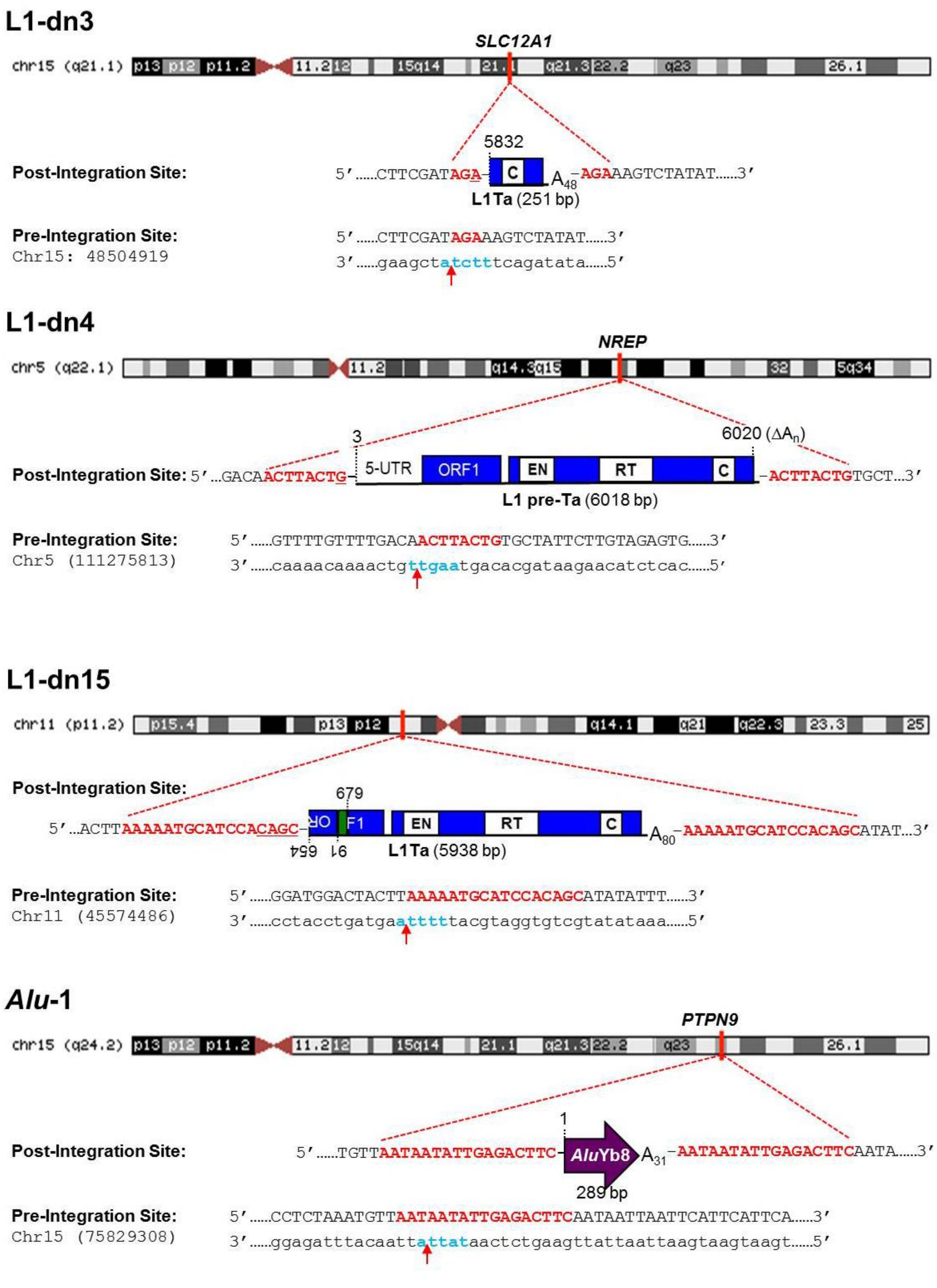

Supplementary Figure S9 continued

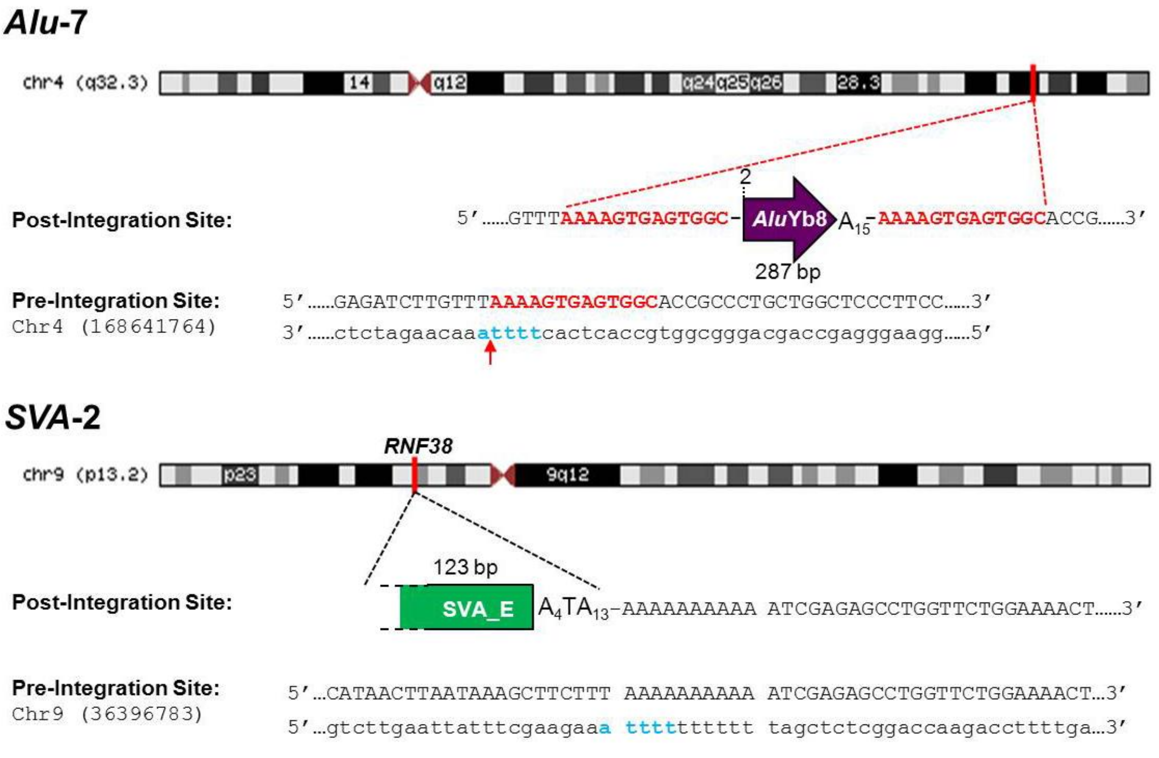

b) *De novo* retrotransposition event in the hESC line H9

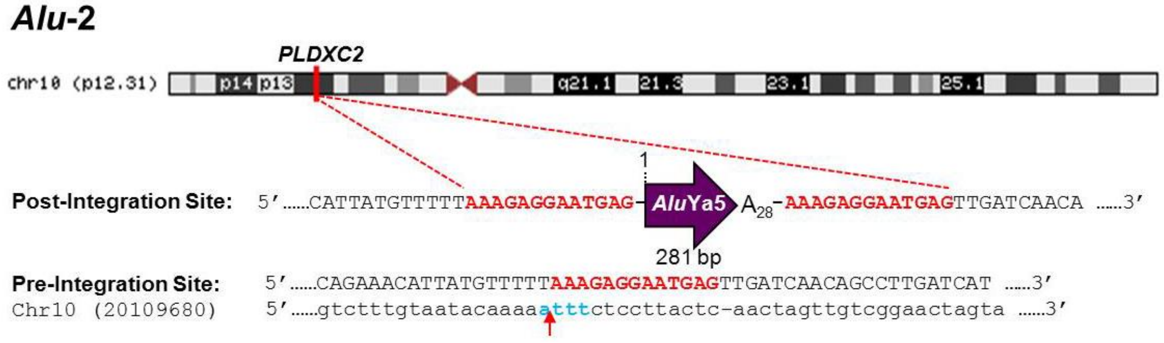

Supplementary Figure 9: Structures of endogenous L1, *Alu* and SVA *de novo* retrotransposition events identified in human iPSC (a) and ESC (b) lines. Chromosomal localization, and pre- and post-integration sites are presented. *De novo* insertions were identified in hiPSC lines hiPS-SB4, hiPS-SB5, hFF-iPS4 and hiPS-CRL1502 respectively. Nucleotide positions at 5' and 3' ends of L1 insertions refer to the L1 reference sequence L1.3[1]. L1 subfamily type and insertion lengths are indicated: L1-dn10, L1-dn6, L1-dn14 and L1-dn15 are intergenic full-length L1Ta insertions; L1-dn13, 5' truncated and 5' inverted L1Ta insertion into intron 7 of the *CADPS2* gene; L1-dn3, 5' truncated L1Ta intronic insertion into intron 2 of the *SLC12A1 gene*; L1-dn4, full-length L1 pre-Ta insertion into intron 2 of the *NREP* gene incorporating a 2bp 3' truncation; L1-dn15, 5' truncated and inverted intergenic insertion; The inverted region at the 5' end of insertion L1-dn15 ranges from pos. 91-654 of the L1 reference sequence and is followed by a 25-bp sequence of unknown origin which is attached to pos. 679 of a 5'-truncated L1 Ta element. *Alu*-1, 289-bp *Alu*Yb8 insertion into intron 1 of the *PTPN9* gene; *Alu*-7, intergenic 287-bp *Alu*Yb8 insertion; SVA-2, 3' junction of the intronic SVA_E insertion into the *RNF38* gene; *Alu*-2, *Alu*Ya5 insertion into intron 2 of the *PLDXC2* gene; L1 EN target sequences on the bottom strands are indicated in blue. L1 EN nicking site is denoted by a red arrow. Extra deoxyguanylates at the 5' ends of *de novo* insertions are indicated in green. Nucleotides representing patches of microcomplementarity are underlined; Red lettering, TSD sequences;

L1-dn4

```
   1 GCGAAGGGGA TGTGTCTACT GCAGTTCTCT TCACTGTGGA CCTGGAACAT GGCAGACAGA CAGACACTTT GATCTCCTTG CCAGTACGAC TTCATGTCTG
 101 CTGTAGTTTG GAGTAGCAGA GAGAAGGCAA CAACTTCCTG GTTGTCTTCT GAACTGTACT TTGATAATCA GTAGGTAGAA AAGAGAGGGA ACAGTATTTC
 201 AAGAACTGTT TTTGTTTTAT CTTTTATCCC TATCCTTAAA CTATATTTTG TTTTTGGTAT TTCGTAGTCT TTTTTCAGGT AAGGTATATT AGGCATCTTT
                                                                                            TSD
                                                                                         ~~~~~~~~~
 301 ATTTATCACA AAAGGCTATC ACAGTGTTTC CCTTTATGTA AGGAGTAAAT TGAAGGAATA GTTTTGTTTT GACAACTTAC TGGGAGGAGC CAAGATGGCC
 401 GAATAGGAAC AGCTCCGGTC TACAGCTCCC AGCGTGAGCG ACGCAGAAGA CGGTGATTTC TGCATTTCCA TCTGAGGTAC CGGGTTCATC TCACTAGGGA
 501 GTGCCAGACA GTGGGCGCAG GCCAGTGTGT GTGCGCACCG TGCGCGAGCC GAAGCAGGGC GAGGCATTGC CTCACCTGGG AAGCGCAAGG GGTCAGGGAG
 601 TTCCCTTTCT GAGTCAAAGA AAGGGGTGAC GGTCGCACCT GGAAAATCGG GTCACTCCCA CCCGAATATT GCGCTTTTCA GACCGGCTTA AGAAACGGCG
 701 CACCACGAGA CTATATCCCA CACCTGGCTC GGAGGGTCCT ACGCCCACGG AATCTCGCTG ATTGCTAGCA CAGCAGTCTG AGATCAAACT GCAAGGCGGC
 801 AACGAGGCTG GGGGAGGGGC GCCCGCCATT GCCCAGGCTT GCTTAGGTAA ACAAAGCAGC CGGGAAGCTC GAACTGGGTG GAGCCCACCA CAGCTCAAGG
 901 AGGCCTGCCT GCCTCTGTAG GCTCCACCTC TGGGGGCAGG GCACAGACAA ACAAAAAGAC AGCAGTAACC TCTGCAGACT TAAGTGTCCC TGTCTGACAG
1001 CTTTGAAGAG AGCAGTGGTT CTCCCAGCAC ACAGCTGGAG ATCTGAGAAC TGGCAGACTG CCTCCTCAAG TGGGTCCCTG ACCCCTGACC CCCGAGCAGC
1101 CTAACTGGGA GGCACCCCCC ACCAGGGGCA CACTGACACC TCACACGGCA GGGTATTCCA GCAGACCTGC AGCTGAGGGT TCTGTCTGTT AGAAGGAAAA

                                                                                                         ORF1
                                                                                                  ~~~~~~~~~~~~~~~~~
                                                                                                   M  G   K  K  Q  N ·
1201 CTAACAACCA GAAAGGACAT CTACACCGAA AACCCATCTG TACATCACCA TCATCAAAGA CCAAAAGTAG ATAAAACCAC AAAGATGGGG AAAAAACAGA

                                                    ORF1
     ·  R  K  T   G  N  S   K  T  Q  S   V  S  P   P  P  K   E  R  S  S   S  P  A   T  E  Q   S  W  M  E   N  D  F ·
1301 ACAGAAAAAC TGGAAACTCT AAAACGCAGA GCGTCTCTCC TCCTCCAAAG GAACGCAGTT CCTCACCAGC AACAGAACAA AGCTGGATGG AGAATGATTT

                                                    ORF1
     · D  E  L   R  E  E  G   F  R  R   S  N  Y   S  E  L  R   E  D  I   Q  T  K   G  K  E  V   E  N  F   E  K  N
1401 TGACGAGCTG AGAGAAGAAG GCTTCAGACG ATCAAATTAC TCTGAGCTAC GGGAGGACAT TCAAACCAAA GGCAAAGAAG TTGAAAACTT TGAAAAAAAT

                                                    ORF1
      L  E  E  C   I  T  R   I  T  N   T  E  K  C   L  K  E   L  M  E   L  K  T  K   A  R  E   L  R  E   E  C  R  S ·
1501 TTAGAAGAAT GTATAACTAG AATAACCAAT ACAGAGAAGT GCTTAAAGGA GCTGATGGAG CTGAAAACCA AGGCTCGAGA ACTACGTGAA GAATGCAGAA

                                                    ORF1
     ·  L  R  S   R  C  D   Q  L  E  E   R  V  S   A  M  E   D  E  M  N   E  M  K   R  E  G   K  F  R  E   K  R  I ·
1601 GCCTCAGGAG CCGATGCGAT CAACTGGAAG AAAGGGTATC AGCAATGGAA GATGAAATGA ATGAAATGAA GCGAGAAGGG AAGTTTAGAG AAAAAAGAAT

                                                    ORF1
     · K  R  N   E  Q  S  L   Q  E  I   W  D  Y   V  K  R  P   N  L  R   L  I  G   V  P  E  S   D  V  E   N  G  T
1701 AAAAAGAAAT GAGCAAAGCC TCCAAGAAAT ATGGGACTAT GTGAAAAGAC CAAATCTACG TCTGATTGGT GTACCTGAAA GTGATGTGGA GAATGGAACC

                                                    ORF1
      K  L  E  N   T  L  Q   D  I  I   Q  E  N  F   P  N  L   A  R  Q   A  N  V  Q   I  Q  E   I  Q  R   T  P  Q  R ·
1801 AAGTTGGAAA ACACTCTGCA GGATATTATC CAGGAGAACT TCCCCAATCT AGCAAGGCAG GCCAACGTTC AGATTCAGGA AATACAGAGA ACGCCACAAA

                                                    ORF1
     ·  Y  S  S   R  R  A   T  P  R  H   I  I  V   R  F  T   K  V  E  M   K  E  K   M  L  R   A  A  R  E   K  G  R ·
1901 GATACTCCTC GAGAAGAGCA ACTCCAAGAC ACATAATTGT GAGATTCACC AAAGTTGAAA TGAAGGAAAA AATGTTAAGG GCAGCCAGAG AGAAAGGTCG

                                                    ORF1
     · V  T  L   K  G  K  P   I  R  L   T  A  D   L  S  A  E   T  L  Q   A  R  R   E  W  G  P   I  F  N   I  L  K
2001 GGTTACCCTC AAAGGAAAGC CCATCAGACT AACAGCGGAT CTCTCGGCAG AAACCCTACA AGCCAGAAGA GAGTGGGGGC CAATATTCAA CATTCTTAAA

                                                    ORF1
      E  K  N  F   Q  P  R   I  S  Y   P  A  K  L   S  F  I   S  E  G   E  I  K  Y   F  I  D   K  Q  M   L  R  D  F ·
2101 GAAAAGAATT TTCAACCCAG AATTTCATAT CCAGCCAAAC TAAGCTTCAT AAGTGAAGGA GAAATAAAAT ACTTTATAGA CAAGCAAATG CTGAGAGATT

                                                   ORF1
     ·  V  T  T   R  P  A   L  K  E  L   L  K  E   V  L  N   M  E  R  N   N  R  Y   Q  P  L   Q  N  H  A   K  M
2201 TTGTCACCAC CAGGCCTGCC CTAAAAGAGC TCCTGAAGGA AGTGCTAAAC ATGGAAAGGA ACAACCGGTA CCAGCCGCTG CAAAATCATG CCAAAATGTA

                                                                                                ORF2
                                                                               M  T   G  S  N  S   H  I  T   I  L  T
2301 AAGACCATCG AGACTAGGAA GAAACTGCAT CAACTAATGA GCAAAATCAC CAGCTAACAT CATAATGACA GGATCAAATT CACACATAAC AATATTAACT

                                                    ORF2
      L  N  I  N   G  L  N   S  A  I   K  R  H  R   L  A  S   W  I  K   S  Q  D  P   S  V  C   C  I  Q   E  T  H  L ·
2401 TTAAATATAA ATGGACTAAA TTCTGCAATT AAAAGACACA GACTGGCAAG TTGGATAAAG AGTCAAGACC CATCAGTGTG CTGTATTCAG GAAACCCATC

                                                    ORF2
     ·  T  C  R   D  T  H   R  L  K  I   T  G  W   R  K  I   Y  Q  A  N   G  K  Q   K  K  A   G  V  A  I   L  V  S ·
2501 TCACGTGCAG AGACACACAT AGGCTCAAAA TAACAGGATG GAGGAAGATC TACCAAGCAA ATGGAAAACA AAAAAAGGCA GGGGTTGCAA TCCTAGTCTC

                                                    ORF2
     · D  K  T   D  F  K  P   T  K  I   K  R  D   K  E  G  H   Y  I  M   V  K  G   S  I  Q  Q   E  E  L   T  I  L
2601 TGATAAAACA GACTTTAAAC CAACAAAGAT CAAAAGAGAC AAAGAAGGCC ATTACATAAT GGTAAAGGGA TCAATTCAAC AAGAGGAGCT AACTATCCTA

                                                    ORF2
      N  I  Y  A   P  N  T   G  A  P   R  F  I  K   Q  V  L   S  D  L   Q  R  D  L   D  S  H   T  L  I   M  G  D  F ·
2701 AATATTTATG CACCCAATAC AGGAGCACCC AGATTCATAA AGCAAGTCCT CAGTGACCTA CAAAGAGACT TAGACTCCCA CACATTAATA ATGGGAGACT

                                                    ORF2
     ·  N  T  P   L  S  T   L  D  R  S   T  R  Q   K  V  N   K  D  T  Q   E  L  N   S  A  L   H  Q  A  D   L  I  D ·
```

```
2801 TTAACACCCC ACTGTCAACA TTAGACAGAT CAACGAGACA GAAAGTCAAC AAGGATACCC AGGAATTGAA CTCAGCTCTG CACCAAGCAG ACCTAATAGA
                                                  ORF2
     · I Y R   T L H   P K S   T E Y   T F F   S A P   H H T   Y S K   I D H   I V G   S K A
2901 CATCTACAGA ACTCTCCACC CCAAATCAAC AGAATATACA TTTTTTTCAG CACCACACCA CACCTATTCC AAAATTGACC ACATAGTTGG AAGTAAAGCT
                                                  ORF2
      L L S K   C K R   T E I   I T N Y   L S D   H S A   I K L E   I R I   K N L   T Q S H ·
3001 CTCCTCAGCA AATGTAAAAG AACAGAAATT ATAACAAACT ATCTCTCAGA CCACAGTGCA ATCAAACTAG AAATCAGGAT TAAGAATCTC ACTCAAAGCC
                                                  ORF2
     ·  S T T   W K L   N N L L   L N D   Y W V   H N E M   K A E   I K M   F F E T   N E N ·
3101 ACTCAACTAC ATGGAAACTG AACAACCTGC TCCTGAATGA CTACTGGGTA CATAACGAAA TGAAGGCAGA AATAAAGATG TTCTTTGAAA CCAACGAGAA
                                                  ORF2
     · K D T   T Y Q N   L W D   A F K   A V C R   G K F   I A L   N A Y K   R K Q   E R S
3201 CAAAGACACC ACATACCAGA ATCTCTGGGA CGCATTCAAA GCAGTGTGTA GAGGGAAATT TATAGCACTA AATGCCTACA AGAGAAAGCA GGAAAGATCC
                                                  ORF2
      K I D S   L T S   Q L K   E L E K   Q E Q   T H S   K A S R   R Q E   I T K   I R A E ·
3301 AAAATTGACA GCCTAACATC ACAATTAAAA GAACTAGAAA AGCAAGAGCA AACACATTCA AAAGCTAGCA GAAGGCAAGA AATAACTAAA ATCAGAGCAG
                                                  ORF2
     ·  L K E   I E T   Q K T L   Q K I   N E S   R S W F   F E R   I N K   I D R P   L A R ·
3401 AACTGAAGGA AATAGAGACA CAAAAAACCC TTCAAAAAAT CAATGAATCC AGGAGCTGGT TTTTTGAAAG GATCAACAAA ATTGATAGAC CACTAGCAAG
                                                  ORF2
     · L I K   K K R E   K N Q   I D T   I K N D   K G D   I T T   D P T E   I Q T   T I R
3501 ACTAATAAAG AAAAAAAGAG AGAAGAATCA AATAGACACA ATAAAAAATG ATAAAGGGGA TATCACCACC GATCCCACAG AAATACAAAC TACCATCAGA
                                                  ORF2
      E Y Y K   H L Y   A N K   L E N L   E E M   D T F   L D T Y   T L P   R L N   Q E E V ·
3601 GAATACTACA AACACCTCTA CGCAAATAAA CTAGAAAATC TAGAAGAAAT GGATACATTC CTCGACACAT ACACTCTCCC AAGACTAAAC CAGGAAGAAG
                                                  ORF2
     ·  E S L   N R P   I T G S   E I V   A I I   N S L P   T K K   S P G   P D G F   T A E ·
3701 TTGAATCTCT GAATAGACCA ATAACAGGCT CTGAAATTGT GGCAATAATC AATAGTTTAC CAACCAAAAA GAGTCCAGGA CCAGATGGAT TCACAGCCGA
                                                  ORF2
     · F Y Q   R Y K E   E L V   P F L   L K L F   Q S I   E K E   G I L P   N S F   Y E A
3801 ATTCTACCAG AGGTACAAGG AGGAACTGGT ACCATTCCTT CTGAAACTAT TCCAATCAAT AGAAAAAGAG GGAATCCTCC CTAACTCATT TTATGAGGCC
                                                  ORF2
      S I I L   I P K   P G R   D T T K   K E N   F R P   I S L M   N I D   A K I   L N K I ·
3901 AGCATCATTC TGATACCAAA GCCGGGCAGA GACACAACCA AAAAAGAGAA TTTTAGACCA ATATCCTTGA TGAACATTGA TGCAAAAATC CTCAATAAAA
                                                  ORF2
     ·  L A N   R I Q   Q H I K   K L I   H H D   Q V G F   I P G   M Q G   W F N I   R K S ·
4001 TACTGGCAAA CCGAATCCAG CAGCACATCA AAAAGCTTAT CCACCATGAT CAAGTGGGCT TCATCCCTGG GATGCAAGGC TGGTTCAATA TACGCAAATC
                                                  ORF2
     · I N V   I Q H I   N R A   K D K   N H M I   I S I   D A E   K A F D   K I Q   Q P F
4101 AATAAATGTA ATCCAGCATA TAAACAGAGC CAAAGACAAA AACCACATGA TTATCTCAAT AGATGCAGAA AAAGCCTTTG ACAAAATTCA ACAACCCTTC
                                                  ORF2
      M L K T   L N K   L G I   D G T Y   F K I   I R A   I Y D K   P T A   N I I   L N G Q ·
4201 ATGCTAAAAA CTCTCAATAA ATTAGGTATT GATGGGACGT ATTTCAAAAT AATAAGAGCT ATCTATGACA AACCCACAGC CAATATCATA CTGAATGGGC
                                                  ORF2
     ·  K L E   A F P   L K T G   T R Q   G C P   L S P L   L F N   I V L   E V L A   R A I ·
4301 AAAAACTGGA AGCATTCCCT TTGAAAACTG GCACAAGACA GGGATGCCCT CTCTCACCGC TCCTATTCAA CATAGTGTTG GAAGTTCTGG CCAGGGCAAT
                                                  ORF2
     · R Q E   K E I K   G I Q   L G K   E E V K   L S L   F A D   D M I V   Y L E   N P I
4401 CAGGCAGGAG AAGGAAATAA AGGGTATTCA ATTAGGAAAA GAGGAAGTCA AATTGTCCCT GTTTGCAGAC GACATGATTG TTTATCTAGA AAACCCCATC
                                                  ORF2
      V S A Q   N L L   K L I   S N F S   K V S   G Y K   I N V Q   K S Q   A F L   Y T N N ·
4501 GTCTCAGCCC AAAATCTCCT TAAGCTGATA AGCAACTTCA GCAAAGTCTC AGGATACAAA ATCAATGTAC AAAAATCACA AGCATTCTTA TACACCAACA
                                                  ORF2
     ·  R Q T   E S Q   I M G E   L P F   T I A   S K R I   K Y L   G I Q   L T R D   V K D ·
4601 ACAGACAAAC AGAGAGCCAA ATCATGGGTG AACTCCCATT CACAATTGCT TCAAAGAGAA TAAAATACCT AGGAATCCAA CTTACAAGGG ATGTGAAGGA
                                                  ORF2
     · L F K   E N Y K   P L L   K E I   K E D T   N K W   K N I   P C S W   V G R   I N I
4701 CCTCTTCAAG GAGAACTACA AACCACTGCT CAAGGAAATA AAAGAGGACA CAAACAAATG GAAGAACATT CCATGCTCAT GGGTAGGAAG AATCAATATC
                                                  ORF2
      V K M A   I L P   K V I   Y R F N   A I P   I K L   P M T F   F T E   L E K   T T L K ·
4801 GTGAAAATGG CCATACTGCC CAAGGTAATT TACAGATTCA ATGCCATCCC CATCAAGCTA CCAATGACTT TCTTCACAGA ATTGGAAAAA ACTACTTTAA
                                                  ORF2
```

```
     · F I W  N Q K  R A R I  A K S  I L S  Q K N K  A G G  I T L  P D F K  L Y Y ·
     ----------------------------------------------------------------------------------------------------
4901 AGTTCATATG GAACCAAAAA AGAGCCCGCA TCGCCAAGTC AATCCTAAGC CAAAAGAACA AAGCTGGAGG CATCACACTA CCTGACTTCA AACTATACTA

                                              ORF2
     ----------------------------------------------------------------------------------------------------
     ·K A T  V T K T  A W Y  W Y Q  N R D I  D Q W  N R T  E P S E  I M P  H I Y
     ----------------------------------------------------------------------------------------------------
5001 CAAGGCTACA GTAACCAAAA CAGCATGGTA CTGGTACCAA AACAGAGATA TAGATCAATG GAACAGAACA GAGCCCTCAG AAATAATGCC GCATATCTAC

                                              ORF2
     ----------------------------------------------------------------------------------------------------
      N Y L I  F D K  P E K  N K Q W  G K D  S L F  N K W C  W E N  W L A  I C R K ·
     ----------------------------------------------------------------------------------------------------
5101 AACTATCTGA TCTTTGACAA ACCTGAGAAA AACAAGCAAT GGGGAAAGGA TTCCCTATTT AATAAATGGT GCTGGGAAAA CTGGCTAGCC ATATGTAGAA

                                              ORF2
     ----------------------------------------------------------------------------------------------------
     · L K L  D P F  L T P Y  T K I  N S R  W I K D  L N V  R P K  T I K T  L E E ·
     ----------------------------------------------------------------------------------------------------
5201 AGCTGAAACT GGATCCCTTC CTTACACCTT ATACAAAAAT CAATTCAAGA TGGATTAAAG ATTTAAACGT TAGACCTAAA ACCATAAAAA CCCTAGAAGA

                                              ORF2
     ----------------------------------------------------------------------------------------------------
     ·N L G  I T I Q  D I G  V G K  D F M S  K T P  K A M  A T K A  K I D  K W D
     ----------------------------------------------------------------------------------------------------
5301 AAACCTAGGC ATTACCATTC AGGACATAGG CGTGGGCAAG GACTTCATGT CCAAAACACC AAAAGCAATG GCAACAAAAG CCAAAATTGA CAAATGGGAT

                                              ORF2
     ----------------------------------------------------------------------------------------------------
      L I K L  K S F  C T A  K E T T  I R V  N R Q  P T T W  E K I  F A T  Y S S D ·
     ----------------------------------------------------------------------------------------------------
5401 CTAATTAAAC TAAAGAGCTT CTGCACAGCA AAAGAAACTA CCATCAGAGT GAACAGGCAA CCTACAACAT GGGAGAAAAT TTTCGCAACC TACTCATCTG

                                              ORF2
     ----------------------------------------------------------------------------------------------------
     · K G L  I S R  I Y N E  L K Q  I Y K  K K T N  N P I  K K W  A K D M  N R H ·
     ----------------------------------------------------------------------------------------------------
5501 ACAAAGGGCT AATATCCAGA ATCTACAATG AACTCAAACA AATTTACAAG AAAAAAACAA ACAACCCCAT CAAAAAGTGG GCGAAGGACA TGAACAGACA

                                              ORF2
     ----------------------------------------------------------------------------------------------------
     ·F S K  E D I Y  A A K  K H M  K K C S  S S L  A I R  E M Q I  K T T  M R Y
     ----------------------------------------------------------------------------------------------------
5601 CTTCTCAAAA GAAGACATTT ATGCAGCCAA AAAACACATG AAGAAATGCT CATCATCACT GGCCATCAGA GAAATGCAAA TCAAAACCAC TATGAGATAT

                                              ORF2
     ----------------------------------------------------------------------------------------------------
      H L T P  V R M  A I I  K K S G  N N R  C W R  G C G E  I G T  L L H  C W W D ·
     ----------------------------------------------------------------------------------------------------
5701 CATCTCACAC CAGTTAGAAT GGCAATCATT AAAAAGTCAG GAAACAACAG GTGCTGGAGA GGATGCGGAG AAATAGGAAC ACTTTTACAC TGTTGGTGGG

                                              ORF2
     ----------------------------------------------------------------------------------------------------
     · C Q L  V Q P  L W K S  V W R  F L R  D L E L  E I P  F D P  A I P L  L G I ·
     ----------------------------------------------------------------------------------------------------
5801 ACTGTCAACT AGTTCAACCA TTGTGGAAGT CAGTGTGGCG ATTCCTCAGG GATCTAGAAC TAGAAATACC ATTTGACCCA GCCATCCCAT TACTGGGTAT

                                              ORF2
     ----------------------------------------------------------------------------------------------------
     ·Y P K  D Y K S  C C Y  K D T  C T R M  F I A  A L F  T I A K  T W N  Q P K
     ----------------------------------------------------------------------------------------------------
5901 ATACCCAAAG GACTATAAAT CATGCTGCTA TAAAGACACA TGCACACGTA TGTTTATTGC GGCACTATTC ACAATAGCAA AGACTTGGAA CCAACCCAAA

                                              ORF2
     ----------------------------------------------------------------------------------------------------
      C P T M  I D W  I K K  M W H I  Y T M  E Y Y  A A I K  N D E  F I S  F V G T ·
     ----------------------------------------------------------------------------------------------------
6001 TGTCCAACAA TGATAGACTG GATTAAGAAA ATGTGGCACA TATACACCAT GGAATACTAT GCAGCCATAA AAAATGATGA GTTCATATCC TTTGTAGGGA

                                         ORF2
     ---------------------------------------------------------------------------------------
     · W M K  L E T  I I L S  K L S  Q E Q  K T K H  R I F  S L I  G G N
     ---------------------------------------------------------------------------------------
6101 CATGGATGAA ATTGGAAACC ATCATTCTCA GTAAACTATC GCAAGAACAA AAAACCAAAC ACCGCATATT CTCACTCATA GGTGGGAATT GAACAATGAG
6201 ATCACATGGA CACAGGAAGG GGAATATCAC ACTCTGGGGA CTGTGGTGGG GTCGGGGGAG GGGGGAGGGA TAGCATTGGG AGATATACCT AATGCTAGAT
                                                                                                        TSD
                                                                                                      ~~~~~
6301 GACACATTAG TGGGTGCAGC GCACCAGCAT GGCACATGTA TACATATGTA ACTAACCTGC ACAATGTGCA CATGTACCCT AAAACTTAGA TTATACTTAC
     TSD
     ~~
6401 TGTGCTATTC TTGTAGAGTG GCTCAGCAGT GGTAATTTTT CTTTGAATGC AGTAGTTCAG ATTGGGATGT ACACTTGATT CTTCCTTCTT CACTGTAAAT
6501 CTGTAAAGTT TCCATGAACG CCAAGTTAAT TGAACATTGA ATGATCTACT CCCACAGGAA ATAAAGAGTC AGATTCCTAT AAGCCTGTGA TAACAGTATT
6601 TTTGTCCAGC TGATCAAAAC ATAACCTTGT CTTATATGTG TTTCTTTTTA AAGATAACGT ATTTAATCAT GTATCATTGA TTCATTAACT TTGGACTCAT
6701 GGCCAACAGC ATGAATGAAG CTTATCCADT TTTCTCCATA AGGCACATAG CAGCCCTCTT GTGCTCAGGA ACACTAGATA GAGTTTCTGC TATTACACTT
6801 GGGGACCATT TTAAACAGCA AAATCACCAA CAAAAAGCAC AAAAATTCAA CATAACGTGG TACTAAATAG ATCATGAAAA GGACACTTGT TTACGGTAAG
6901 AGCTGAAACA ACAGAGGTAG AATATTGCCT TGTTTGACCT CAGCTGTGCA CTTGCACGTG CGGCAACTCG AACTTTTTTT TTGCCACACT GTGCTAATGA
7001 CCACTAATGA TCACAGACAT GCTGCAAATA CTGDATTTCA GGGTTACAAA TAAATTTAAG TGAGTAGGCA ACTTCCCAAA TATGGAATCC ACACAATAAT
7101 GAGGATTTCC CAGACTGTAA TGTCTGGGAA AAAATGCTGA TTGCTCTAAA GTABTCTTTA AAAGGGAAAT TGATGCTTTG GTTATGATGT CCTGGCTCTG
7201 CCAGAAATGA TAGCCTCCTT GTATTTTTCC TACTCCATCA TTCCAGAGGA GACAGGATCA GAATCTCTGC CCCCTTGAGC CTACTTTTTG CATCAGATGT
7301 TTGCTGTGAG AGTATGAGTC TGTAAA
```

L1-dn6-5.4

```
  1  CGGAATTCGA TTAAAAGATT GGAAAACAGT TGCAGAACAT GAAATGAAGA TAGATTTTAT ATACATGAAA AGATGTTCAG TCTCACATAT GAGAAAAACG

           5'TSD
         ~~~~~~~~~~~~~
101  CAATTTAAAA CTATACTGGG GGGGAGGAGC CAAGATGGCC GAATAGGAAC AGCTCCGGTC TACAGCTCCC TGCGTGAGCG ACGCAGAAGA CGGTGATTTC
201  TGCATTTCCA TCTGAGGTAC CGGGTTCATC TCACTAGGGA GTGCCAGACA GTGGGCGCAG GCCAGTGTGT GTGCGCACCG TGCGCGAGCC GAAGCAGGGC
301  GAGGCATTGC CTCACCTGGG AAGCGCAAGG GGTCAGGGAG TTCCCTTTCC GAGTCAAAGA AAGGGGTGAC GGACGCACCT GGAAAATCGG GTCACTCCCA
401  CCCGAATATT GTGCTTTTCA GACCGGCTTA AGAAACGGCG CACCACGAGA CTATATCCCA CACCTGGCTC GGAGGGTCCT ACGCCCATGG AATCTCGCTG
501  ATTGCTAGCA CAGCAGTCTG AGATCAAACT GCAAGGCGGC AACGAGGCTG GGGGAGGGGC GCCCGCCATT GCCCAGGCTT GCTTAGGTAA ACAAAGCAGC
601  CGGGAAGCTC GAACTGGGTG GAGCCCACCA CAGCTCAAGG AGGCCTGCCT GCCTCTGTAG GCTCCACCTC TGGGGGCAGG GCACAGACAA ACAAAAAGAC
701  AGCAGTAACC TCTGCAGACT TAAGTGTCCC TGTCTGACAG CTTTGAAGAG AGCAGTGGTT CTCCCAGCAC GCAGCTGGAG ATCTGAGAAC GGGCAGACTG
801  CCTCCTCAAG TGGGTCCCTG ACTCCTGACC CCCGAGCAGC CTAACTGGGA GGCACCCCCC AGCAGGGGCA CACTGACACC TCACACGGCA GGGTATTCCA
901  ACAGACCTGC AGCTGAGGGT CCTGTCTGTT AGAAGGAAAA CTAACAACCA GAAAGGACAT CTACACCGAA AACCCATCTG TACATCACCA TCATCAAAGA
```

ORF1

M G K K Q N R K T G N S K T Q S A S P P P K E R S S ·

1001 CCAAAAGTAG ATAAAACCAC AAAGATGGGG AAAAAACAGA ACAGAAAAAC TGGAAACTCT AAAACACAGA GCGCCTCTCC TCCTCCAAAG GAACGCAGTT

ORF1

· S P A T E Q S W M E N D F D E L R E E G F R R S N Y S E L R E D I ·

1101 CCTCACCAGC AACAGAACAA AGCTGGATGG AGAATGATTT TGACGAGCTG AGAGAAGAAG GCTTCAGACG ATCAAATTAC TCTGAGCTAC GGGAGGACAT

ORF1

· Q T K G K E V E N F E K N L E E C I T R I T N T E K C L K E L M E

1201 TCAAACCAAA GGCAAAGAAG TTGAAAACTT TGAAAAAAAT TTAGAAGAAT GTATAACTAG AATAACCAAT ACAGAGAAGT GCTTAAAGGA GCTGATGGAG

ORF1

L K T K A R E L R E E C R S L R S R C D Q L E E R V S A M E D E M N ·

1301 CTGAAAACCA AGGCTCGAGA ACTACGTGAA GAATGCAGGA GCCTCAGGAG CCGATGCGAT CAACTGGAAG AAAGGGTATC AGCAATGGAA GATGAAATGA

ORF1

· E M K R E G K F R E K R I K R **D** E Q S L Q E I W D Y V K R P N L R ·

1401 ATGAAATGAA GCGAGAAGGG AAGTTTAGAG AAAAAAGAAT AAAAAGAGAT GAGCAAAGCC TCCAAGAAAT ATGGGACTAT GTGAAAAGAC CAAATCTACG

ORF1

· L I G V P E S D **G** E N G T K L E N T L Q D I I Q E N F P N L A R Q

1501 TCTGATTGGT GTACCTGAAA GTGATGGGGA GAATGGAACC AAGTTGGAAA ACACTCTGCA GGATATTATC CAGGAGAACT TCCCCAATCT AGCAAGGCAG

ORF1

A N V Q I Q E I Q R T P Q R Y S S R R A T P R H I I V R F T K V E M ·

1601 GCCAACGTTC AGATTCAGGA AATACAGAGA ACGCCACAAA GATACTCCTC GAGAAGAGCA ACTCCAAGAC ACATAATTGT CAGATTCACC AAAGTTGAAA

ORF1

· K E K M L R A A R E K G R V T L K G K P I R L T **A** D L S A E T L Q ·

1701 TGAAGGAAAA AATGTTAAGG GCAGCCAGAG AGAAAGGTCG GGTTACCCTC AAAGGAAAGC CCATCAGACT AACAGCGGAT CTCTCGGCAG AAACCCTACA

ORF1

· A R R E W G P I F N I L K E K N F Q P R I S Y P A K L S F I S E G

1801 AGCCAGAAGA GAGTGGGGGC CAATATTCAA CATTCTTAAA GAAAAGAATT TTCAACCCAG AATTTCATAT CCAGCCAAAC TAAGCTTCAT AAGTGAAGGA

ORF1

E I K Y F I D K Q M L R D F V T T R P A L K E L L K E A L N M E R N ·

1901 GAAATAAAAT ACTTTATAGA CAAGCAAATG TTGAGAGATT TTGTCACCAC CAGGCCTGCC CTAAAAGAGC TCCTGAAGGA AGCGCTAAAC ATGGAAAGGA

ORF1

· N R Y Q P L Q N H A K M

2001 ACAACCGGTA CCAGCCGCTG CAAAATCATG CCAAAATGTA AAGACCATCG AGACTAGGAA GAAACTGCAT CAACTAATGA GCAAAATCAC CAGCTAACAT

ORF2

M T G S N S H I T I L T L N I N G L N S A I K R H R L A S W I K

2101 CATAATGACA GGATCAAATT CACACATAAC AATATTAACT TTAAATATAA ATGGACTAAA TTCTGCAATT AAAAGACACA GACTGGCAAG TTGGATAAAG

ORF2

S Q D P S V C C I Q E T H L T C R D T H R L K I K G W R K I Y Q A N ·

2201 AGTCAAGACC CATCAGTGTG CTGTATTCAG GAAACCCATC TCACGTGCAG AGACACACAT AGGCTCAAAA TAAAAGGATG GAGGAAGATC TACCAAGCCA

ORF2

· G K Q K K A G V A I L V S D K T D F K P T K I **E** R D K E G H Y I M ·

2301 ATGGAAAACA AAAAAAGGCA GGGGTTGCAA TCCTAGTCTC TGATAAAACA GACTTTAAAC CAACAAAGAT CGAAAGAGAC AAAGAAGGCC ATTACATAAT

ORF2

· V K G S I Q Q E E L T I L N I Y A P N T G A P R F I K Q V L S D L

2401 GGTAAAGGGA TCAATTCAAC AAGAGGAGCT AACTATCCTA AATATTTATG CACCCAATAC AGGAGCACCC AGATTCATAA AGCAAGTCCT CAGTGACCTA

ORF2

Q R D L D S H T L I M G D F N T P L S T L D R S T R Q K V N K D T Q ·

2501 CAAAGAGACT TAGACTCCCA CACATTAATA ATGGGAGACT TTAACACCCC ACTGTCAACA TTAGACAGAT CAACAAGACA GAAAGTCAAC AAGGATACCC

ORF2

· E L N S A L H Q A D L I D I Y R T L H P K S T E Y T F F S A P H H ·

2601 AGGAATTGAA CTCAGCTCTG CACCAAGCAG ACCTAATAGA CATCTACAGA ACTCTCCACC CCAAATCAAC AGAATATACA TTTTTTTCAG CACCACACCA

ORF2

· T Y S K I D H I V G S K A L L S K C K R T E I I T N Y L S D H S A

2701 CACCTATTCC AAAATTGACC ACATAGTTGG AAGTAAAGCT CTCCTCAGCA AATGTAAAAG AACAGAAATT ATAACAAACT ATCTCTCAGA CCACAGTGCA

ORF2

I K L E L R I K N L T Q S **R** A T T W K L N N L L L N D Y W V H N E M ·

2801 ATCAAACTAG AACTCAGGAT TAAGAATCTC ACTCAAAGCC GCGCAACTAC ATGGAAACTG AACAACCTGC TCCTGAATGA CTACTGGGTA CATAACGAAA

ORF2

· K A E I K M F F E T N E N K D T T Y Q N L W D A F K A V C R G K F ·

```
2901  TGAAGGCAGA AATAAAGATG TTCTTTGAAA CCAACGAGAA CAAAGACACC ACATACCAGA ATCTCTGGGA CGCATTCAAA GCAGTGTGTA GAGGGAAATT
                                                ORF2
      ------------------------------------------------------------------------------------------------------------
      ·I  A  L   N  A  Y  K   R  K  Q   E  R  S   K  I  D  T   L  T  S   Q  L  K   E  L  E  K   Q  E  Q   T  H  S
      ------------------------------------------------------------------------------------------------------------
3001  TATAGCACTA AATGCCTACA AGAGAAAGCA GGAAAGATCC AAAATTGACA CCCTAACATC ACAATTAAAA GAACTAGAAA AGCAAGAGCA AACACATTCA
                                                ORF2
      ------------------------------------------------------------------------------------------------------------
       K  A  S  R   R  Q  E   I  T  K   I  R  A  E   L  K  E   I  E  T   Q  K  T  L   Q  K  I   N  E  S   R  S  W  F·
      ------------------------------------------------------------------------------------------------------------
3101  AAAGCTAGCA GAAGGCAAGA AATAACTAAA ATCAGAGCAG AACTGAAGGA AATAGAGACA CAAAAAACCC TTCAAAAAAT CAATGAATCC AGGAGCTGGT
                                                ORF2
      ------------------------------------------------------------------------------------------------------------
      · F  E  R   I  N  K   I  D  R  P   L  A  R   L  I  K   K  K  R  E   K  N  Q   I  D  T   I  K  N  D   K  G  D·
      ------------------------------------------------------------------------------------------------------------
3201  TTTTTGAAAG GATCAACAAA ATTGATAGAC CGCTAGCAAG ACTAATAAAG AAAAAAAGAG AGAAGAATCA AATAGACACA ATAAAAAATG ATAAAGGGGA
                                                ORF2
      ------------------------------------------------------------------------------------------------------------
      ·I  T  T   D  P  T  E   I  Q  T   T  I  R   E  Y  Y  K   H  L  Y   A  N  K   L  E  N  L   E  E  M   D  T  F
      ------------------------------------------------------------------------------------------------------------
3301  TATCACCACC GATCCCACAG AAATACAAAC TACCATCAGA GAATACTACA AACACCTCTA CGCAAATAAA CTAGAAAATC TGGAAGAAAT GGATACATTC
                                                ORF2
      ------------------------------------------------------------------------------------------------------------
       L  D  T  Y   T  L  P   R  L  N   Q  E  E  V   E  S  L   N  R  P   T  T  G  S   E  I  V   A  I  I   N  S  L  P·
      ------------------------------------------------------------------------------------------------------------
3401  CTCGACACAT ACACTCTCCC AAGACTAAAC CAGGAAGAAG TTGAATCTCT GAATCGACCA ACAACAGGCT CTGAAATTGT GGCAATAATC AATAGTTTAC
                                                ORF2
      ------------------------------------------------------------------------------------------------------------
      · T  K  K   S  P  G   P  D  G  F   T  A  E   F  Y  Q   R  Y  K  E   E  L  V   P  F  L   L  K  L  F   Q  S  I·
      ------------------------------------------------------------------------------------------------------------
3501  CAACCAAAAA GAGTCCAGGA CCAGATGGAT TCACAGCCGA ATTCTACCAG AGGTACAAGG AGGAACTGGT ACCATTCCTT CTGAAACTAT TCCAATCAAT
                                                ORF2
      ------------------------------------------------------------------------------------------------------------
      ·E  K  E   G  I  L  P   N  S  F   Y  E  A   S  I  I  L   I  P  K   P  G  R   D  T  T  K   K  E  N   F  R  P
      ------------------------------------------------------------------------------------------------------------
3601  AGAAAAAGAG GGAATCCTCC CTAACTCATT TTATGAGGCC AGCATCATTC TGATACCAAA GCCGGGCAGA GACACAACCA AAAAAGAGAA TTTTAGACCA
                                                ORF2
      ------------------------------------------------------------------------------------------------------------
       I  S  L  M   N  I  D   A  K  I   L  N  K  I   L  A  N   R  I  Q   Q  H  I  K   K  L  I   H  H  D   Q  V  G  F·
      ------------------------------------------------------------------------------------------------------------
3701  ATATCCTTGA TGAACATTGA TGCAAAAATC CTCAATAAAA TACTGGCAAA CCGAATCCAG CAGCACATCA AAAAGCTTAT CCACCATGAT CAAGTGGGCT
                                                ORF2
      ------------------------------------------------------------------------------------------------------------
      · I  P  G   M  Q  G   W  F  N  I   R  K  S   I  N  V   I  Q  H  I   N  R  A   K  D  K   N  H  M  I   I  S  I·
      ------------------------------------------------------------------------------------------------------------
3801  TCATCCCTGG GATGCAAGGC TGGTTCAATA TACGCAAATC AATAAATGTA ATCCAGCATA TAAACAGAGC CAAAGACAAA AACCACATGA TTATCTCAAT
                                                ORF2
      ------------------------------------------------------------------------------------------------------------
      ·D  A  E   K  A  F  D   K  I  Q   Q  P  F   M  L  K  T   L  N  K   L  G  I   D  G  T  Y   F  K  I   I  R  A
      ------------------------------------------------------------------------------------------------------------
3901  AGATGCAGAA AAAGCCTTTG ACAAAATTCA ACAACCCTTC ATGCTAAAAA CTCTCAATAA ATTAGGTATT GATGGGACGT ATTTCAAAAT AATAAGAGCT
                                                ORF2
      ------------------------------------------------------------------------------------------------------------
       I  Y  D  K   P  T  A   N  I  I   L  N  G  Q   K  L  E   A  F  P   L  K  T  G   T  R  Q   G  C  P   L  S  P  L·
      ------------------------------------------------------------------------------------------------------------
4001  ATCTATGACA AACCCACAGC CAATATCATA CTGAATGGGC AAAAACTGGA AGCATTCCCT TTGAAAACTG GCACAAGACA GGGATGCCCT CTCTCACCGC
                                                ORF2
      ------------------------------------------------------------------------------------------------------------
      · L  F  N   I  V  L   E  V  L  A   R  A  I   R  Q  E   K  E  I  K   G  I  Q   L  G  K   E  E  V  K   L  S  L·
      ------------------------------------------------------------------------------------------------------------
4101  TCCTATTCAA CATAGTGTTG GAAGTTCTGG CCAGGGCAAT CAGGCAGGAG AAGGAAATAA AGGGTATTCA ATTAGGAAAA GAGGAAGTCA AATTGTCCCT
                                                ORF2
      ------------------------------------------------------------------------------------------------------------
      ·F  A  D   D  M  I  V   Y  L  E   N  P  I   V  S  A  Q   N  L  L   K  L  I   S  N  F  S   K  V  S   G  Y  K
      ------------------------------------------------------------------------------------------------------------
4201  GTTTGCAGAC GACATGATTG TTTATCTAGA AAACCCCATC GTCTCAGCCC AAAATCTCCT TAAGCTGATA AGCAACTTCA GCAAAGTCTC AGGATACAAA
                                                ORF2
      ------------------------------------------------------------------------------------------------------------
       I  N  V  Q   K  S  Q   A  F  L   Y  T  N  N   R  Q  T   E  S  Q   I  M  S  E   L  P  F   T  I  A   S  K  R  I·
      ------------------------------------------------------------------------------------------------------------
4301  ATCAATGTAC AAAAATCACA AGCATTCTTA TACACCAACA ACAGACAAAC AGAGAGCCAA ATCATGAGTG AACTCCCATT CACAATTGCT TCAAAGAGAA
                                                ORF2
      ------------------------------------------------------------------------------------------------------------
      · K  Y  L   G  I  Q   L  T  R  D   V  K  D   L  F  K   E  N  Y  K   P  L  L   K  E  I   K  E  E  T   N  K  W·
      ------------------------------------------------------------------------------------------------------------
4401  TAAAATACCT AGGAATCCAA CTTACAAGGG ATGTGAAGGA CCTCTTCAAG GAGAACTACA AACCACTGCT CAAGGAAATA AAAGAGGAGA CAAACAAATG
                                                ORF2
      ------------------------------------------------------------------------------------------------------------
      ·K  N  I   P  C  S  W   V  G  R   I  N  I   V  K  M  A   I  L  P   K  V  I   Y  R  F  N   A  I  P   I  K  L
      ------------------------------------------------------------------------------------------------------------
4501  GAAGAACATT CCATGCTCAT GGGTAGGAAG AATCAATATC GTGAAAATGG CCATACTGCC CAAGGTAATT TACAGATTCA ATGCCATCCC CATCAAGCTA
                                                ORF2
      ------------------------------------------------------------------------------------------------------------
       P  M  T  F   F  T  E   L  E  K   T  T  L  K   F  I  W   N  Q  K   R  A  R  I   A  K  S   I  L  S   Q  K  N  K·
      ------------------------------------------------------------------------------------------------------------
4601  CCAATGACTT TCTTCACAGA ATTGGAAAAA ACTACTTTAA AGTTCATATG GAACCAAAAA AGAGCCCGCA TTGCCAAGTC AATCCTAAGC CAAAAGAACA
                                                ORF2
      ------------------------------------------------------------------------------------------------------------
      · A  G  G   I  T  L   P  D  F  K   L  Y  Y   K  A  T   V  T  K  T   A  W  Y   W  Y  Q   N  R  D  I   D  Q  W·
      ------------------------------------------------------------------------------------------------------------
4701  AAGCTGGAGG CATCACACTA CCTGACTTCA AACTATACTA CAAGGCTACA GTAACCAAAA CAGCATGGTA CTGGTACCAA AACAGAGATA TAGATCAATG
                                                ORF2
      ------------------------------------------------------------------------------------------------------------
      ·N  I  T   E  P  S  E   I  M  P   H  I  Y   N  Y  L  I   F  D  K   P  E  K   N  Q  Q  W   G  K  D   S  L  F
      ------------------------------------------------------------------------------------------------------------
4801  GAACATAACA GAGCCCTCAG AAATAATGCC GCATATCTAC AACTATCTGA TCTTTGACAA ACCTGAGAAA AACCAGCAAT GGGGAAAGGA TTCCCTGTTT
                                                ORF2
      ------------------------------------------------------------------------------------------------------------
       N  K  W  C   W  E  N   W  L  A   I  C  R  K   L  K  L   D  P  F   L  T  P  Y   T  K  I   N  S  R   W  I  K  D·
```

```
4901  AATAAATGGT GCTGGGAAAA CTGGCTAGCC ATATGTAGAA AGCTGAAACT GGATCCCTTC CTTACACCTT ATACAAAAAT CAATTCAAGA TGGATTAAAG

                                                      ORF2
      · L N V  K P K  T I K T  L E E  N L G  I T I Q  D I G  V G K  D F M S  K T P ·
5001  ATTTAAACGT TAAACCTAAA ACCATAAAAA CCCTAGAAGA AAACCTAGGC ATTACCATTC AGGACATAGG CGTGGGCAAG GACTTCATGT CCAAAACACC

                                                      ORF2
      ·K A M  A T K D  K I D  K W D  L I K L  K S F  C T A  K E T T  I R V  N R Q
5101  AAAAGCAATG GCAACAAAAG ACAAAATTGA CAAATGGGAT CTAATTAAAC TAAAGAGTTT CTGCACAGCA AAAGAAACTA CCATCAGAGT GAACAGGCAA

                                                      ORF2
       P T T W  E K I  F A T  Y S S D  K G L  I S R  I Y N E  L K Q  I Y K  K K T N ·
5201  CCTACAACAT GGGAGAAAAT TTTTGCAACC TACTCATCTG ACAAAGGGCT AATATCCAGA ATCTACAATG AACTCAAACA AATTTACAAG AAAAAAACAA

                                                      ORF2
      · N P I  K K W  A K D M  N R H  F S K  E D I Y  A A K  K H M  K K C S  S S L ·
5301  ACAACCCCAT CAAAAAGTGG GCGAAGGACA TGAACAGACA CTTCTCAAAA GAAGACATTT ATGCAGCCAA AAAACACATG AAGAAATGCT CATCATCACT

                                                      ORF2
      ·A I R  E M Q I  K T T  M R Y  H L T P  V R M  A I I  K K S G  N N R  C W R
5401  GGCCATCAGA GAAATGCAAA TCAAAACCAC TATGAGATAT CATCTCACAC CAGTTAGAAT GGCAATCATT AAAAAGTCAG GAAACAACAG GTGCTGGAGA

                                                      ORF2
       G C G E  I G T  L L H  C W W D  C K L  V Q P  L W K S  V W R  F L R  D L E L ·
5501  GGATGCGGAG AAATAGGAAC ACTTTTACAC TGTTGGTGGG ACTGTAAACT AGTTCAACCA TTGTGGAAGT CAGTGTGGCG ATTCCTCAGG GATCTAGAAC

                                                      ORF2
      · E I P  F D P  A I P L  L G I  Y P N  E Y K S  C C Y  K D T  C T R M  F I A ·
5601  TAGAAATACC ATTTGACCCA GCCATCCCAT TACTGGGTAT ATACCCAAAT GAGTATAAAT CATGCTGCTA TAAAGACACA TGCACACGTA TGTTTATTGC

                                                      ORF2
      ·A L F  T I A K  T W N  Q P K  C P T M  I D W  I K K  M W H I  Y T M  E Y Y
5701  GGCACTATTC ACAATAGCAA AGACTTGGAA CCAACCCAAA TGTCCAACAA TGATAGACTG GATTAAGAAA ATGTGGCACA TATACACCAT GGAATACTAT

                                                      ORF2
       A A I K  S D E  F I S  F V G T  W M K  L E T  I I L S  K L S  Q E Q  K T K H ·
5801  GCAGCCATAA AAAGTGATGA GTTCATATCC TTTGTAGGGA CATGGATGAA ATTGGAAACC ATCATTCTCA GTAAACTATC GCAAGAACAA AAAACCAAAC

              ORF2
      · R I F  S L I  G G N
5901  ACCGCATATT CTCACTCATA GGTGGGAATT GAACAATGAG ATCACATGGA CACAGGAAGG GGAATATCAC ACTCTGGGGA CTGTGGTGGG GTCGGGGGAG
6001  GGGGGAGGGA TAGCATTGGG AGATATACCT AATGCTAGAT GACACATTAG TGGGTGCAGC GCACCAGCAT GGCACATGTA TACATATGTA ACTAACCTGC
6101  ACAATGTGCA CATGTACCCT AAAACTTAGA GTATAATAAA AAAAAAAAAA AAAAAAAAAA AAAAAAAAAA AAAAAAAAAA AAAAAAAAAA AAAAAAAAAA

         3'TSD
      ~~~~~~~~~~~~~~
6201  AAAAACTATA CTGAATGCTA TCTTTCATAT TTCATGTTGG CAAAACATGA AAAATCTCAC ATAATTTTGC TTTTATGAAG CAAAGCTTTT ATGAAGCAAA
6301  CCTTTTATGA AGCAAAACTG AATAATGCAA GATACAAACG AAGGCTTATT TGTTTAAAAA TGAATAAAAA GAAATATATA TTCTAACAGA CTTTGTTGAG
6401  TCTTTAGGTT TTTCTAAATA TAAGATTATG TCCTCTGTGA ACAAGAATAA TTGGACTTAT TCCAAAGTTG CAGG
```

L1-dn6-2.2

```
   1  CGGAATTCGA TTAAAAGATT GGAAAACAGT TGCAGAACAT GAAATGAAGA TAGATTTTAT ATACATGAAA AGATGTTCAG TCTCACATAT GAGAAAAACG
               5' TSD
            ~~~~~~~~~~~~~
 101  CAATTTAAAA CTATACTGGG GGGGAGGAGC CAAGATGGCC GAATAGGAAC AGCTCCGGTC TACAGCTCCC TGCGTGAGCG ACGCAGAAGA CGGTGATTTC
 201  TGCATTTCCA TCTGAGGTAC CGGGTTCATC TCACTAGGGA GTGCCAGACA GTGGGCGCAG GCCAGTGTGT GTGCGCACCG TGCGCGAGCC GAAGCAGGGC
 301  GAGGCATTGC CTCACCTGGG AAGCGCAAGG GGTCAGGGAG TTCCCTTTCC GAGTCAAAGA AAGGGGTGAC GGACGCACCT GGAAAATCGG GTCACTCCCA
 401  CCCGAATATT GCGCTTTTCA GACCGGCTTA AGAAACGGCG CGCCACGAGA CTATATCCCA CACCTGGCTC GGAGGGTCCT ACGCCCATGG AATCTCGCTG
 501  ATTGCTAGCA CAGCAGTCTG AGATCAAACT GCAAGGCGGC AACGAGGCTG GGGGAGGGGC GCCCGCCATT GCCCAGGCTT GCTTAGGTAA ACAAAGCAGC
 601  CGGGAAGCTC GAACTGGGTG GAGCCCACCA CAGCTCAAGG AGGCCTGCCT GCCTCTGTAG GCTCCACCTC TGGGGGCAGG GCACAGACAA ACAAAAAGAC
 701  AGCAGTAACC TCTGCAGACT TAAGTGTCCC TGTCTGACAG CTTTGAAGAG AGCAGTGGTT CTCCCAGCAC GCAGCTGGAG ATCTGAGAAC GGGCAGACTG
 801  CCCCCTCAAG TGGGTCCCTG ACTCCTGACC CCCGAGCAGC CTAACTGGGA GGCACCCCCC AGCAGGGGCA CACTGACACC TCACACGGCA GGGTATTCCA
 901  ACAGACCTGC AGCTGAGGGC CCTGTCTGTT AGAAGGAAAA CTAACAACCA GAAAGGACAT CTACACCGAA AACCCATCTG TACATCACCA TCATCAAAGA
                                                                         Orf1
                                M  G   K  K  Q  N   R  K  T   G  N  S   K  T  Q  S   A  S  P   P  P  K   E  R  S  S ·
1001  CCAAAAGTAG ATAAAACCAC AAAGATGGGG AAAAAACAGA ACAGAAAAAC TGGAAACTCT AAAACACAGA GCGCCTCTCC TCCTCCAAAG GAACGCAGTT
                                                        Orf1
      · S  P  A   T  E  Q   S  W  M  E   N  D  F   D  E  L   R  E  E  G   F  R  R   S  N  Y   S  E  L  R   E  D  I ·
1101  CCTCACCAGC AACAGAACAA AGCTGGATGG AGAATGATTT TGACGAGCTG AGAGAAGAAG GCTTCAGACG ATCAAATTAC TCTGAGCTAC GGGAGGACAT
                                                        Orf1
      ·Q  T  K   G  K  E  V   E  N  F   E  K  N   L  E  E  C   I  T  R   I  T  N   T  E  K  C   L  K  E   L  M  E
1201  TCAAACCAAA GGCAAAGAAG TTGAAAACTT TGAAAAAAAT TTAGAAGAAT GTATAACTAG AATAACCAAT ACAGAGAAGT GCTTAAAGGA GCTGATGGAG
                                                        Orf1
       L  K  T  K   A  R  E   L  R  E   E  C  R  S   L  R  S   R  C  D   Q  L  E  E   R  V  S   A  M  E   D  E  M  N ·
1301  CTGAAAACCA AGGCTCGAGA ACTACGTGAA GAATGCAGGA GCCTCAGGAG CCGATGCGAT CAACTGGAAG AAAGGGTATC AGCAATGGAA GATGAAATGA
                                                        Orf1
      · E  M  K   R  E  G   K  F  R  E   K  R  I   K  R  N   E  Q  S  L   Q  E  I   W  D  Y   V  K  R  P   N  L  R ·
1401  ATGAAATGAA GCGAGAAGGG AAGTTTAGAG AAAAAAGAAT AAAAAGAAAT GAGCAAAGCC TCCAAGAAAT ATGGGACTAT GTGAAAAGAC CAAATCTACG
                                                        Orf1
      ·L  I  G   V  P  E  S   D  G  E   N  G  T   K  L  E  N   T  L  Q   D  I  I   Q  E  N  F   P  N  L   A  R  Q
1501  TCTGATTGGT GTACCTGAAA GTGATGGGGA GAATGGAACC AAGTTGGAAA ACACTCTGCA GGATATTATC CAGGAGAACT TCCCCAATCT AGCAAGGCAG
                                                        Orf1
       A  N  V  Q   I  Q  E   I  Q  R   T  P  Q  R   Y  S  S   R  R  A   T  P  R  H   I  I  V   R  F  T   K  V  E  M ·
1601  GCCAACGTTC AGATTCAGGA AATACAGAGA ACGCCACAAA GATACTCCTC GAGAAGAGCA ACTCCAAGAC ACATAATTGT CAGATTCACC AAAGTTGAAA
                                                        Orf1
      · K  E  K   M  L  R   A  A  R  E   K  G  R   V  T  L   K  G  K  P   I  R  L   T  A  D   L  S  A  E   T  L  Q ·
1701  TGAAGGAAAA AATGTTAAGG GCAGCCAGAG AGAAAGGTCG GGTTACCCTC AAAGGAAAGC CCATCAGACT AACAGCGGAT CTCTCGGCAG AAACCCTACA
                                                        Orf1
      ·A  R  R   E  W  G  P   I  F  N   I  L  K   E  K  N  F   Q  P  R   I  S  Y   P  A  K  L   S  F  I   S  E  G
1801  AGCCAGAAGA GAGTGGGGGC CAATATTCAA CATTCTTAAA GAAAAGAATT TTCAACCCAG AATTTCATAT CCAGCCAAAC TAAGCTTCAT AAGTGAAGGA
                                                        Orf1
       E  I  K  Y   F  I  D   K  Q  M   L  R  D  F   V  T  T   R  P  A   L  K  E  L   L  K  E   A  L  N   M  E  R  N ·
1901  GAAATAAAAT ACTTTATAGA CAAGCAAATG TTGAGAGATT TTGTCACCAC CAGGCCTGCC CTAAAAGAGC TCCTGAAGGA AGCGCTAAAC ATGGAAAGGA
                        Orf1
      · Y  R  Y   Q  P  L   Q  N  H  A   K  M
2001  ACTACCGGTA CCAGCCGCTG CAAAATCATG CCAAAATGTA AAGACCATCG AGACTAGGAA GAAACTGCAT CAACTAATGA GCAAAATCAC CAGCTAACAT
                                                          Orf2
         M  T   G  S  N  S   H  I  T   I  L  T   L  N  I  N   G  L  N   S  A  I   K  R  H  R   L  A  S   W  I  K
2101  CATAATGACA GGATCAAATT CACACATAAC AATATTAACT TTAAATATAA ATGGACTAAA TTCTGCAATT AAAAGACACA GACTGGCAAG TTGGATAAAG
                                                        Orf2
       S  Q  D  P   S  V  C   C  I  Q   E  T  H  L   T  C  R   D  T  H   R  L  K  I   K  G  W   R  K  I   Y  Q  A  N ·
2201  AGTCAAGACC CATCAGTGTG CTGTATTCAG GAAACCCATC TCACGTGCAG AGACACACAT AGGCTCAAAA TAAAAGGATG GAGGAAGATC TACCAAGCCA
                                                        Orf2
      · G  K  Q   K  K  A   G  V  A  I   L  V  S   D  K  T   D  F  K  P   T  K  I   K  R  D   K  E  G  H   Y  I  M ·
2301  ATGGAAAACA AAAAAAGGCA GGGGTTGCAA TCCTAGTCTC TGATAAAACA GACTTTAAAC CAACAAAGAT CAAAAGAGAC AAAGAAGGCC ATTACATAAT
                                                        Orf2
      ·V  K  G   S  I  Q  Q   E  E  L   T  I  L   N  I  Y  A   P  N  T   G  A  P   R  F  I  K   Q  V  L   S  D  L
2401  GGTAAAGGGA TCAATTCAAC AAGAGGAGCT AACTATCCTA AATATTTATG CACCCAATAC AGGAGCACCC AGATTCATAA AGCAAGTCCT CAGTGACCTA
                                                        Orf2
       Q  R  D  L   D  S  H   T  L  I   M  G  D  F   N  T  P   L  S  T   L  D  R  S   T  R  Q   K  V  N   K  D  T  Q ·
2501  CAAAGAGACT TAGACTCCCA CACATTAATA ATGGGAGACT TTAACACCCC ACTGTCAACA TTAGACAGAT CAACRAGACA GAAAGTCAAC AAGGATACCC
                                                        Orf2
      · E  L  N   S  A  L   H  Q  A  D   L  I  D   I  Y  R   T  L  H  P   K  S  T   E  Y  T   F  F  S  A   P  H  H ·
2601  AGGAATTGAA CTCAGCTCTG CACCAAGCAG ACCTAATAGA CATCTACAGA ACTCTCCACC CCAAATCAAC AGAATATACA TTTTTTTCAG CACCACACCA
                                                        Orf2
      ·T  Y  S   K  I  D  H   I  V  G   S  K  A   L  L  S  K   C  K  R   T  E  I   I  T  N  Y   L  S  D   H  S  A
2701  CACCTATTCC AAAATTGACC ACATAGTTGG AAGTAAAGCT CTCCTCAGCA AATGTAAAAG AACAGAAATT ATAACAAACT ATCTCTCAGA CCACAGTGCA
                                                        Orf2
       I  K  L  E   L  R  I   K  N  L   T  Q  S  R   A  T  T   W  K  L   N  N  L  L   L  N  D   Y  W  V   H  N  E  M ·
```

```
2801  ATCAAACTAG AACTCAGGAT TAAGAATCTC ACTCAAAGCC GCGCAACTAC ATGGAAACTG AACAACCTGC TCCTGAATGA CTACTGGGTA CATAACGAAA
                                                  Orf2
      · K A E  I K M  F F E  T N E  N K D  T T Y  Q N L  W D A  F K A  V C R  G K F ·
2901  TGAAGGCAGA AATAAAGATG TTCTTTGAAA CCAACGAGAA CAAAGACACC ACATACCAGA ATCTCTGGGA CGCATTCAAA GCAGTGTGTA GAGGGAAATT
                                                  Orf2
      ·I A L  N A Y  Q R K  Q E R  S K I  D T L  T S Q  L K E  L E K  Q E Q  T H S
3001  TATAGCACTA AATGCCTACC AGAGAAAGCA GGAAAGATCC AAAATTGACA CCCTAACATC ACAATTAAAA GAACTAGAAA AGCAAGAGCA AACACATTCA
                                                  Orf2
       K A S R  R Q E  I T K  I R A E  L K E  I E T  Q K T L  Q K I  N E S  R S W F ·
3101  AAAGCTAGCA GAAGGCAAGA AATAACTAAA ATCAGAGCAG AACTGAAGGA AATAGAGACA CAAAAAACCC TTCAAAAAAT CAATGAATCC AGGAGCTGGT
                                                  Orf2
      · F E R  I N K  I D R P  L A R  L I K  K K R E  K N Q  I D T  I K N D  K G D ·
3201  TTTTTGAAAG GATCAACAAA ATTGATAGAC CGCTAGCAAG ACTAATAAAG AAAAAAAGAG AGAAGAATCA AATAGACACA ATAAAAAATG ATAAAGGGGA
                                                  Orf2
      ·I T T  D P T E  I Q T  T I R  E Y Y K  H L Y  A N K  L E N L  E E M  D T F
3301  TATCACCACC GATCCCACAG AAATACAAAC TACCATCAGA GAATACTACA AACACCTCTA CGCAAATAAA CTAGAAAATC TGGAAGAAAT GGATACATTC
                                                  Orf2
       L D T Y  T L P  R L N  Q E E V  E S L  N R P  I T G S  E I V  A I I  N S L P ·
3401  CTCGACACAT ACACTCTCCC AAGACTAAAC CAGGAAGAAG TTGAATCTCT GAATCGACCA ATAACAGGCT CTGAAATTGT GGCAATAATC AATAGTTTAC
                                                  Orf2
      · T K K  S P G  P D G F  T A E  F Y Q  R Y K  E E L V  P F L  L K L  F Q S I ·
3501  CAACCAAAAA GAGTCCAGGA CCAGATGGAT TCACAGCCGA ATTCTACCAG AGGTACAAGG AGGAACTGGT ACCATTCCTT CTGAAACTAT TCCAATCAAT
                                                  Orf2
      ·E K E  G I L P  N S F  Y E A  S I I L  I P K  P G R  D T T K  K E N  F R P
3601  AGAAAAAGAG GGAATCCTCC CTAACTCATT TTATGAGGCC AGCATCATTC TGATACCAAA GCCGGGCAGA GACACAACCA AAAAAGAGAA TTTTAGACCA
                                                  Orf2
       I S L M  N I D  A K I  L N K I  L A N  R I Q  Q H I K  K L I  H H D  Q V G F ·
3701  ATATCCTTGA TGAACATTGA TGCAAAAATC CTCAATAAAA TACTGGCAAA CCGAATCCAG CAGCACATCA AAAAGCTTAT CCACCATGAT CAAGTGGGCT
                                                  Orf2
      · I P G  M Q G  W F N I  R K S  I N V  I Q H  I N R A  K G K  N H M  I I S I ·
3801  TCATCCCTGG GATGCAAGGC TGGTTCAATA TACGCAAATC AATAAATGTA ATCCAGCATA TAAACAGAGC CAAAGRCAAA AACCACATGA TTATCTCAAT
                                                  Orf2
      ·D A E  K A F D  K I Q  Q P F  M L K T  L N K  L G I  D G T Y  F K I  I R A
3901  AGATGCAGAA AAAGCCTTTG ACAAAATTCA ACAACCCTTC ATGCTAAAAA CTCTCAATAA ATTAGGTATT GATGGGACGT ATTTCAAAAT AATAAGAGCT
                                                  Orf2
       I Y D K  P T A  N I I  L N G Q  K L E  A F P  L K T G  T R Q  G C P  L S P L ·
4001  ATCTATGACA AACCCACAGC CAATATCATA CTGAATGGGC AAAAACTGGA AGCATTCCCT TTGAAAACTG GCACAAGACA GGGATGCCCT CTCTCACCGC
                                                  Orf2
      · L F N  I V L  E V L A  R A I  R Q E  K E I K  G I Q  L G K  E E V K  L S L ·
4101  TCCTATTCAA CATAGTGTTG GAAGTTCTGG CCAGGGCAAT CAGGCAGGAG AAGGAAATAA AGGGTATTCA ATTAGGAAAA GAGGAAGTCA AATTGTCCCT
                                                  Orf2
      ·F A D  D M I V  Y L E  N P I  V S A Q  N L L  K L I  S N F S  K V S  G Y K
4201  GTTTGCAGAC GACATGATTG TTTATCTAGA AAACCCCATC GTCTCAGCCC AAAATCTCCT TAAGCTGATA AGCAACTTCA GCAAAGTCTC AGGATACAAA
                                                  Orf2
       I N V Q  K S Q  A F L  Y T N N  R Q T  E S Q  I M S E  L P F  T I A  S K R I ·
4301  ATCAATGTAC AAAAATCACA AGCATTCTTA TACACCAACA ACAGACAAAC AGAGAGCCAA ATCATGAGTG AACTCCCATT CACAATTGCT TCAAAGAGAA
                                                  Orf2
      · K Y L  G I Q  L T R D  V K D  L F K  E N Y K  P L L  K E I  K E E T  N K W ·
4401  TAAAATACCT AGGAATCCAA CTTACAAGGG ATGTGAAGGA CCTCTTCAAG GAGAACTACA AACCACTGCT CAAGGAAATA AAAGAGGAGA CAAACAAATG
                                                  Orf2
      ·K N I  P C S W  V G R  I N I  V K M A  I L P  K V I  Y R F N  A I P  I K L
4501  GAAGAACATT CCATGCTCAT GGGTAGGAAG AATCAATATC GTGAAAATGG CCATACTGCC CAAGGTAATT TACAGATTCA ATGCCATCCC CATCAAGCTA
                                                  Orf2
       P M T F  F T E  L E K  T T L K  F I W  N Q K  R A R I  A K S  I L S  Q K N K ·
4601  CCAATGACTT TCTTCACAGA ATTGGAAAAA ACTACTTTAA AGTTCATATG GAACCAAAAA AGAGCCCGCA TTGCCAAGTC AATCCTAAGC CAAAAGAACA
                                                  Orf2
      · A G G  I T L  P D F K  L Y Y  K A T  V T K T  A W Y  W Y Q  N R D I  D Q W ·
4701  AAGCTGGAGG CATCACACTA CCTGACTTCA AACTATACTA CAAGGCTACA GTAACCAAAA CAGCATGGTA CTGGTACCAA AACAGAGATA TAGATCAATG
                                                  Orf2
      ·N I T  E P S E  I M P  H I Y  N Y L I  F D K  P E K  N Q Q W  G K D  S L F
4801  GAACATAACA GAGCCCTCAG AAATAATGCC GCATATCTAC AACTATCTGA TCTTTGACAA ACCTGAGAAA AACCAGCAAT GGGGAAAGGA TTCCCTGTTT
                                                  Orf2
       N K W C  W E N  W L A  I C R K  L K L  D P F  L T P Y  T K I  N S R  W I K D ·
4901  AATAAATGGT GCTGGGAAAA CTGGCTAGCC ATATGTAGAA AGCTGAAACT GGATCCCTTC CTTACACCTT ATACAAAAAT CAATTCAAGA TGGATTAAAG
                                                  Orf2
```

```
     · L N V  K P K  T I K T  L E E  N L G  I T I Q  D I G  V G K  D F M S  K T P ·
5001 ATTTAAACGT TAAACCTAAA ACCATAAAAA CCCTAGAAGA AAACCTAGGC ATTACCATTC AGGACATAGG CGTGGGCAAG GACTTCATGT CCAAAACACC
                                              Orf2
     ·K A M  A T K D  K I D  K W D  L I K L  K S F  C T A  K E T T  I R V  N R Q
5101 AAAAGCAATG GCAACAAAAG ACAAAATTGA CAAATGGGAT CTAATTAAAC TAAAGAGCTT CTGCACAGCA AAAGAAACTA CCATCAGAGT GAACAGGCAA
                                              Orf2
      P T T W  E K I  F A T  Y S S D  K G L  I S R  I Y N E  L K Q  I Y K  K K T N ·
5201 CCTACAACAT GGGAGAAAAT TTTTGCAACC TACTCATCTG ACAAAGGGCT AATATCCAGA ATCTACAATG AACTYAAACA AATTTACAAG AAAAAAACAA
                                              Orf2
     · N P I  K K W  A K D M  N R H  F S K  E D I Y  A A K  K H M  K K C S  S S L ·
5301 ACAACCCCAT CAAAAAGTGG GCGAAGGACA TGAACAGACA CTTCTCAAAA GAAGACATTT ATGCAGCCAA AAAACACATG AAGAAATGCT CATCATCACT
                                              Orf2
     ·A I R  E M Q I  K T T  M R Y  H L T P  V R M  A I I  K K S G  N N R  C W R
5401 GGCCATCAGA GAAATGCAAA TCAAAACCAC TATGAGATAT CATCTCACAC CAGTTAGAAT GGCAATCATT AAAAAGTCAG GAAACAACAG GTGCTGGAGA
                                              Orf2
      G C G E  I G T  L L H  C W W D  C K L  V Q P  L W K S  V W R  F L R  D L E L ·
5501 GGATGCGGAG AAATAGGAAC ACTTTTACAC TGTTGGTGGG ACTGTAAACT AGTTCAACCA TTGTGGAAGT CAGTGTGGCG ATTCCTCAGG GATCTAGAAC
                                              Orf2
     · E I P  F D P  A I P L  L G I  Y P N  E Y K S  C C Y  K D T  C T R M  F I A ·
5601 TAGAAATACC ATTTGACCCA GCCATCCCAT TACTGGGTAT ATACCCAAAT GAGTATAAAT CATGCTGCTA TAAAGACACA TGCACACGTA TGTTTATTGC
                                              Orf2
     ·A L F  T I A K  T W N  Q P K  C P T M  I D W  I K K  M W H I  Y T M  E Y Y
5701 GGCACTATTC ACAATAGCAA AGACTTGGAA CCAACCCAAA TGTCCAACAA TGATAGACTG GATTAAGAAA ATGTGGCACA TATACACCAT GGAATACTAT
                                              Orf2
      A A I K  N D E  F I S  F V G T  W M K  L E T  I I L S  K L S  Q E Q  K T K H ·
5801 GCAGCCATAA AAAATGATGA GTTCATATCC TTTGTAGGGA CATGGATGAA ATTGGAAACC ATCATTCTCA GTAAACTATC GCAAGAACAA AAAACCAAAC
                 Orf2
     · R I F  S L I  G G N
5901 ACCGCATATT CTCACTCATA GGTGGGAATT GAACAATGAG ATCACATGGA CACAGGAAGG GGAATATCAC ACTCTGGGGA CTGTGGTGGG GTCGGGGGAG
6001 GGGGGAGGGA TAGCATTGGG AGATATACCT AATGCTAGAT GACACATTAG TGGGTGCAGC GCACCAGCAT GGCACATGTA TACATATGTA ACTAACCTGC
6101 ACAATGTGCA CATGTACCCT AAAACTTAGA GTATAATAAA AAAAAAAAAA AAAAAAAAAA AAAAAAAAAA AAAAAAAAAA AAAAAAAAAA AAAAAAAAAA
            3'TSD
         ~~~~~~~~~~~~~
6201 AAAAACTATA CTGAATGCTA TCTTTCATAT TTCATGTTGG CAAAACATGA AAAATCTCAC ATAATTTTGC TTTTATGAAG CAAAGCTTTT ATGAAGCAAA
6301 CCTTTTATGA AGCAAAACTG AATAATGCAA GATACAAACG AAGGCTTATT TGTTTAAAAA TGAATAAAAA GAAATATATA TTCTAACAGA CTTTGTTGAG
6401 TCTTTAGGTT TTTCTAAATA TAAGATTATG TCCTCTGTGA ACAAGAATAA TTGGACTTAT TCCAAAGTTG CAGG
```

L1-dn14

```
   1 AGGTAAATGT TGGAAACATT TGTGAACAGA AGCAGGAAAC CTCATTAAAG TAAGGTATAA AAAGAACTTG AATAAATACA TAAACAACTT GAGCCTGTAA
                                                                   TSD
                                                          ~~~~~~~~~~~~~~~~~~
 101 TACAGGGCAA TAGAAAAACA CCAAAAAATG TCCAAATTGT CCATTTATGA TGAAAATACA CAAGCACAGG GGGAGGAGCC AAGATGGCCG AATAGGAACA
 201 GCTCCGGTCT ACAGCTCCCA GCGTGAGCGA CGCAGAAGAC GGTGATTTCT GCATTTCCAT CTGAGGTACC GGGTTCATCT CACTAGGGAG TGCCAGACAG
 301 TGGGCGCAGG CCAGTGTGTG TGCGCACCGT GCGCGAGCCG AAGCAGGGCG ACGCATTGCC TCACCTGGGA AGCGCAACGG GTCAGGGAGT TCCCTTTCCG
 401 AGTCAAAGAA AGGGGTGACG GACGCACCTG GAAAATCGGG TCACTCCCAC CCGAATATTG CGCTTTTCAG ACCGGCTTAA GAAACGGCGC ACCACGAGAC
 501 TATATCCCAC ACCTGGCTCG GAGGGTCCTA CGCCCACGGA ATCTCGCTGA TTGCTAGCAC AGCAGTCTGA GATCAAACTG CAAGGCGGCA ACGAGGCTGG
 601 GGGAGGGGCG CCCGCCATTG CCCAGGCTTG CTTAGGTAAA CAAAGCAGCC GGGAAGCTCG AACTGGGTGG AGCCCACCAC AGCTCAAGGA GGCCTGCCTG
 701 CCTCTGTAGG CTCCACCTCT GGGGGCAGGG CACAGACAAA CAAAAAGACA GCAGTAACCT CTGCAGACTT AAGTGTCCCT GTCTGACAGC TTTGAAGAGA
 801 GCAGTGGTTC TCCCAGCACG CAGCTGGAGA TCTGAGAACG GGCAGACTGC CTCCTCAAGT GGGTCCCTGA CTCCTGACCC CCGAGCAGCC TAACTGGGAG
 901 GCACCCCCCA GCAGGGGCAC ACTGACACCT CACATGGCAG GGTATTCCAA CAGACCTGCA GCTGAGGGTC CTGTCTGTTA GAAGCAAAAC TAACAACCAG

                                                                                                 ORF1
                                                                                        M G K  K Q N  R K T
1001 AAAGGACATC TACACCGAAA ACCCATCTGT ACATCACCAT CATCAAAGAC CAAAAGTAGA TAAAACCACA AAGATGGGGA AAAAACAGAA CAGAAAAACT

                                              ORF1
      G N S K  T Q S  A S P  P P K E  R S S  S P A  T E Q S  W M E  N D F  D E L R ·
1101 GGAAACTCTA AAACGCAGAG CGCCTCTCCT CCTCCAAAGG AACGCAGTTC CTCACCAGCA ACAGAACAAA GCTGGATGGA GAATGATTTT GATGAGCTGA

                                              ORF1
     · E E G  F R R  S N Y S  E L R  E D I  Q T K G  K E V  E N F  E K N L  E E C ·
1201 GAGAAGAAGG CTTCAGACGA TCAAATTACT CTGAGCTACG GGAGGACATT CAAACCAAAG GCAAAGAAGT TGAAAACTTT GAAAAAAATT TAGAAGAATG

                                              ORF1
     ·I T R  I T N T  E K C  L K E  L M E L  K T K  A G E  L R E E  C R S  L R S
1301 TATAACTAGA ATAACCAATA CAGAGAAGTG CTTAAAGGAG CTGATGGAGC TGAAAACCAA GGCTGGAGAA CTACGTGAAG AATGCAGAAG CCTCAGGAGC

                                              ORF1
      R C D Q  L E E  R V S  A M E D  E M N  E M K  R E G K  F R E  K R I  K R N E ·
1401 CGATGCGATC AACTGGAAGA AAGGGTATCA GCAATGGAAG ATGAAATGAA TGAAATGAAG CGAGAAGGGA AGTTTAGAGA AAAAAGAATA AAAAGAAATG

                                              ORF1
     · Q S L  Q E I  W D Y V  K R P  N L R  L I G V  P E S  D V E  N G T K  L E N ·
```

```
1501 AGCAAAGCCT CCAAGAAATA TGGGACTATG TGAAAAGACC AAATCTACGT CTGATTGGTG TACCTGAAAG TGATGTGGAG AATGGAACCA AGTTGGAAAA

                                                  ORF1
     ·T L Q  D I I Q  E N F  P N L  A R Q A  N V Q  I Q E  I Q R T  P Q R  Y S S
1601 CACTCTGCAG GATATTATCC AGGAGAACTT CCCCAATCTA GCAAGGCAGG CCAACGTTCA GATTCAGGAA ATACAGAGAA CGCCACAAAG ATACTCCTCG

                                                  ORF1
      R R A T  P R H  I I V  R F T K  V E M  K E K  M L R A  A R E  K G R  V T L K·
1701 AGAAGAGCAA CTCCAAGACA CATAATTGTC AGATTCACCA AAGTTGAAAT GAAGGAAAAA ATGTTAAGGG CAGCCAGAGA GAAAGGTCGG GTTACCCTCA

                                                  ORF1
     · G K P  I R L  T A D L  S A E  T L Q  A R R E  W G P  I F N  I L K E  K N F·
1801 AAGGAAAGCC CATCAGACTA ACAGCGGATC TCTCGGCAGA AACCCTACAA GCCAGAAGAG AGTGGGGGCC AATATTCAAC ATTCTTAAAG AAAAGAATTT

                                                  ORF1
     ·Q P R  I S Y P  A K L  S F I  S E G E  I K Y  F I D  K Q M L  R D F  V T T
1901 TCAACCCAGA ATTTCATATC CAGCCAAACT AAGCTTCATA AGTGAAGGAG AAATAAAATA CTTTATAGAC AAGCAAATGT TGAGAGATTT TGTCACCACC

                                             ORF1
      R P A L  K E L  L K E  A L N M  E R N  N R Y  Q P L Q  N H A  K M
2001 AGGCCTGCCC TAAAAGAGCT CCTGAAGGAA GCGCTAAACA TGGAAAGGAA CAACCGGTAC CAGCCGCTGC AAAATCATGC CAAAATGTAA AGACCATCGA

                                                                        ORF2
                                                               M T G  S T S  H I T  I L T L  N I N·
2101 GACTAGGAAG AAACTGCATC AACTAATGAG CAAAATCACC AGCTAACATC ATAATGACAG GATCAACTTC ACACATAACA ATATTAACTT TAAATATAAA

                                                  ORF2
     ·G L N  S A I K  R H R  L A S  W I K S  Q D P  S V C  C I Q E  T H L  T C R
2201 TGGACTAAAT TCTGCAATTA AAAGACACAG ACTGGCAAGT TGGATAAAGA GTCAAGACCC ATCAGTGTGC TGTATTCAGG AAACCCATCT CACGTGCAGA

                                                  ORF2
      D T H R  L K I  K G W  R K I Y  Q A N  G K Q  K K A G  V A I  L V S  D K T D·
2301 GACACACATA GGCTCAAAAT AAAAGGATGG AGGAAGATCT ACCAAGCCAA TGGAAAACAA AAAAAGGCAG GGGTTGCAAT CCTAGTCTCT GATAAAACAG

                                                  ORF2
     · F K P  T K I  K R D K  E G H  Y I M  V K G S  I Q Q  E E L  T I L N  I Y A·
2401 ACTTTAAACC AACAAAGATC AAAAGAGACA AAGAAGGCCA TTACATAATG GTAAAGGGAT CAATTCAACA AGAGGAGCTA ACAATCCTAA ATATTTATGC

                                                  ORF2
     ·P N T  G A P R  F I K  Q V L  S D L Q  R D L  D S H  T L I M  G D F  N T P
2501 ACCCAATACA GGAGCACCCA GATTCATAAA GCAAGTCCTC AGTGACCTAC AAAGAGACTT AGACTCCCAC ACATTAATAA TGGGAGACTT TAACACCCCA

                                                  ORF2
      L S T L  D R S  T R Q  K V N K  D T Q  E L N  S A L H  Q A D  L I D  I Y R T·
2601 CTGTCAACAT TAGACAGATC AACGAGACAG AAAGTTAACA AGGATACCCA GGAATTGAAC TCAGCTCTGC ACCAAGCAGA CCTAATAGAC ATCTACAGAA

                                                  ORF2
     · L H P  K S T  E Y T F  F S A  P H H  T Y S K  I D H  I V G  S K A L  L S K·
2701 CTCTCCACCC CAAATCAACA GAATATACAT TTTTTTCAGC ACCACACCAC ACCTATTCCA AAATTGACCA CATAGTTGGA AGTAAAGCTC TCCTCAGCAA

                                                  ORF2
     ·C K R  T E I I  T N Y  L S D  H S A I  K L E  L R I  K N L T  Q S R  S T T
2801 ATGTAAAAGA ACAGAAATTA TAACAAACTA TCTCTCAGAC CACAGTGCAA TCAAACTAGA ACTCAGGATT AAGAATCTCA CTCAAAGCCG CTCAACTACA

                                                  ORF2
      W K L N  N L L  L N D  Y W V H  N E M  K A E  I K M F  F E T  N E N  K D T T·
2901 TGGAAACTGA ACAACCTGCT CCTGAATGAC TACTGGGTAC ATAACGAAAT GAAGGCAGAA ATAAAGATGT TCTTTGAAAC CAACGAGAAC AAAGACACCA

                                                  ORF2
     · Y Q N  L W D  A F K A  V C R  G K F  I A L N  A Y K  R K Q  E R S K  I D T·
3001 CATACCAGAA TCTCTGGGAC GCATTCAAAG CAGTGTGTAG AGGGAAATTT ATAGCACTAA ATGCCTACAA GAGAAAGCAG GAAAGATCCA AAATTGACAC

                                                  ORF2
     ·L T S  Q L K E  L E K  Q E Q  T H S K  A S R  R Q E  I T K I  R A E  L K E
3101 CCTAACATCA CAATTAAAAG AACTAGAAAA GCAAGAGCAA ACACATTCAA AAGCTAGCAG AAGGCAAGAA ATAACTAAAA TCAGAGCAGA ACTGAAGGAA

                                                  ORF2
      I E T Q  K T L  Q K I  N E S R  S W F  F E R  I N K I  D R P  L A R  L I K K·
3201 ATAGAGACAC AAAAAACCCT TCAAAAAATC AATGAATCCA GGAGCTGGTT TTTTGAAAGG ATCAACAAAA TTGATAGACC GCTAGCAAGA CTAATAAAGA

                                                  ORF2
```

```
     · K R E  K N Q  I D T I  K N D  K G D  I T T D  P T E  I Q T  T I R E  Y Y K ·
     --------------------------------------------------------------------------------
3301 AAAAAAGAGA GAAGAATCAA ATAGACACAA TAAAAAATGA TAAAGGGGAT ATCACCACCG ATCCCACAGA AATACAAACT ACCATCAGAG AATACTACAA
                                                ORF2
     --------------------------------------------------------------------------------
     ·H L Y  A N K L  E N L  E E M  D T F L  D T Y  T L P  R L N Q  E E V  E S L
     --------------------------------------------------------------------------------
3401 ACACCTCTAC GCAAATAAAC TAGAAAATCT AGAAGAAATG GATACATTCC TCGACACATA CACTCTCCCA AGACTAAACC AGGAAGAAGT TGAATCTCTG
                                                ORF2
     --------------------------------------------------------------------------------
      N R P I  T G S  E I V  A I I N  S L P  T K K  S P G P  D G F  T A E  F Y Q R ·
     --------------------------------------------------------------------------------
3501 AATCGACCAA TAACAGGCTC TGAAATTGTG GCAATAATCA ATAGTTTACC AACCAAAAAG AGTCCAGGAC CAGATGGATT CACAGCCGAA TTCTACCAGA
                                                ORF2
     --------------------------------------------------------------------------------
     · Y K E  E L V  P F L L  K L F  Q S I  E K E G  I L P  N S F  Y E A S  I I L ·
     --------------------------------------------------------------------------------
3601 GGTACAAGGA GGAACTGGTA CCATTCCTTC TGAAACTATT CCAATCAATA GAAAAAGAGG GAATCCTCCC TAACTCATTT TATGAGGCCA GCATCATTCT
                                                ORF2
     --------------------------------------------------------------------------------
     ·I P K  P G R D  T T K  K E N  F R P I  S L M  N I D  A K I L  N K I  L A N
     --------------------------------------------------------------------------------
3701 GATACCAAAG CCGGGCAGAG ACACAACCAA AAAAGAGAAT TTTAGACCAA TATCCTTGAT GAACATTGAT GCAAAAATCC TCAATAAAAT ACTGGCAAAC
                                                ORF2
     --------------------------------------------------------------------------------
      R I Q Q  H I K  K L I  H H D Q  V G F  I P G  M Q G W  F N I  R K S  I N V I ·
     --------------------------------------------------------------------------------
3801 CGAATCCAGC AGCACATCAA AAAGCTTATC CACCATGATC AAGTGGGCTT CATCCCTGGG ATGCAAGGCT GGTTCAATAT ACGCAAATCA ATAAATGTAA
                                                ORF2
     --------------------------------------------------------------------------------
     · Q H I  N R A  K D K N  H M I  I S I  D A E K  A F D  K I Q  Q P F M  L K T ·
     --------------------------------------------------------------------------------
3901 TCCAGCATAT AAACAGAGCC AAAGACAAAA ACCACATGAT TATCTCAATA GATGCAGAAA AAGCCTTTGA CAAAATTCAA CAACCCTTCA TGCTAAAAAC
                                                ORF2
     --------------------------------------------------------------------------------
     ·L N K  L G I D  G T Y  F K I  I R A I  Y D K  P T A  N I I L  N G Q  K L E
     --------------------------------------------------------------------------------
4001 TCTCAATAAA TTAGGTATTG ATGGGACGTA TTTCAAAATA ATAAGAGCTA TCTATGACAA ACCCACAGCC AATATCATAC TGAATGGGCA AAAACTGGAA
                                                ORF2
     --------------------------------------------------------------------------------
      A F P L  K T G  T R Q  G C P L  S P L  L F N  I V L E  V L A  R A I  R Q E K ·
     --------------------------------------------------------------------------------
4101 GCATTCCCTT TGAAAACCGG CACAAGACAG GGATGCCCTC TCTCACCACT CCTATTCAAC ATAGTGTTGG AAGTTCTGGC CAGGGCAATC AGGCAGGAGA
                                                ORF2
     --------------------------------------------------------------------------------
     · E I K  G I Q  L G K E  E V K  L S L  F A D D  M I V  Y L E  N P I V  S A Q ·
     --------------------------------------------------------------------------------
4201 AGGAAATAAA GGGTATTCAA TTAGGAAAAG AGGAAGTCAA ATTGTCCCTG TTTGCAGACG ACATGATTGT TTATCTAGAA AACCCCATCG TCTCAGCCCA
                                                ORF2
     --------------------------------------------------------------------------------
     ·N L L  K L I S  N F S  K V S  G Y K I  N V Q  K S Q  A F L Y  T N N  R Q T
     --------------------------------------------------------------------------------
4301 AAATCTCCTT AAGCTGATAA GCAACTTCAG CAAAGTCTCA GGATACAAAA TCAATGTACA AAAATCACAA GCATTCTTAT ACACCAACAA CAGACAAACA
                                                ORF2
     --------------------------------------------------------------------------------
      E S Q I  M G E  L P F  T I A S  K R I  K Y L  G I Q L  T R D  V K D  L F K E ·
     --------------------------------------------------------------------------------
4401 GAGAGCCAAA TCATGGGTGA ACTCCCATTC ACAATTGCTT CAAAGAGAAT AAAATACCTA GGAATCCAAC TTACAAGGGA TGTGAAGGAC CTCTTCAAGG
                                                ORF2
     --------------------------------------------------------------------------------
     · N Y K  P L L  K E I K  E E T  N K W  K N I P  C S W  V G R  I N I V  K M A ·
     --------------------------------------------------------------------------------
4501 AGAACTACAA ACCACTGCTC AAGGAAATAA AAGAGGAGAC AAACAAATGG AAGAACATTC CATGCTCATG GGTAGGAAGA ATCAATATCG TGAAAATGGC
                                                ORF2
     --------------------------------------------------------------------------------
     ·I L P  K V I Y  R F N  A I P  I K L P  M T F  F T E  L E K T  T L K  F I W
     --------------------------------------------------------------------------------
4601 CATACTGCCC AAGGTAATTT ACAGATTCAA TGCCATCCCC ATCAAGCTAC CAATGACTTT CTTCACAGAA TTGGAAAAAA CTACTTTAAA GTTCATATGG
                                                ORF2
     --------------------------------------------------------------------------------
      N Q K R  A R I  A K S  I L S Q  K N K  A G G  I T L P  D F K  L Y Y  K A T V ·
     --------------------------------------------------------------------------------
4701 AACCAAAAAA GAGCCCGCAT TGCCAAGTCA ATCCTAAGCC AAAAGAACAA AGCTGGAGGC ATCACACTAC CTGACTTCAA ACTATACTAC AAGGCTACAG
                                                ORF2
     --------------------------------------------------------------------------------
     · T K T  A W Y  W Y Q N  R D I  D Q W  N R T E  P S E  I M P  H I Y N  Y L I ·
     --------------------------------------------------------------------------------
4801 TAACCAAAAC AGCATGGTAC TGGTACCAAA ACAGAGATAT AGATCAATGG AACAGAACAG AGCCCTCAGA AATAATGCCG CATATCTACA ACTATCTGAT
                                                ORF2
     --------------------------------------------------------------------------------
     ·F D K  P E K N  K Q W  G K D  S L F N  K W C  W E N  W L A I  C R K  L K L
     --------------------------------------------------------------------------------
4901 CTTTGACAAA CCTGAGAAAA ACAAGCAATG GGGAAAGGAT TCCCTATTTA ATAAATGGTG CTGGGAAAAC TGGCTAGCCA TATGTAGAAA GCTGAAACTG
                                                ORF2
     --------------------------------------------------------------------------------
      D P F L  T P Y  T K I  N S R W  I K D  L N V  K P K T  I K T  L E E  N L G I ·
     --------------------------------------------------------------------------------
5001 GATCCCTTCC TTACACCTTA TACAAAAATC AATTCAAGAT GGATTAAAGA TTTAAACGTT AAACCTAAAA CCATAAAAAC CCTAGAAGAA AACCTAGGCA
                                                ORF2
     --------------------------------------------------------------------------------
     · T I Q  D I G  V G K D  F M S  K T P  K A M A  T K D  K I D  K W D L  I K L ·
```

```
5101 TTACCATTCA GGACATAGGC GTGGGCAAGG ACTTCATGTC CAAAACACCA AAAGCAATGG CAACAAAAGA CAAAATTGAC AAATGGGATC TAATTAAACT

                                          ORF2
     ·K  S  F  C  T  A  K  E  T  T  I  R  V  N  R  Q  P  T  T  W  E  K  I  F  A  T  Y  S  S  D  K  G  L
5201 AAAGAGCTTC TGCACAGCAA AAGAAACTAC CATCAGAGTG AACAGGCAAC CTACAACATG GGAGAAAATT TTTGCAACCT ACTCATCTGA CAAAGGGCTA

                                          ORF2
      I  S  R  I  Y  N  E  L  K  Q  I  Y  K  K  K  T  N  N  P  I  K  K  W  A  K  D  M  N  R  H  F  S  K  E·
5301 ATATCCAGAA TCTACAATGA ACTCAAACAA ATTTACAAGA AAAAAACAAA CAACCCCATC AAAAAGTGGG CGAAGGACAT GAACAGACAC TTCTCAAAAG

                                          ORF2
     · D  I  Y  A  A  K  K  H  M  K  K  C  S  S  S  L  A  I  R  E  M  Q  I  K  T  T  M  R  Y  H  L  T  P·
5401 AAGACATTTA TGCAGCCAAA AAACACATGA AGAAATGCTC ATCATCACTG GCCATCAGAG AAATGCAAAT CAAAACCACT ATGAGATATC ATCTCACACC

                                          ORF2
     ·V  R  M  A  I  I  K  K  S  G  N  N  R  C  W  R  G  C  G  E  I  G  T  L  L  H  C  W  W  D  C  K  L
5501 AGTTAGAATG GCAATCATTA AAAAGTCAGG AAACAACAGG TGCTGGAGAG GATGCGGAGA AATAGGAACA CTTTTACACT GTTGGTGGGA CTGTAAACTA

                                          ORF2
      V  Q  P  L  W  K  S  V  W  R  F  L  R  D  L  E  L  E  I  P  F  D  P  A  I  P  L  L  G  I  Y  P  N  E·
5601 GTTCAACCAT TGTGGAAGTC AGTGTGGCGA TTCCTCAGGG ATCTAGAACT AGAAATACCA TTTGACCCAG CCATCCCATT ACTGGGTATA TACCCAAATG

                                          ORF2
     · Y  K  S  C  C  Y  K  D  T  C  T  R  M  F  I  A  A  L  F  T  I  A  K  T  W  N  Q  P  K  C  P  T  M·
5701 AGTATAAATC ATGCTGCTAT AAAGACACAT GCACACGTAT GTTTATTGCG GCACTATTCA CAATAGCAAA GACTTGGAAC CAACCCAAAT GTCCAACAAT

                                          ORF2
     ·I  D  W  I  K  K  M  W  H  I  Y  T  M  E  Y  Y  A  A  I  K  N  D  E  F  I  S  F  V  G  T  W  M  K
5801 GATAGACTGG ATTAAGAAAA TGTGGCACAT ATACACCATG GAATACTATG CAGCCATAAA AAATGATGAG TTCATATCCT TTGTAGGGAC ATGGATGAAA

                                ORF2
      L  E  T  I  I  L  S  K  L  S  Q  E  Q  K  T  K  H  R  I  F  S  L  I  G  G  N
5901 TTGGAAACCA TCATTCTCAG TAAACTATCG CAAGAACAAA AAACCAAACA CCGCATATTC TCACTCATAG GTGGGAATTG AACAATGAGA TCACATGGAC
6001 ACAGGAAGGG GAATATCACA CTCTGGGGAC TGTGGTGGGG TCGGGGGAGG GGGGAGGGAT AGCATTGGGA GATATACCTA ATGTTAGATG ACACATTAGT
6101 GGGTGCAGCG CACCAGCATG GCACATGTAT ACATATGTAA CTAACCTGCA CAATGTGCAC ATGTACCCTA AAACTTAGAG TATAATAAAA AAAAAAAAAA
                          TSD
                    ------------------
6201 AAAAAAAAAA AAAAAAAGAA AATACACAAG CACAAACCAG AGATGTCTTT AAACATTTTT CCCTAAATTT TAGATAAGCA GCCATCTAAC TGATATGTAC
6301 ATACAAAA
```

Supplementary Figure 10: Nucleotide sequences of full-length L1 *de novo* insertions L1-dn4, L1-dn6 and L1-dn14 and their genomic flanks identified in hiPSCs. In the case of L1-dn6, two independent long-range PCRs were performed on genomic iPSC DNA. Resulting PCR products, L1-dn6-5.4 and L1-dn6-2.2, were subcloned and sequenced. Each full-length L1 element codes for intact ORF1 and ORF2 proteins. TSDs are highlighted in green. Aminoacid residues that differ from the L1.3 reference sequence (accession number L19088.1)[1] are highlighted in red.

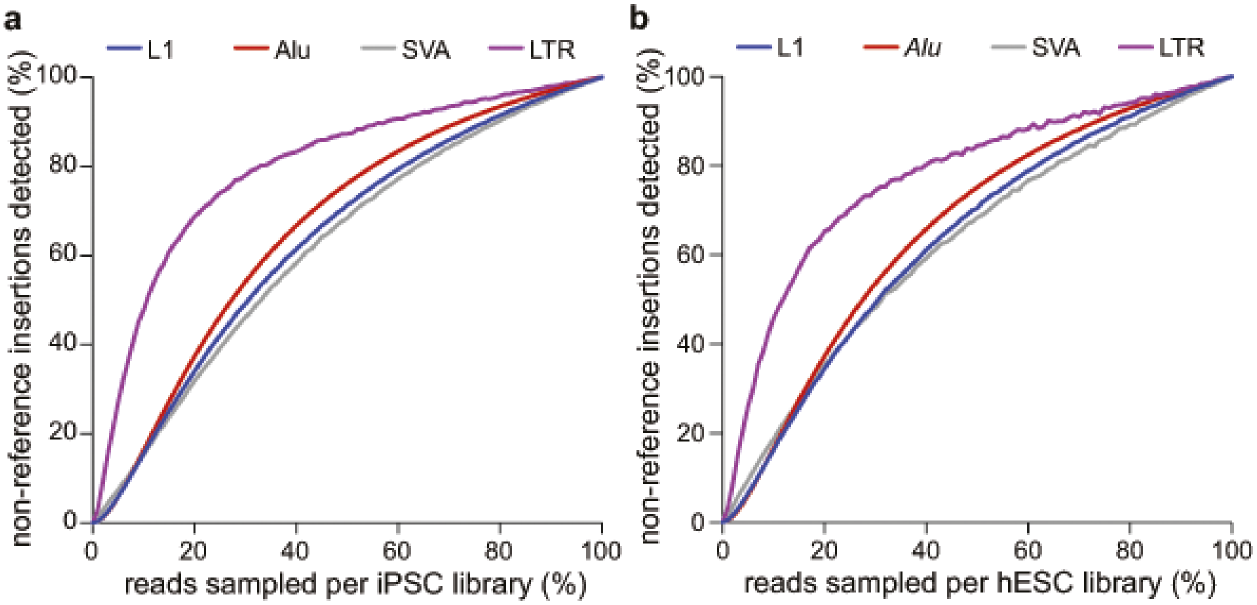

Supplementary Figure 11: Modeling the sensitivity of RC-seq for the detection of *de novo* insertions in pluripotent stem cell cultures. Presented are proportions of non-reference genome L1, *Alu*, SVA and LTR insertions detected as a function of sampling depth for hiPSC **(a)** and hESC **(b)** RC-seq libraries.

a

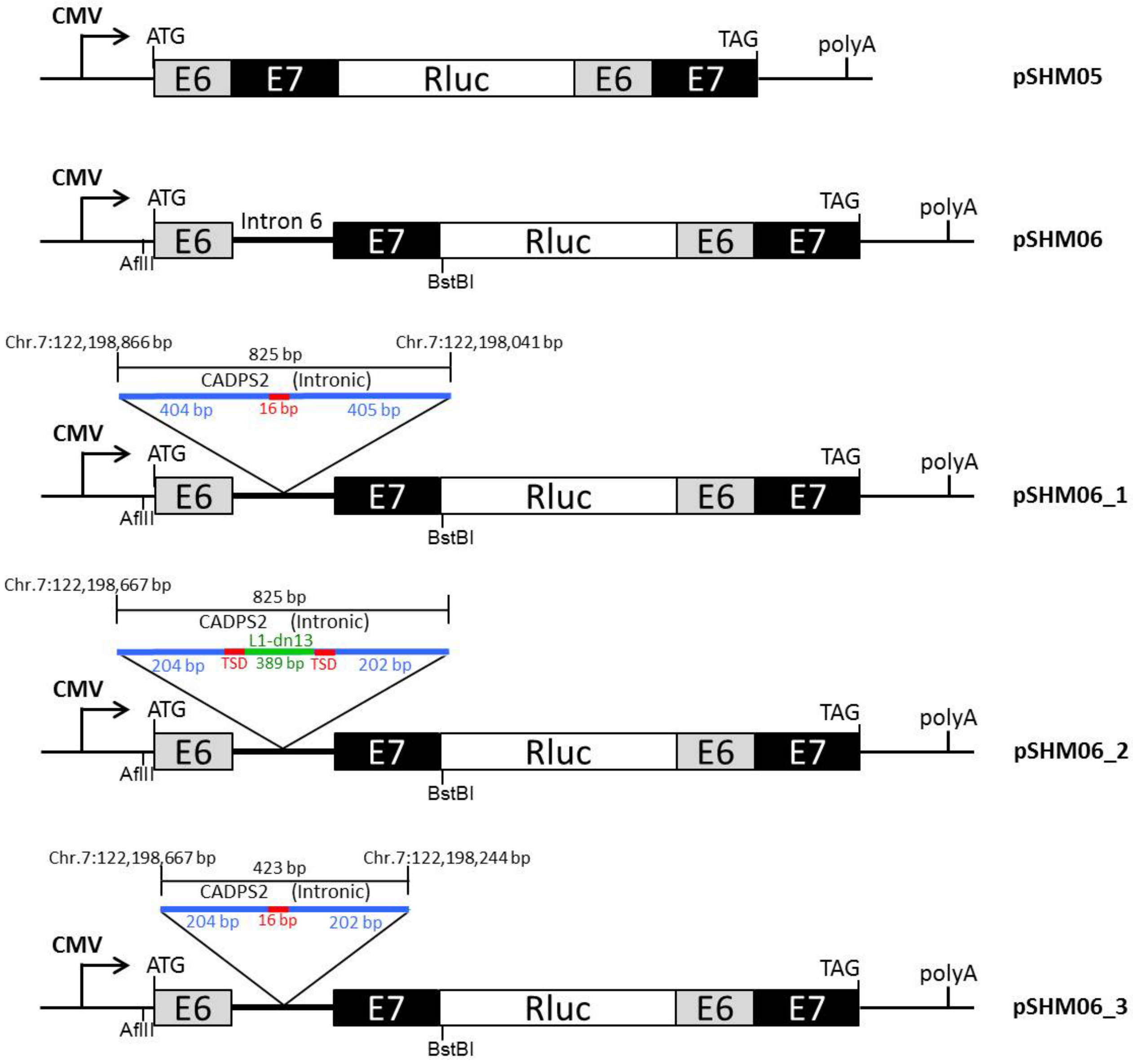
CMV
ATG
E6
E7
Rluc
E6
E7
TAG
polyA
pSHM05
CMV
ATG
Intron 6
E6
E7
Rluc
E6
E7
TAG
polyA
AflII
BstBI
pSHM06
Chr.7:122,198,866 bp
825 bp
Chr.7:122,198,041 bp
CADPS2 (Intronic)
404 bp
16 bp
405 bp
CMV
ATG
E6
E7
Rluc
E6
E7
TAG
polyA
AflII
BstBI
pSHM06_1
Chr.7:122,198,667 bp
825 bp
CADPS2 (Intronic)
L1-dn13
204 bp
TSD
389 bp
TSD
202 bp
CMV
ATG
E6
E7
Rluc
E6
E7
TAG
polyA
AflII
BstBI
pSHM06_2
Chr.7:122,198,667 bp
423 bp
Chr.7:122,198,244 bp
CADPS2 (Intronic)
204 bp
16 bp
202 bp
CMV
ATG
E6
E7
Rluc
E6
E7
TAG
polyA
AflII
BstBI
pSHM06_3

b

pSHM06_1: 1121-bp AflII/BstBI fragment

AflII

cttaagcttcagcgcctcggctccagccgccatggaagtacacgagaagctccgaggatggctgaagt
ccaacgtctctgatgcggtggctcagagcacccgtatcatttatgtaggtgagtggctttggttcccg
gctgaggtggagtgggctgaggactagactgagccctcggacatagtttggcctcaatcaggtatctc
agtagaaaagaaacaatgtagaagtgacagcttatatattttaagagaaagctacttctgaaccccat
atcactctaaacatttctgtgtgtaaatgtgtattttatatttcatttttttaagtcttagtttttcc
tcctgtatattaaatttgatggaatttttaacaaatcctaaaaatccattttgtggtttccattacac
cttagcaaagatgacaagtgacactgacattttattatgaagaagatacattagctttatgtttaaga
caagaaatcattttgtgccatgctgacaagacatttaaagtgttggtgacaaaaaataaatgagaaga
agcagaaatgcatacggaaaggttaagagaaacgaatggtaaagaacaaaatagtttgctaaataata
ttaaaagttatttgtacagagctatagtttaatttaaatgcgaatttaggatacagaaggaccttagt
aagcttttatatctgttaaatattttaattattatgactgtcaacatcagacacaatctctaaacatg
aactgagtcctattgaagtggaactgcttctaataaaaaaaaacaattcttcattatttacttttgaa
agatttgaaacaaaataccttgttgatatactagttaggatactgctaaatgctataacaagaaagt
cgttacatttcaataattcaaaataatatcaggtttgtaagagtttaccacaggtattcctgatagaa
gtgtgactttcttccacatggtgatttagggctctgatttctttcgtttcctaggaggtggggatggg
gcagactcatcccattcttgaccaagcccttgttctgctcccttcccaggctctgtgactggggcaac
ctgcaaggagctggccagcaccggtacttcgaa

BstBI

pSHM06_2: 1123-bp AflII/ BstBI fragment

AflII

Cttaagcttcagcgcctcggctccagccgccatggaagtacacgagaagctccgaggatggctgaagt
ccaacgtctctgatgcggtggctcagagcacccgtatcatttatgtaggtgagtggctttggttcccg
gctgaggtggagtgggctgaggactagactgagccctcggacataaaatccattttgtggtttccatt
acaccttagcaaagatgacaagtgacactgacattttattatgaagaagatacattagctttatgttt
aagacaagaaatcattttgtgccatgctgacaagacatttaaagtgttggtgacaaaaaataaatgag
aagaagcagaaatgcatacggaaaggttaagagaaacgaatggtaaagaacaaaatagttgatggttt
ccaatttcatccatgtccctacaaagatcattctcagtaaactatcgcaagaacaaaaaaccaaacac
cgcatattctcactcataggtgggaattgaacaatgagatcacatggacacaggaaggggaatatcac
actctggggactgtggtggggtcgggggagggggagggatagcattgggagatatacctaatgctag
atgacacattagtgggtgcagcgcaccagcatggcacatgtatacatatgtaactaacccgggcaatg
tgcacatgtaccctaaaacttaaagtataataaaaaaaaaaaaaaaaaaaaaaaaaaaaaaaaaaaaa
aaaaaaaaaaaaaaaaaaaaaaaaaaaaaaaaaaaaaaaaaaaagaacaaaatagtttgctaaataat
attaaaagttatttgtacagagctatagtttaatttaaatgcgaatttaggatacagaaggaccttag
taagcttttatatctgttaaatattttaattattatgactgtcaacatcagacacaatctctaaacat
gaactgagtcctattgaagtggaactgcttctaataaaaaaaaacaattcttcatggaggtggggatg
ggcagactcatcccattcttgaccaagcccttgttctgctcccttcccaggctctgtgactggggca
acctgcaaggagctggccagcaccggtacttcgaa

BstBI

pSHM06_3: 718-bp AflII/BstBI fragment

AflII

cttaagcttcagcgcctcggctccagccgccatggaagtacacgagaagctccgaggatggctgaagt
ccaacgtctctgatgcggtggctcagagcacccgtatcatttatgtaggtgagtggctttggttcccg
gctgaggtggagtgggctgaggactagactgagccctcggacataaaatccattttgtggtttccatt
acaccttagcaaagatgacaagtgacactgacattttattatgaagaagatacattagctttatgttt
aagacaagaaatcattttgtgccatgctgacaagacatttaaagtgttggtgacaaaaaataaatgag
aagaagcagaaatgcatacggaaaggttaagagaaacgaatggtaaagaacaaaatagtttgctaaat
aatattaaaagttatttgtacagagctatagtttaatttaaatgcgaatttaggatacagaaggacct
tagtaagcttttatatctgttaaatattttaattattatgactgtcaacatcagacacaatctctaaa
catgaactgagtcctattgaagtggaactgcttctaataaaaaaaaacaattcttcatggaggtgggg
atggggcagactcatcccattcttgaccaagcccttgttctgctcccttcccaggctctgtgactggg
gcaacctgcaaggagctggccagcaccggtacttcgaa

BstBI

c

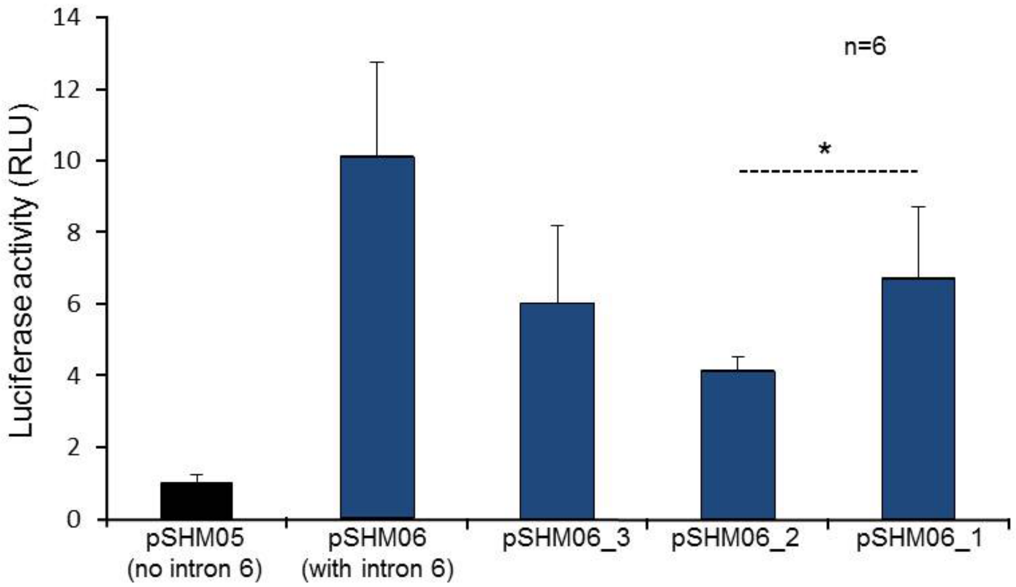

Supplementary Figure 12: The 389-bp L1-dn13 insertion flanked by *CADPS2* intron 7 sequences has the strongest inhibitory effect on luciferase reporter gene expression. **(a)** Schematic of TPI/Renilla luciferase reporter constructs used to evaluate the effect of the L1-dn13 insertion on reporter gene expression. . The 16-bp target sequence that was recognized by L1-EN and became the TSD of insertion L1-dn13, is marked in red. The 825-bp insertion in pSHM06_2 covers the *CADPS2* intron 7 sequence encoded by the allele, in which insertion L1-dn13 occurred. The 825-bp insert in pSHM06_2 differs from the same size insertion of pSHM06_1 in the presence of insertion L1-dn13 (green) and the absence of the 202-bp and 203-bp sequences located at the 5' and 3' ends of the *CADPS2* intron sequence of pSHM06_1, respectively. pSHM06_2 differs from pSHM06_3 exclusively in the absence of L1-dn13 including one TSD sequence.**(b)** Nucleotide sequences of the three synthesized AflII/BstBI DNA fragments inserted into the AflII/BstBI site of pSHM06[2]**.** Red underlined ATG, start codon of E6-E7-Rluc-E6-E7 expression cassette. Bold underlined lettering following ATG, E6 sequence. Bold underlined lettering at the 3' end, E7 sequence. Bold black lettering, TPI intron 6. Blue highlighted sequence, *CADPS2* intron 7 sequence. Green highlighted sequence, L1-dn13 insertion. Red repetitive sequence, 16-bp L1-EN target recognition sequence (pSHM06_1, pSHM06_3) or TSDs (pSHM06_2), respectively. 825-bp and 422-bp *CADPS2* intron 7 sequences introduced into TPI intron 6 are highlighted in blue (pSHM06_1, pSHM06_3) or in blue and green (pSHM06_2). **(c)** Relative cell lysate luciferase activity measured 48 h post-transfection. *CADPS2* intron sequence harbouring L1-dn13 reduced luciferase activity by ~62 %. *CADPS2* intron sequences lacking L1-dn13 restrict luciferase activity by only 40-45 %. The difference in luciferase activities obtained after transfection of pSHM06_2 and pSHM06_1 is significant (p=0.022). Presented activities are an average of six independent experiments. Error bars represent arithmetic means ±s.d. RLU, relative light units. Luciferase activities obtained after transfection of pSHM05 (no TPI intron 6) and pSHM06 (with TPI intron 6) served as controls.

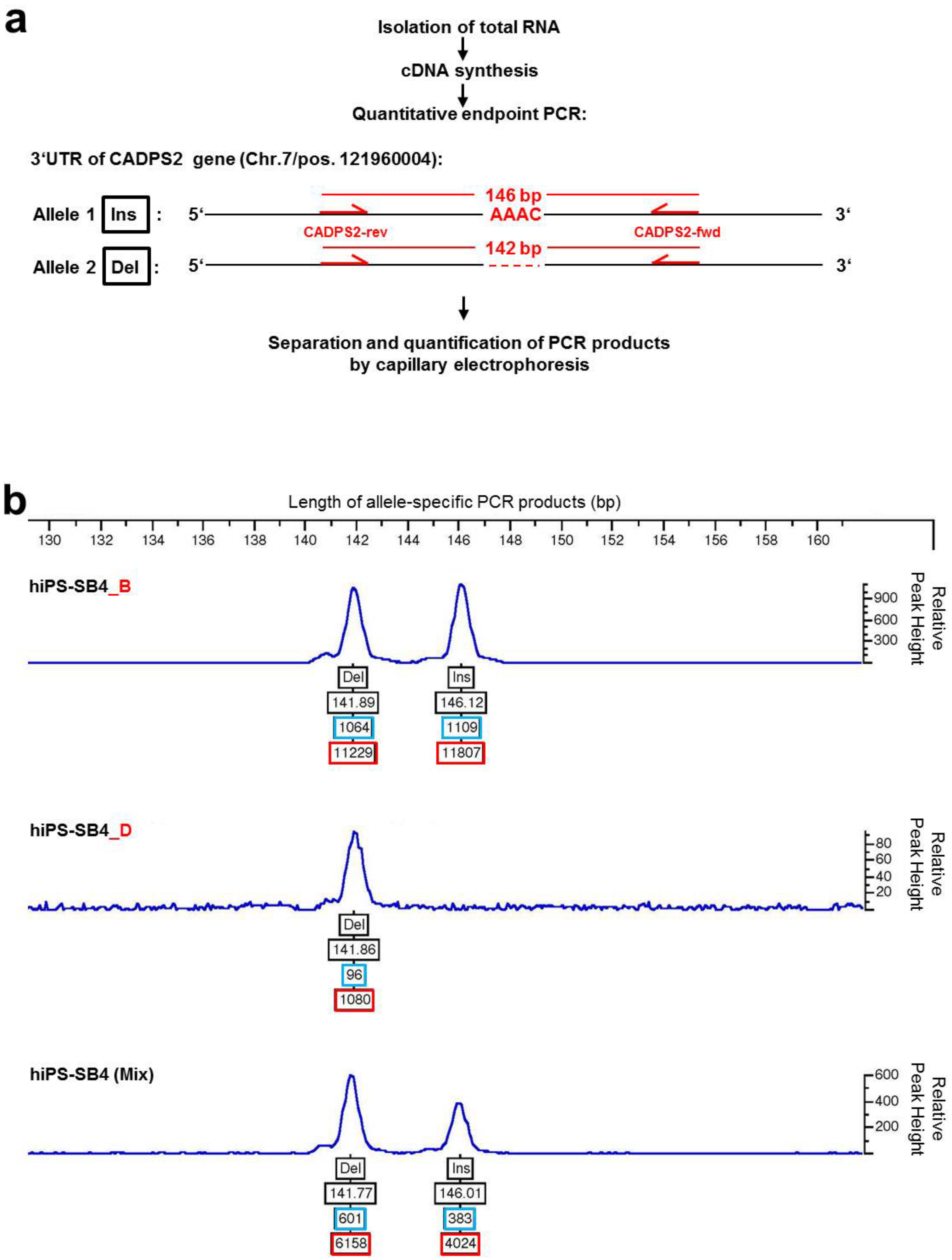
a
Isolation of total RNA
cDNA synthesis
Quantitative endpoint PCR:
3'UTR of CADPS2 gene (Chr.7/pos. 121960004):
Allele 1 Ins :
5'
146 bp
AAAC
3'
CADPS2-rev
CADPS2-fwd
Allele 2 Del :
5'
142 bp
3'
Separation and quantification of PCR products
by capillary electrophoresis
b
Length of allele-specific PCR products (bp)
130 132 134 136 138 140 142 144 146 148 150 152 154 156 158 160
hiPS-SB4_B
Relative Peak Height
900 600 300
Del
141.89
1064
11229
Ins
146.12
1109
11807
hiPS-SB4_D
Relative Peak Height
80 60 40 20
Del
141.86
96
1080
hiPS-SB4 (Mix)
Relative Peak Height
600 400 200
Del
141.77
601
6158
Ins
146.01
383
4024

Supplementary Figure 13: Quantification of allelic CADPS2 transcription in the hiPS-SB4(Mix) line and in single-cell subclones hiPS-SB4_B and hiPS-SB4_D.

(a) Schematic of the procedure to quantify allelic CADPS2 transcription. Endpoint quantitative RT-PCR was performed using primers CADPS2-fwd and CADPS2-rev which bind 43-62 bp upstream and 64-85 bp downstream of the annotated common SNP CAAA(-/AAAC)AAACA (UTR-variant-3-prime), respectively. For quantification, a calibration curve was generated by PCR with CADPS2-fwd and CADPS2-rev on mixtures of both linearized plasmids each harbouring one synthesized *CADPS2*-3'UTR allele including the SNP. Allele-specific PCR products obtained from hiPS-SB4(Mix), hiPS-SB4_B and hiPS-SB4_D cells and from the calibration curve experiments were separated and quantified by capillary electrophoresis.

(b) Capillary electrophoresis to quantify allelic CADPS2 expression in hiPSC lines with and without the intronic L1-dn13 insertion. Peaks result from the 146-bp PCR product derived from allele 1 including the AAAC SNP (Ins) and from the 142-bp PCR product derived from allele 2 devoid of the AAAC sequence at position 121960004/Chr 7 (genome build GRCh37/hg19). Peak heights (blue boxes) and peak areas (red boxes) are a measure for the relative amounts of allele-specific transcriptional products. Numbers in black boxes, lengths of respective PCR products in bp. X-axis, length of allele-specific PCR products in bp; Y-axis, relative peak heights.

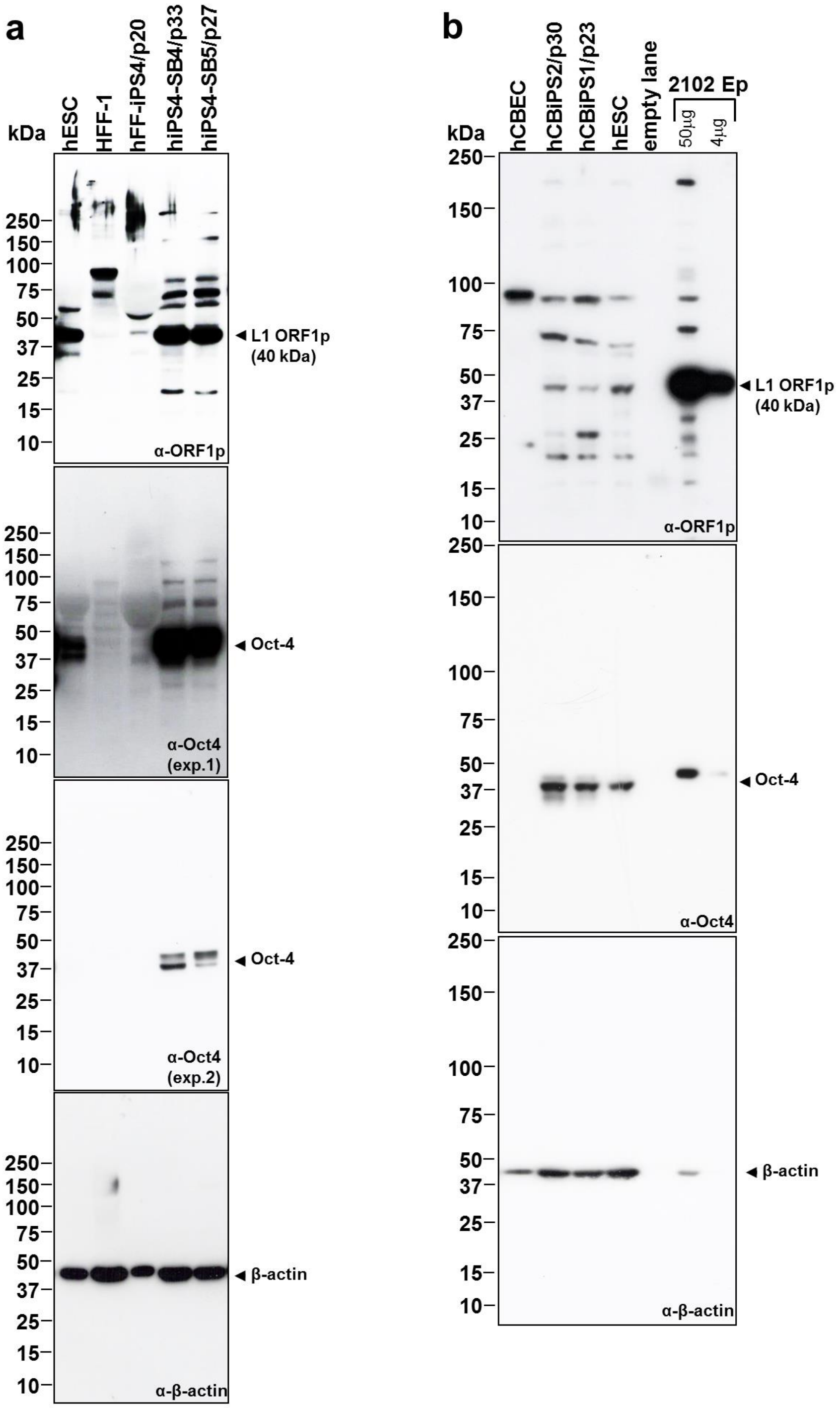

Supplementary Figure 14: Immunoblot analysis of cell lysates from HFF-1 (a) and hCBEC (b) cells and their respective derived hiPSC lines. Presented are full scans of the immunblots presented in Figure 1e. Analyses demonstrate L1 ORF1p (40 kDa) and Oct-3/4 expression (A isoform, 45 kDa; B isoform 33 kDa) in pluripotent stem cells. Shorter (exp.1) and longer exposures (exp.2) of the αOct-3/4 immunoblot are provided. Lysates from hESC lines HES-3 (a) and H1 (b) and the embryonal carcinoma line 2102 Ep (b) served as positive control for L1 ORF1p and Oct-3/4 expression. β-actin (42 kDa) was used as loading control.

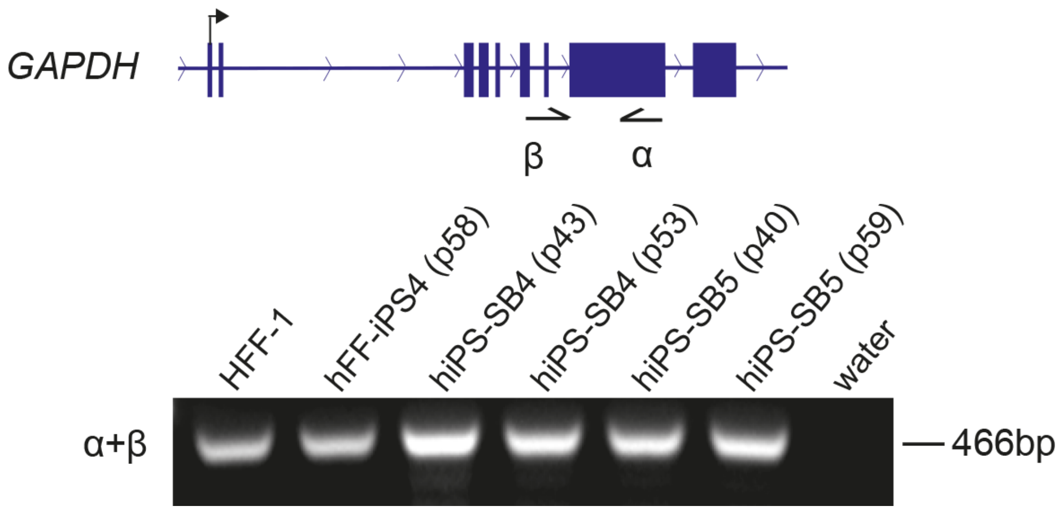

Supplementary Figure 15: Control PCR validation. As performed for the single-copy reference gene GAPDH in genomic DNA preparations of parental and iPSC lines used for RC-seq and PCR amplification of *de novo* insertions presented in Figure 3a. Primers GAPDH-α (Chr12: 6647005-6647026) and GAPDH- β (Chr12:6646561-6646580) are located in exon 8 and intron 7, respectively. The resulting PCR product spans 466 bp.

Supplementary Table 1: Applied primers and probes.

Primer/probe name	Target	Position in L1.3 or HERV-K reference	Annealing temperature (°C)	Product size (bp)	5'-3' Sequences
5´-UTR 1f	L1Hs	1021-1087	60	67	GAATGATTTTGACGAGCTGAGAGAA
5´-UTR 1r					GTCCTCCCGTAGCTCAGAGTAATT
5´-UTR 1p					AAGGCTTCAGACGATC
5´-UTR 2f	L1Hs	626-688	60	62	ACAGCTTTGAAGAGAGCAGTGGTT
5´-UTR 2r					AGTCTGCCCGTTCTCAGATCT
5´-UTR 2p					TCCCAGCACGCAGC
L1_FW1 (n)	L1Hs	58-1356		1299	AGCGACGCAGAAGACGGTGAT
L1_RV1 (n)					CCCATATTTCTTGGAGGCTTTGC
L1-13 f	L1-dn13	-40-107	60	147	AGCAGAAATGCATACAGAAAGG
L1-13 r					GGTTTCCAATTTCATCCATGTC
L1-13 p					ATATGTGCCACATTTTCTATGCAGCCA
L1-14 f	L1-dn14	-50-57	60	107	ACAGGGCAATAGAAAAACACC
L1-14 r					ACCGGAGCTGTTCCTATTC
L1-14 p					TATGATGAAAATACACAAGCACAGGGGGAGG
HK-forward	HERV-K HML-2	1626-1792	60	167	GGCCATCAGAGTCTAAACCACG
HK-reverse					CTGACTTTCTGGGGGTGGCCG
actin_FW (n)	Human β-actin gene			491	GCCGCCAGCTCACCATGGATG
actin_RV (n)					GACCCCGTCACCGGAGTCCA
OCT4 f	OCT4		60	60	CGACCATCTGCCGCTTTG
OCT4 r					GCCGCAGCTTACACATGTTCT
NANOG f	NANOG		60	62	CCA AAGGCA AACAACCCACTT
NANOG r					CGGGACCTTGTCTTCCTTTTT
SOX1 f	SOX1		60	97	CTGGCTGTGGCAAGGTCTTC
SOX1 r					CAGCCCTCAAACTCGCACTT
TUBB3 f	TUBB3		60	70	GGCCAAGTTCTGGGAAGTCA
TUBB3 r					CGAGTCGCCCACGTAGTTG
AFP f	AFP		60	182	AGCTTGGTGGTGGATGAAAC
AFP r					TCTGCAATGACAGCCTCAAG
GATA6 f	GATA6		60	178	AGGGTGAACCCGTGTGCAATG
GATA6 r					TGGAAGTTGGAGTCATGGGAATGG
EOMES f	EOMES		60	213	AGAGGGCTGTGCCTTCCGTTTC
EOMES r					AGCACACAGCAGAGGCCTAGCAAG
GAPDH f	GAPDH		60	60	ATGGAA ATCCCATCACCATCTT
GAPDH r					CGCCCCACTTGATTTTGG[1]

[1] Presented are sequences of oligonucleotides (f, r) and probes (p) used for quantitative real-time RT-PCR and normal PCR amplification (n), including corresponding gene information, primer position and applied PCR condition.

Supplementary Table 2: Applied antibodies

Species	Class	Name	Clonality	Vendor	Dilution
Rabbit	Rabbit IgG	Anti-L1ORF1p	polyclonal	Eurogentec [2]	1:2000 WB/ 1:250 IF
Mouse	Mouse IgG2b	Anti-Oct3/4	monoclonal	Santa Cruz	1:1000 WB/ 1:500 IF
Mouse	Mouse IgG1	Anti-CD105	monoclonal	Dako	1:500 IF
Goat	Goat IgG	AlexaFluor488 Anti-Mouse	polyclonal	Invitrogen	1:1000
Goat	Goat IgG	AlexaFluor647 Anti-Rabbit	polyclonal	Invitrogen	1:1000
Sheep	Sheep IgG	ECL-HRP coupled anti-Mouse	polyclonal	GE Healthcare	1:30.000
Donkey	Donkey IgG F(ab)´2 fragment	ECL-HRP coupled anti-rabbit	polyclonal	GE Healthcare	1:30.000
Mouse	IgG2b	Anti-Oct4	monoclonal	Santa Cruz Biotechnology	1:100
Mouse	IgM	Anti-SSEA-3	monoclonal	Hybridoma Bank, Iowa City, USA	1:100
Mouse	IgG3	Anti-SSEA-4	monoclonal	Hybridoma Bank, Iowa City, USA	1:70
Mouse	IgM	Anti-TRA-1-60	monoclonal	Abcam	1:100
Mouse	IgG2a	Anti-β-Tubulin	monoclonal	Upstate,NY, USA	1:400
Goat	Goat IgG	Anti-SOX17	monoclonal	R&D Systems	1:200
Mouse	IgG1	Anti-Troponin T	monoclonal	Thermo Scientific	1:100

Supplementary Table 3: Validated, reprogramming-induced retrotransposition events in HFF-1-derived hiPSC lines and hESC line H9

**ID**	**RC-seq information**					**PCR validation**		
	**Chr**	**Family**	**Strand**	**Read count**	**Detected**	**Primer α**	**Primer β**	**Detected**
L1-dn3	chr15	L1-Ta	-	10	hiPS-SB4-p43, hiPS-SB4-p53	CCAGCCTAATTCC TGTGACC	GTTGTTAACAGCA TAATGATCCAAA	hiPS-SB4-p43, hiPS-SB4-p53
L1-dn6	chr10	L1-Ta	-	5	hiPS-SB4-p53	GCAAAAATTTGGG AAAAGATTG	AACTCCCTGACCC CTTGC	hiPS-SB4-p43, hiPS-SB4-p53
Alu-1	chr15	*Alu*Yb8	-	9	hFF-iPS4-p58	GGAGTGCCTTGAA GGATGAG	CTGGCCTGCAATC ACTAGT	hFF-iPS4-p58
L1-dn10	chr4	L1-Ta	-	4	hiPS-SB4-p43	GATCCATGGCACA AAAATGA	AACTCCCTGACCC CTTGC	hiPS-SB4-p43, hiPS-SB4-p53
L1-dn14	chr11	L1-Ta	-	2	hiPS-SB4-p43	CAGGGCAATAGAA AAACACCA	AACTCCCTGACCC CTTGC	hiPS-SB4-p43, hiPS-SB4-p53
L1-dn15	chr11	L1-Ta	-	2	hiPS-SB5-p40	CTGTGGGGTACAT GTGCTTG	CACCAGCATGGC ACATGTAT	hiPS-SB5-p40, hiPS-SB5-p59
L1-dn4	chr5	L1-pre-Ta	-	3	hiPS-SB5-p59	CATCCCAATCTGA ACTACTGCAT	AGTGCACCAGCAT AGCACAT	hiPS-SB5-p40, hiPS-SB5-p59
L1-dn13	chr7	L1-Ta	-	2	hiPS-SB4-p53	GGATCCTTCTGTA TCCTAAATTCG (= primer ISP1)	CACTCTGGGGACT GTGGTG (= primer OP1)	hiPS-SB4-p53
Alu-7	chr4	*Alu*Ya5	+	14	hiPS-CRL1502-p15 hiPS-CRL1502-p40	TTGTTAGCCAGGA TGGT	GATATAGCTTGGG TGTTT	hiPS-CRL1502-p15
SVA-2	chr9	SVA_E	+	6	hiPS-CRL1502-p15 hiPS-CRL1502-p40	GGTCCTCTGCCTA GGAAAACCA	CAGAGGGTCAGTT CTACCCAG	hiPS-CRL1502-p15
Alu-2	chr10	*Alu*Ya5	+	3	H9-p60	TGCTCCAGACAG GTTTCTTCA	CAAGAACAGGCA GGGGAGAG	H9-p60

Supplementary Methods

HERV-K-specific qRT-PCR using SYBR® Green I dye.

To quantify transcripts levels of human endogenous retrovirus K group HERV-K(HML-2), *gag* specific primers listed in Table S1 were used as previously described[3]. qRT-PCR was performed with the Applied Biosystems 7900HT Fast Real-Time PCR System, FastStart SYBR Green Master Mix (Roche Applied Science, Mannheim, Germany) and the standard protocol. After 2 min incubation at 50°C, a 10-minute initial denaturation step at 95°C was followed by 40 amplification cycles of 15 s at 95°C and 60 s at 60°C. Dissociation curves were generated for the final PCR products by decreasing the temperature to 65°C for 15 s followed by an increase in temperature to 95°C. 18S rRNA transcripts (primers: 5'-GTCCCCCAACTTCTTAGAG-3' (for), 5'-CACCTACGGAAACCTTGTTAC-3'(rev)) were quantified as internal standard. The relative expression of HERV transcripts was calculated relative to HERV-K expression in human embryonic stem cell line HES-3, with values normalized to 18S rRNA. Quantitative RT-PCR experiments were performed for each gene in triplicate.

Immunoblot analysis with subsequent multiplexed detection of proteins using two infrared (IR) fluorophores.

20μg of each whole cell lysate were loaded on a 10% SDS-PAGE gel and transferred to nitrocellulose membranes following standard procedures. The blot was probed with rabbit anti-ORF1p (provided by Oliver Weichenrieder, Max-Planck Institute for Developmental Biology, Tübingen, Germany) at a dilution of 1:1000 and mouse anti-β-actin antibody (Sigma) at a dilution of 1:20,000. Next, we incubated membranes with fluorescent labeled antibodies (both at 1/10000) as follows: IRDye® 800CW Goat anti-Rabbit IgG (LI-COR P/N 926-32210) and IRDye 680LT Goat anti-Mouse IgG (LI-COR P/N 926-68020). IR fluorescence was measured and quantified using an Odyssey® device (LI-COR). We performed multiplexed detection of ORF1p and β-actin using green and redIR fluorophores simultaneously to quantify the rate of ORF1p/β-actin expression.

Generation of TPI/Renilla Luciferase reporter constructs to evaluate the effect of the L1-dn13 insertion on reporter gene expression.

The assay is predicated on a luciferase-based reporter system for monitoring the effects of individual introns on gene expression[2]. This reporter system is building on the plasmids pSHM05 and pSHM06 (Suppelmentary Fig. 12a,b)[2]. TPI exons 6 (E6; 85 nts) and 7 (E7; 38 nts) were cloned in frame at both ends of the Renilla luciferase ORF (no intron/pSHM05). In pSHM06, TPI intron 6 (127 nts) was inserted between exons 6 and 7 located at the 5' end of the expression cassette. An 825-bp sequence of *CADPS2* intron 7 in which insertion L1-dn13 occurred, was inserted into intron 6 of pSHM06 (pSHM06_1). Sequence-verified gene fragments carrying an 825-bp portion (Chr.7: 122,198,866 –122,198,041) and a 422-bp portion (Chr.7: 122,198,667 - 122,198,244) of intron 7 of the human *CADPS2* gene were generated by Custom Gene Synthesis (Integrated DNA Technologies, Coralville, IA, USA) as part of the E6-intron6-E7 cassette that is flanked by AflII and BstBI restriction sites in pSHM06 (Supplementary Fig. 12a,b). A third fragment was synthesized carrying the corresponding *CADPS2* intron 7 sequence of the allele of the hiPS-SB4 cell line, harbouring the 389-bp L1-dn13 *de novo* insertion (Supplementary Fig. 12a,b). Nucleotide sequences of each of the three AflII/BstBI fragments are presented in Supplementary Fig. 12b. To generate TPI/Renilla Luciferase reporter constructs pSHM06_1, pSHM06_2 and pSHM06_3 (Supplementary Fig. 12a), the 295-bp AflII/BstBI fragment in

pSHM06 was replaced by the 1121-bp, 1123-bp and the 718-bp AflII/BstBI-fragment, respectively, presented in Supplementary Fig. 12b. Sequences of the AflII/BstBI fragments in pSHM06_1, pSHM06_2 and pSHM06_3 were validated by sequencing analysis.

Luciferase reporter assays

Luciferase reporter assays were performed in the human cervical carcinoma cell line HeLa-HA[4] using the Dual Luciferase Reporter System (Promega) according to the manufacturer's instructions. $1x10^5$ HeLa HA cells were seeded per well in 12-well plates (Nunc) for 24 h at 37ºC. Subsequently, cells were transfected with 0.5µg of the TPI/Renilla Luciferase reporter plasmid pSHM05, pSHM06, pSHM06_1, pSHM06_2 or pSHM06_3 and 0.05µg firefly luciferase reference plasmid pSF-CMV-Fluc (Sigma-Aldrich) using the FuGENE HD transfection reagent (Promega) according to the manufacturer’s instructions. Each transfection was performed in sextuplicate. Light units were quantified using the microplate luminometer Infinite® 200 PRO (Tecan, Männedorf, Switzerland). The number of renilla luciferase light units was normalized according to manufacturer’s instructions. P-values for the pairwise comparisons of pSHM06_2 vs. pSHM06_1, and pSHM06_2 vs. vs. pSHM06_3 (with 95% CI) were adjusted for multiple comparisons according to Bonferroni. Statistical analysis was performed with SAS®/STAT software (PROCGLM), version 9.3, SAS System for Windows.

Quantification of allelic CADPS2 expression by endpoint quantitative PCR and capillary electrophoresis

The presence of the annotated common SNP CAAA(-/AAAC)AAACA (UTR-variant-3-prime) in the genomes of the analyzed hiPSC lines hiPS-SB4(Mix), hiPS-SB4_B and hiPS-SB4_D was confirmed by sequence analysis of CADPS2-3‘UTR-specific PCR products obtained using genomic DNA from the three lines as templates. 800ng total RNA per cell line were used as template to synthesize cDNA by applying the GoScriptTM Reverse Transcriptase system (Promega) according to the manufacturer‘s instructions. In order to facilitate quantification of allele-specific transcription by endpoint reverse transcription PCR[5], the AAAC-SNP harbouring 3‘UTR regions of both *CADPS2* alleles covering 146 bp and 142 bp (GRCh37/hg19; Chr. 7 Pos. 121959943-121960091), respectively, were synthesized and cloned into the plasmid pEX-A2 (Eurofins Genomics, Ebersberg, Germany) resulting in plasmids pEX-CADPS2-Insertion and pEX-CADPS2-Deletion. To standardize the quantification, DNA copies of both alleles, pEX-CADPS2-Deletion and pEX-CADPS2-Insertion, were mixed at various ratios (99/0, 0/100, 90/10, 50/50, 25/75, 10/90, 5/95). Both standard plasmid mixtures and hiPSC line-specific cDNAs were then used as template for endpoint PCR using primers CADPS2-fwd (5‘-CAAGCAGAGCCAAGGTTCAT-3‘) and CADPS2-rev (5‘-AGTAAGCCTGAATGTAGAGTA-3‘).

Endpoint quantitative PCR: 25 µl of PCR mixture contained 3µL of cDNA template (representing 15 % of the transcribed RNA) or 40,000 to 100,000 copies $µL^{-1}$ calibration plasmid DNA, 2.5 µl 10x Gold ST★R buffer (Promega, Madison), 1.5 U of AmpliTaq Gold DNA polymerase (ThermoFisher Scientific) and 0.2 µM each of CADPS2-fwd and CADPS2-rev primers. The 5‘ end of the forward primer was fluorescently labeled with 5-FAM. Thermal cycling conditions were as follows: initial denaturation of 12 min at 95°C; 35 cycles of 30 s at 95°C; 30 s at 59°C; 30 s at 72°C; and final extension of 10 min at 72°C. The PCRs were performed on a GeneAmp PCR system 9700 (Applied Biosystems). PCR products were diluted

at ratios 1:40, 1:100, and 1:500 and separated on POP-4™ separation matrix (ThermoFisher Scientific) by capillary electrophoresis using an Applied Biosystems 3500 Genetic Analyzer (LifeTechnologies). Electrophoresis and detection methods followed the manufacturer's technical manual. Samples were prepared for capillary electrophoresis using 1 µl RXN ILS 600 (Promega, Madison), 10 µl Hi-Di™ formamide (Life Technologies/Applied Biosystems), and 1 µl diluted sample. Injection conditions for the 3130*xl* Genetic Analyzer (Life Technologies/Applied Biosystems) instruments were 1.2 kV and 18s, running conditions were 15 kV and 1500 s. DNA profiles were analysed with GeneScan and Genotyper software (Life Technologies/Applied Biosystems).

*Calculation of Accuracy in Quantification of Allel-specific PCR product*s: In capillary electrophoresis methods, the quantity of an amplified PCR product is represented by peak area (measured in relative fluorescent units, rfu) or peak height. Accuracy in quantification of allele-specific PCR products was calculated by comparison of ratio of peak area or height of the amplicons of the two alleles generated from defined mixtures (90/10, 75/25, 50/50) of the two control plasmids containing the two variants of the SNP with the ratio of peak area or height of the amplicons from the cDNA.

Supplementary References

1. Sassaman, D. M., *et al*. Many human L1 elements are capable of retrotransposition. *Nat. Genet.*, **16**, 37–43 (1997).

2. Nott, A., Meislin, S. H., & Moore, M. J. A quantitative analysis of intron effects on mammalian gene expression. *RNA*. **9**, 607-17. (2003)

3. Diem,O., Schaffner,M., Seifarth,W., & Leib-Mösch,C. Influence of antipsychotic drugs on human endogenous retrovirus (HERV) transcription in brain cells. *PLoS. One.* **7**, e30054 (2012).

4. Hulme, A.E., Bogerd, H. P., Cullen B. R., & Moran J. V. Selective inhibition of Alu retrotransposition by APOBEC3G. *Gene*. **390**, 199-205 (2007)

5. Schmittgen, T.D., Zakrajsek, B.A., Mills, A.G., Gorn, V., Singer, M.J. and Reed, M.W. Quantitative reverse transcription-polymerase chain reaction to study mRNA decay: Comparison of endpoint and real-time methods. *Anal. Biochem.* **285,** 194-204 (2000)
